# A Nearly Complete Genome of *Ciona intestinalis* Type A (*C. robusta*) Reveals the Contribution of Inversion to Chromosomal Evolution in the Genus *Ciona*

**DOI:** 10.1093/gbe/evz228

**Published:** 2019-10-22

**Authors:** Yutaka Satou, Ryohei Nakamura, Deli Yu, Reiko Yoshida, Mayuko Hamada, Manabu Fujie, Kanako Hisata, Hiroyuki Takeda, Noriyuki Satoh

**Affiliations:** 1 Department of Zoology, Graduate School of Science, Kyoto University, Japan; 2 Department of Biological Sciences, Graduate School of Science, The University of Tokyo, Japan; 3 Ushimado Marine Institute, Faculty of Science, Okayama University, Setouchi, Japan; 4 DNA Sequencing Section, Okinawa Institute of Science and Technology Graduate University, Okinawa, Japan; 5 Marine Genomics Unit, Okinawa Institute of Science and Technology Graduate University, Okinawa, Japan

**Keywords:** genome, *Ciona intestinalis* type A (*C. robusta*), ascidian, chromosomal inversion

## Abstract

Since its initial publication in 2002, the genome of *Ciona intestinalis* type A (*Ciona robusta*), the first genome sequence of an invertebrate chordate, has provided a valuable resource for a wide range of biological studies, including developmental biology, evolutionary biology, and neuroscience. The genome assembly was updated in 2008, and it included 68% of the sequence information in 14 pairs of chromosomes. However, a more contiguous genome is required for analyses of higher order genomic structure and of chromosomal evolution. Here, we provide a new genome assembly for an inbred line of this animal, constructed with short and long sequencing reads and Hi-C data. In this latest assembly, over 95% of the 123 Mb of sequence data was included in the chromosomes. Short sequencing reads predicted a genome size of 114–120 Mb; therefore, it is likely that the current assembly contains almost the entire genome, although this estimate of genome size was smaller than previous estimates. Remapping of the Hi-C data onto the new assembly revealed a large inversion in the genome of the inbred line. Moreover, a comparison of this genome assembly with that of *Ciona savignyi*, a different species in the same genus, revealed many chromosomal inversions between these two *Ciona* species, suggesting that such inversions have occurred frequently and have contributed to chromosomal evolution of *Ciona* species. Thus, the present assembly greatly improves an essential resource for genome-wide studies of ascidians.

## Introduction

The genome of the ascidian, *Ciona intestinalis*, was decoded in 2002 as the seventh animal genome (Dehal et al. 2002). Recently, it was shown that there are two cryptic species of *C.intestinalis*, types A and B (Caputi et al. 2007; Nydam and Harrison 2007, 2010, 2011a, 2011b; Sato et al. 2012, 2014; Roux et al. 2013; Bouchemousse et al. 2016). A taxonomic study (Brunetti et al. 2015) proposed renaming *C.intestinalis* type A as *Ciona robusta* and *C.intestinalis* type B as *C.intestinalis*. This newly proposed nomenclature is sometimes confusing, especially in studies using genomic information, because the animal from which the genome was decoded (Dehal et al. 2002) was originally identified as *C. intestinalis*. To avoid such confusion, many reports have included two names to identify the species, such as *C.intestinalis* type A (*C.robusta*) (e.g., Yoshida et al. 2017; Satoh et al. 2018; Cao et al. 2019; Kourakis et al. 2019; Matsubara et al. 2019; Mizutani et al. 2019; Oonuma and Kusakabe 2019). For the sake of clarity, the genome that was further explored in this study was from *C.intestinalis* type A (*C.robusta*).

Ascidians are tunicates, the closest relatives to vertebrates (Delsuc et al. 2006; Putnam et al. 2008). The ascidian tadpole-like larva, which comprises only 2,600 cells, shares the basic body plan of vertebrates. The larval tail contains a central notochord flanked laterally by muscle, dorsally by nerve cord, and ventrally by endodermal cells (Satoh 2003; Lemaire 2011). The genome sequence has been utilized as a key resource to analyze developmental mechanisms underlying such a simple body plan, especially genome-wide gene regulatory networks, epigenetic regulatory mechanisms, and gene expression profiles at single-cell resolution (Imai et al. 2004, 2006; Suzuki et al. 2007; Horie et al. 2018; Cao et al. 2019; Madgwick et al. 2019). Thus, the genome sequence, in combination with more than a century of experimental animal studies (Conklin 1905a, 1905b), has made *Ciona* an ideal model system for studies of developmental mechanisms (Satoh 2013), and the origin and evolution of chordates and vertebrates (Satoh 2016). For example, recent studies have shown that ascidian embryos develop cells similar to placodal cells and neural crest cells in vertebrate embryos (Manni et al. 2004; Mazet et al. 2005; Jeffery et al. 2008; Tresser et al. 2010; Abitua et al. 2012, 2015; Wagner and Levine 2012; Ikeda et al. 2013; Stolfi et al. 2015; Waki et al. 2015; Horie et al. 2018).

Chromosomal-level genome sequence data for *C.intestinalis* type A (*C. robusta*) became available after a major update in 2008 (Satou, Mineta, et al. 2008). This version, called the KH assembly (Kyoto-Hoya; “Hoya” is a Japanese word for ascidians), includes 68% of the sequence information in 14 pairs of chromosomes. Recent technological advances enabled us to analyze higher-order structure of the genome, and motivated us to improve the quality of the *Ciona* genome assembly.

Comparisons of invertebrate genomes have shown that orthologous sequences on an ancestral chromosome tend to be retained in its descendant chromosome of extant taxa, but the order of orthologous sequences is generally not conserved (Clark et al. 2007; Hillier et al. 2007; Hill et al. 2008). On the other hand, in vertebrate genomes, interchromosomal rearrangements are more common (Waterston et al. 2002; Kasahara et al. 2007). To analyze at higher resolution how chromosomes have changed during evolution, chromosomal-level genome sequences will undoubtedly be helpful.

In the present study, we provide a new assembly, called the HT (Hoya T-strain)-version. This assembly contains 95% of the genome sequences in the chromosomes; thus, it provides a valuable genomic resource for chordate studies. Comparison of this assembly with the genome of *Ciona savignyi*, which is a different species in the same genus, allowed us to identify many chromosomal inversions between the two species.

## Materials and Methods 

### Biological Materials

In the present study, we used *C.intestinalis* type A (*C. robusta*). To confirm that the animal we used was *C. intestinalis* type A, we used genomic sequences of five loci, *Fgf4/5/6* (this gene annotation was likely incorrect, because the sequences found in the public database were all mapped to a region within an intron of the *Fgf receptor* gene; chromosome 4: 7,098,700–7,099,456), *Foxa.a* (*fkh*; chromosome 11: 7,730,157–7,731,071)*, Jade* (chromosome 2: 4,786,572–4,787,267)*, Patched* (chromosome 5: 4,938,586–4,939,428), and *Vesicular acetylcholine transporter* (*vAChTP*; chromosome 1: 5,619,869–5,620,532), because these loci have been reported to be diverged between these two types (Nydam and Harrison 2011a). Sequences retrieved from NCBI were aligned using the Clustal Omega program (Sievers et al. 2011), and alignments were manually adjusted. After removing gaps, alignments were used to construct molecular phylogenetic trees by the maximum likelihood method with the PhyML program (Guindon and Gascuel 2003). Trees were tested with 100 bootstrap pseudoreplicates. All molecular phylogenetic trees for these five loci indicated that the animal we used was *C. intestinalis* type A (*C. robusta*) (supplementary fig. S1, Supplementary Material online).

### Genome Sequencing

For PacBio RSII sequencing, we used sperm obtained from an animal in the eighth generation of self-fertilization, as described previously (Satou et al. 2015). To prepare a library, an SMRTbell Template Prep Kit 1.0 (Pacific Biosciences) was used. The library was sequenced using a PacBio RSII sequencer employing P6-C4 chemistry (Pacific Biosciences) with 360-min movie lengths. Contig assembly was performed with the MECAT assembler pipeline (Xiao et al. 2017). Each program was run with the following parameters: for mecat2pw, “-j 0”; for mecat2cns, “-i 0”; for extract_sequences, “40× 160000000”; for mecat2canu, “genomesize = 160000000.”

For polishing contig sequences obtained with the MECAT assembler, Pilon was utilized (Walker et al. 2014). Illumina sequencing reads (paired 101 base reads, 16.6 Gb in total; SRA accession number DRR018354) for a 11th generation animal (Satou et al. 2015) were mapped with bowtie2 (Langmead et al. 2019).

### Hi-C

Hi-C (*in situ* Hi-C) experiments were performed as previously described (Rao et al. 2014) with some modifications. Approximately 1,000 tailbud embryos were collected by centrifugation at 500 × g for 3 min, and cross-linked with 1% formaldehyde in PBS for 10 min at room temperature. Cross-linking was quenched by adding 2.5 M glycine (125 mM final) and incubating for 5 min at room temperature, followed by 15 min on ice. Cells were pelleted at 500×g for 5 min. Supernatant was removed and stored at −80 °C.

Cells were thawed on ice, washed with PBS, resuspended in 250 μl of ice-cold Hi-C lysis buffer (10 mM Tris–HCl pH 8.0, 10 mM NaCl, 0.2% Igepal CA-630) with 50 μl of protease inhibitors (Sigma, P8340), and incubated on ice for 20 min. The lysate was centrifuged at 2,500 × g for 5 min, washed with ice-cold Hi-C lysis buffer, resuspended in 50 μl of 0.5% SDS, and incubated for 10 min at 62 °C. Then, 145 μl of water and 25 μl of 10% Triton X-100 were added. After incubation for 15 min at 37 °C, 25 μl of NEBuffer2 and 100 U of MboI were added, and chromatin was digested overnight at 37 °C with rotation. MboI was inactivated by incubating at 62 °C for 20 min. DNA ends were labeled with biotin by adding 50 μl of fill-in master mix (0.3 mM biotin-14-dATP, 0.3 mM dCTP, 0.3 mM dGTP, 0.3 mM dTTP, 40 units of DNA Polymerase I Large Klenow Fragment), and incubated at 37 °C for 1.5 h with rotation. Proximal ligation was performed by adding 900 μl of ligation master mix [669 μl of water, 120 μl of 10× NEB T4 DNA ligase buffer, 100 μl of 10% Triton X-100, 6 μl of 10 mg/ml BSA, and 5 μl of 400 U/μl T4 DNA ligase (NEB)], and incubated at room temperature for 4 h. Proteins were degraded by adding 50 μl of 20 mg/ml proteinase K, 120 μl of 10% SDS, and incubated at 55 °C for 2 h. Cross-linking was reversed by adding 130 μl of 5 M NaCl and incubated at 68 °C overnight.

Biotinylated DNA was collected by ethanol precipitation, and resuspended in 130 μl of Tris buffer (10 mM Tris–HCl, pH 8.0). DNA was sheared using Covaris S220 with following parameters; Peak Incident Power: 140, Duty Factor: 10, Cycle per Burst: 200, time: 80 s. Sheared DNA was size-selected to 100–500 bp and purified using AMPure XP beads (Bechman Coulter). DNA was eluted in 100 μl of Tris buffer.

A 150 μl of 10 mg/ml Dynabeads MyOne Streptavidine T1 beads (Life technologies) were washed with 400 μl of 1× Tween washing buffer (1× TWB: 5 mM Tris–HCl pH 7.5, 0.5 mM EDTA, 1 M NaCl, 0.05% Tween 20), and resuspended in 300 μl of 2× binding buffer (2× BB: 10 mM Tris–HCl pH 7.5, 1 mM EDTA, 2 M NaCl). The beads were added to the sheared DNA sample, and incubated at room temperature for 15 min with rotation. Biotinylated beads with bound DNA were collected with a magnet and supernatant was discarded. The beads were washed twice by adding 600 μl of 1× TWB, transferred to a new tube, incubated at 55 °C for 2 min on a Thermomixer. The supernatant was discarded using a magnet. Then, beads were washed with 100 μl of Tris buffer, transferred to a new tube, and resuspended in 50 μl of Tris buffer.

A-tailing and Illumina adapter ligation were performed using the KAPA Hyper Prep Kit (Kapa Biosystems KK8500). Adapter-ligated DNA was washed twice more by adding 600 μl of 1× TWB, transferred to a new tube, and incubated at 55 °C for 2 min on a Thermomixer. The supernatant was discarded using a magnet to retain the beads. Beads were washed with 100 μl of Tris buffer, transferred to a new tube, and resuspended in 50 μl of Tris buffer. Libraries were amplified directly from the beads with 9–14 cycles of PCR, using KAPA HiFi HotStart ReadyMix (KAPA Biosystems), and DNA was purified using AMPure XP beads. Paired-end sequencing of Hi-C libraries was performed using the Illumina HiSeq 1500 platform. Sequenced reads were mapped, filtered, and normalized using Juicer (version 1.5) (Durand et al. 2016). Hi-C data were analyzed with the 3D de novo assembly (3D-DNA) pipeline (Dudchenko et al. 2017) to obtain candidates for novel linkages and misassembly locations in the former KH version assembly.

To validate these candidates, we used Illumina paired sequencing data obtained in a previous study (Satou et al. 2015) (SRA accession number DRR018353 and DRR018354) (supplementary fig. S2, Supplementary Material online), and BAC end sequence data (Dehal et al. 2002; Kobayashi et al. 2002). We inspected Hi-C data using the Hi-C data browser (Durand et al. 2016) to obtain scaffolds, which are tentatively called KH/Hi-C linked scaffolds.

**Fig. 1.**
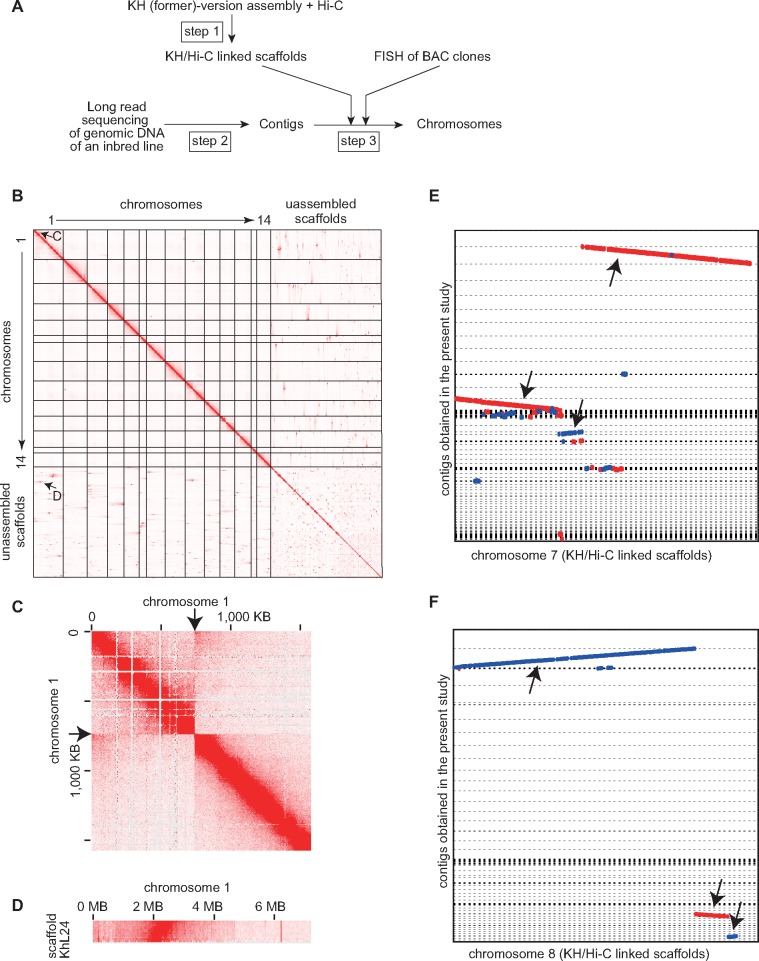
—Assembly of the genome of an inbred strain. (*A*) The assembly strategy. See supplementary figure S2, Supplementary Material online for details. (*B*) Hi-C data mapping on the KH-version of chromosomes and scaffolds for identification of candidates for misassemblies and linkages. (*C*) A candidate for an artifactitious inversion site in chromosome 1 of the KH-version of the assembly. Note a clear disconnection at the point indicated by arrows. The Hi-C data demonstrate the proximity between the initial ∼200 kb region and the region around 750 kb, suggesting that the initial ∼750 kb region is inverted. (*D*) A candidate for a possible linkage between chromosome 1 and scaffold KhL24. Chromosomes 7 (*E*) and 8 (*F*) are probably partly heterozygous in the animal used for PacBio RSII sequencing. Arrows indicate contigs used for genome assembly. These sequences were aligned with Nucmer and visualized with Mummer Plot (Kurtz et al. 2004). Forward alignments are shown in red and reverse alignments are shown in blue.

### Chromosome Assembly

Contigs obtained from sequencing data were aligned with the KH/Hi-C linked scaffolds using Nucmer (Kurtz et al. 2004). For possible heterozygous regions on chromosomes 7 and 8, only longer contigs were kept for the following assembly processes. Contigs were compared with KH/Hi-C linked scaffolds using BLASTN (Altschul et al. 1990) with the options “-task megablast -perc_identity 95.” The top hits with 1-kb or longer alignments were used as inputs for Chromosomer to link the contigs (Tamazian et al. 2016). Finally, nucleotide sequences of both ends of BAC clones used for fluorescence in situ hybridization (Shoguchi et al. 2006) were mapped with BLAT (Kent 2002). On the basis of mapping data, six contigs were included in the final chromosomal sequences. We inserted 1,000 “N”s between contigs, and as a result, 40,000 “N”s are included in the final assembly. Note that the assembly included two additional “N”s, which are bases we failed to determine.

### Genome Size Estimation

For genome size estimation, we caught animals in Ushimado, Okayama Prefecture, Japan. From two animals, we obtained a sufficient number of sequencing reads (∼4 Gb). All possible 21-mers were counted using Jellyfish (Marcais and Kingsford 2011), and genome sizes for each individual were estimated with Genomescope (Vurture et al. 2017).

### Gene Prediction

For predicting genes/transcripts, we used Augustus (Stanke et al. 2008) with cDNA sequences available in the DDBJ/EMBL/Genbank database and the KH version of the gene model set as hints. Predicted models were inspected on the Artemis browser (Carver et al. 2012), and manually curated on the basis of mapped cDNA-based data (full cDNA sequences and expression sequencing tags). Transcript names consist of five fields delimited by dots (e.g., KY.Chr1.1.v1.nonSL1-1). The first field represents the gene/transcript model version; therefore, all models have the same tag (KY stands for Kyoto). The second field represents the chromosome (or unassembled contig) name. The third name-field represents the serial number for gene loci on individual chromosomes. Gene models are defined with these first three fields (KY.Chr1.1). The fourth field specifies alternative transcript variants by numbers preceded by the letter “v.” The fifth field includes information for the 5′- and 3′-ends of the models, and consists of two subfields delimited with hyphens. The first subfield refers to the evidence identifying the 5′-end: SL means *trans*-splice acceptor site defined experimentally, nonSL means non-*trans*-spliced mRNA 5′-end determined experimentally, and ND means 5′-end predicted computationally (not determined by experimental evidence). The number concatenated to the 5′-end code identifies individual alternative 5′-ends within each locus. The second subfield refers to the 3′-end and consists of numbers identifying individual alternative 3′-ends within each locus.

### Experimental Validation of the Inversion in Chromosome 4

**Fig. 2.**
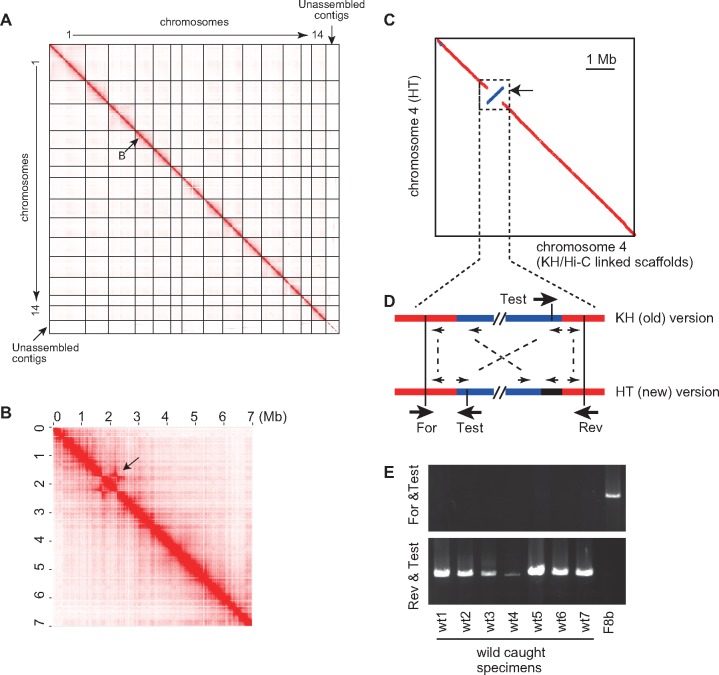
—An inversion in chromosome 4. (*A*) Hi-C data mapping on the new HT-version of the assembly. Mapping data show an overall high level of consistency, except for a small region in chromosome 4 (shown by *B*). (*B*) Hi-C mapping data for chromosome 4 of the HT version of the assembly. Note that the assembly is based on genomic DNA derived from the T-inbred line, and that Hi-C data were obtained from embryos derived from wild-caught animals. A possible inversion is indicated with an arrow. (*C*) The Nucmer alignment (Kurtz et al. 2004) of chromosome 4 of the HT version assembly with the corresponding region of KH/Hi-C linked scaffolds. A PCR experiment to confirm the inversion. (*D*) Three primers were designed, and their locations and orientations are shown by large arrows. Small arrows indicate genes, and the same genes are linked with broken lines. Note that the region indicated by the black line in HT does not have a corresponding region in the KH version. (*E*) Two sets of primers were used to examine which set gave specific amplification. PCR products were analyzed by agarose gel electrophoresis. The set of For and Test gave specific amplification for F8b, whereas the set of Rev and Test gave specific amplification for the seven wild-caught animals.

We used genomic DNAs obtained from seven wild-caught animals. These DNAs were isolated in our previous study, in which we called these specimens wt1 to wt7 (Satou et al. 2015). To examine this inversion, we performed PCR. The experimental design is shown in figure 2*D*. Primer sequences are as follows: For, 5′-ACGTAGGAGATCCAAATCAAAGCCATCATA-3′; Rev, 5′-ACCCACAGTAACCTATGATAAACGACTACTT-3′; Test, 5′- CTATCACACAAGAGATATGCACAAAGCATA-3′. PCR was performed with Primestar Gxl enzyme according to manufacturer instructions (Takara Bio).

### A Comparison of Gene Model Sets Between Two *Ciona* Species

*Ciona* *savignyi* protein sequences were obtained from the Ensembl database (Zerbino et al. 2018). Genomic positions of genes encoding these proteins were also deduced from the same database, and these genes were ordered within each reftig. *Cionasavignyi* proteins were used as queries for BLAST searches against proteins derived from KY gene models. Because we compared 11,616 proteins, hits with E-values <4.3e^−6^ (0.05/11616; Bonferroni correction for multiple testing) were regarded as significant. For conservative comparisons, we used only hits in which the alignment exceeded 40% of query and subject protein lengths. Species-specific tandem duplications may affect subsequent analyses; therefore, we used only the highest scoring match if two or more *C. savignyi* proteins were mapped to a single KY model. Reftigs that contained 10 or more genes with putative orthologs among the KY gene models and had orthologs preferentially in 1 of the 14 chromosomes (Fishers exact tests with the Bonferroni correction <5%/122) were chosen to make a dot plot.

**Fig. 3.**
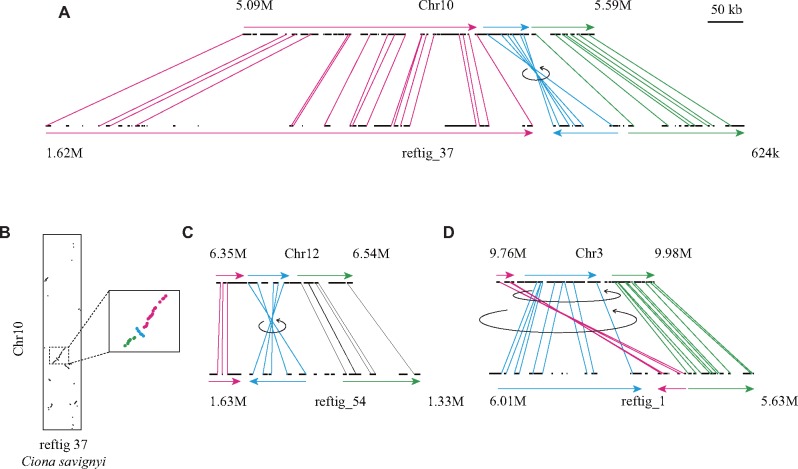
—Possible inversions between chromosomes of two *Ciona* species. Genes of the two *Ciona* species are shown as black lines in the upper (HT chromosomes) and lower rows (*C. savignyi* reftigs) along genomic regions indicated above and below the rows. Colinear genomic blocks are shown with colored arrows, and 5′-ends of putative orthologous genes are linked by lines of the same color. Putative inversions are indicated with black arrows. Single inversions can explain the gene arrangements in (*A*) and (*C*). Two inversions can explain the gene arrangement in (*D*). (*B*) A dot plot represents the rank order position of orthologous gene pairs in chromosome 10 in the HT-assembly and reftig 37 of *C. savignyi*. The region shown in a higher-magnification view includes genes shown in (*A*).

## Results

### A Strategy for Constructing a New Assembly

To construct a new version of genome assembly, we employed the strategy shown in figure 1*A* (see also supplementary fig. S2, Supplementary Material online). First, we rebuilt scaffolds using contigs from the former KH assembly and Hi-C data, and the resulting scaffolds were further curated with Illumina sequencing reads. Second, independently of the first step, we assembled long-read sequencing data from a PacBio sequencer. Third, we combined these two types of data with fluorescent in situ hybridization (FISH) data from BAC clones (Shoguchi et al. 2004, 2006) to obtain a final assembly called the HT assembly.

### Step 1: Constructing Reference Scaffolds from Scaffolds of the Previous Version Using Hi-C Data

We performed an Hi-C analysis using tailbud embryos to determine distances between nucleotide positions within chromosomes. The KH version assembly contains a total of 115,226,814 bases, 68% of which are mapped to the 14 chromosomes. Mapping of the Hi-C data suggested many possibly misassembled sites and potential linkages (fig. 1*B–D*). We first inferred positions of unassembled scaffolds and misassembled positions within the chromosomes using the 3D-DNA pipeline (Dudchenko et al. 2017). These candidates were individually validated as follows.

We mapped Illumina sequencing data obtained from a previous study (Satou et al. 2015) (supplementary fig. S2*B*, Supplementary Material online) to examine whether these candidate positions were supported by paired-end sequences. Similarly, we mapped sequence data of both insert ends of BAC clones, which were derived from Sanger sequencing (Dehal et al. 2002; Kobayashi et al. 2002). For each of the candidates supported by any of these sequencing data, we inspected Hi-C data using the Hi-C data browser (Durand et al. 2016). As a result, we obtained a new set of 14 chromosomal sequences containing 95,495,880 bases. This set of chromosomal sequences was used as references for the new assembly (see below). Hereafter, these reference scaffolds are tentatively called KH/Hi-C linked scaffolds.

### Step 2: Primary Assembly Using Long Reads

*Ciona* is a hermaphroditic animal, and self-fertilization can be induced in the laboratory. Taking advantage of this, we created an inbred line (T-line) by repeated self-fertilization (Satou et al. 2015). Genomic DNA from sperm obtained from a specimen in the eighth generation was used. The heterozygosity rate in natural populations is expected to be 1.1–1.2% (Dehal et al. 2002; Satou et al. 2012). Under the assumption of neutrality, the heterozygosity rate for the eighth generation inbred animals was expected to be 0.0043–0.0047% [1.1–1.2% × (1/2)^8^], and we considered this rate sufficiently low. Using this specimen, we obtained 644,697 sequence reads from a PacBio RSII sequencer, which yielded 7,946,426,251 bases (supplementary fig. S2*B*, Supplementary Material online). Using the MECAT assembler (Xiao et al. 2017), we reconstructed 126,262,676 bases into 153 contigs (N50 = 3,746,906; L50 = 12). These sequences were further polished with Pilon (Walker et al. 2014) and Illumina sequencing data from a specimen in the 11th generation (supplementary fig. S2*B*, Supplementary Material online), which corrected 31,314 sites.

### Step 3: Scaffolding the New Contigs

Next, we compared the new contigs mentioned above with the KH/Hi-C linked scaffolds using Nucmer (Kurtz et al. 2004) (supplementary fig. S3, Supplementary Material online). This alignment revealed duplicated regions in chromosomes 7 and 8 (fig. 1*E* and *F*). These contigs probably represent haplotypes, because the duplicated region in chromosome 7 overlapped the region that is retained as heterozygous even in the 11th generation (Satou et al. 2015). This region contains a self-incompatibility locus with a highly variable region among individuals to prevent fertilization between eggs and sperm with the same locus type (Harada et al. 2008). We chose more contiguous contigs for these regions for the following assembly processes (fig. 1*E* and *F*). These contigs and contigs that aligned to other chromosomes (47 contigs in total) were assembled into 14 chromosomes in reference to the KH/Hi-C linked scaffolds, using Chromosomer (Tamazian et al. 2016). Nevertheless, 60 contigs remained unassembled into chromosomes.

Finally, we mapped sequences of both insert ends of 270 BAC clones that were used for previous FISH assays (Shoguchi et al. 2004, 2006). Sequences of 18 BAC clones were mapped onto seven unassembled contigs with BLAT (Kent 2002), and the FISH results indicated that these contigs were located at chromosomal ends. As a result, we included these seven contigs in chromosomal sequences, and the number of unassembled contigs was reduced to 53. Linkages that were determined on the basis of the KH/Hi-C linked scaffolds and FISH are listed in supplementary table S1, Supplementary Material online.

The final assembly included 122,951,598 bases (table 1). Among them, 117,489,544 bases (95.6%) were included in the chromosomal sequences. The N50 was 8,327,059 bases and the L50 was 6. We called this the HT assembly, a great improvement compared with the KH assembly, as evident from their statistics (table 1).

**Table 1:** Basic Statistics of the Present and Previous Assemblies

	HT Assembly (Present)	KH Assembly (Previous)
Total nucleotide length (bp)	122,951,598	112,162,187
Total nucleotide length including “N” length (bp)	122,991,600	115,226,814
Number of chromosomes	14	14
Number of contigs/scaffolds that are not included in chromosomes	53	1,258
N50 (bp)	8,327,059	5,152,901
L50	6	9
N90 (bp)	4,872,821	40,806
L90	13	196

### Gene Models

We utilized Augustus (Stanke et al. 2008) to build a new set of gene models, using cDNA sequences and the KH gene models as hints. The resulting models were manually curated with Artemis (Carver et al. 2012). We identified transcription start sites (TSSs) and *trans*-splicing accepter sites for the 5′-spliced leaders (SL sites) using the data obtained through various high-throughput methods (Satou et al. 2006; Matsumoto et al. 2010; Yokomori et al. 2016). Using these data, 5′-ends of models were extended or shortened. Our previous study showed that there are no intergenic regions in any operon, and that polycistronic transcripts derived from operons are resolved by *trans*-splicing (Satou et al. 2006). Therefore, we curated every intergenic region within operons and checked whether upstream genes ended with “AG,” which worked as a *trans*-splicing acceptor site for downstream genes.

The resultant model set [KY (KYoto) model set] contains 14,072 genes with 18,701 splicing variants in total. When multiple 5′-ends (TSSs and/or SL acceptor sites) or 3′-ends were indicated by experiments for a single splicing variant, multiple transcript models were constructed. As a result, 61,667 transcript models were constructed; among them, 22,239 transcript models start with TSSs, and 31,757 transcript models start with SL sites. Among these transcript variants, 14% were not included in the KH models. Similarly, 9% were not included in the Refseq model set (O’Leary et al. 2016) (supplementary fig. S4, Supplementary Material online).

### Validation of the Assembly

We again mapped sequences of both insert ends of the BAC clones described above onto the final assembly. Among the 270 BAC clones, one or both ends of 257 clones were confidently mapped (BLAT score ≥150; single hits) (supplementary table S2, Supplementary Material online). Most of these clones (254 of 257) were mapped onto the expected chromosomes in the expected order. Only three clones showed inconsistent results. More specifically, one end of a clone (GECi31_c16) was mapped onto chromosome 8, which was supported by FISH results, but the other end of this clone was mapped onto chromosome 10, which was not supported by FISH result. Similarly, one end sequence for each of two clones (GECi38_g14 and GECi47_f07) was mapped near one end of chromosome 8, and the other end was mapped to unassembled contigs. We were not able to determine whether these three clones indicate actual misassemblies or whether they artificially contained two genomic fragments. Nevertheless, as mentioned above, a vast majority of the clones were mapped onto the expected chromosomes. Therefore, it is unlikely that the new assembly contains any large misassemblies.

Next, Hi-C data were mapped onto the new version of the genome (fig. 2*A*). The overall contiguity of chromosomes was much improved (compare fig. 2*A* with fig. 1*B*). We found no obvious inconsistency except for one position in chromosome 4 (fig. 2*B*), where the inbred strain indeed had an inversion (see below). In other words, except for this region, the Hi-C data indicated that the overall structure of the assembly successfully reproduces the chromosomal structures.

To evaluate nucleotide level errors, we compared the assembled sequence with the KH version of the genome sequence. To do this, we split the KH genome sequences into 222,119 fragments, each of which was 500 bases in length. These were used as query sequences for the BLAT program (Kent 2002). Among them, 178,398 fragments were mapped uniquely onto the new assembly, and 88,559,623 bases were aligned in total. This alignment contained 974,264 mismatches, corresponding to 1.1% (=974,264/88,559,623) in excellent agreement with the heterozygosity rate of natural populations of this animal (1.1–1.2%). Therefore, it is likely that the new assembly does not contain significant errors at the nucleotide level.

Finally, we evaluated the genome and gene models using BUSCO, a tool for assessing completeness of genome assemblies and gene models with single-copy orthologs (Simao et al. 2015). For this purpose, we used the metazoan gene model set distributed with BUSCO. Among the “metazoan” genes, 95.3% were found in the genome. This score was slightly improved when compared with the score of the previous KH assembly (94.2% found) (table 2). Similarly, BUSCO gave a slightly better score for the KY gene model set than for the former KH version set (table 2). This observation suggests that the HT assembly mainly improved genomic regions that do not encode protein-coding genes.

**Table 2:** Evaluation of Present and Previous Versions of the Assemblies and the Gene Model Sets Using BUSCO

	Found		Missing
	Complete	Fragmented	
Genome			
HT assembly (new)	94.6%	0.7%	4.7%
KH assembly (old)	93.0%	1.2%	5.8%
Gene models			
KY models (new)	95.6%	1.3%	3.1%
KH models (old)	95.0%	1.7%	3.3%

For an independent validation, we mapped 318 genes for transcription factors and signaling ligand molecules (supplementary fig. S5, Supplementary Material online). These genes constitute a set of the most extensively annotated genes, including all family members encoding bHLH, bZip, Ets, Fox, HMG-box, homeodomain, and nuclear receptor transcription factors, and all family members encoding Fgf, Ephrin, Tgfβ/Bmp, Wnt, hedgehog, and Notch ligands (Hino et al. 2003; Wada et al. 2003; Yagi et al. 2003; Yamada et al. 2003; Satou, Imai, et al. 2003; Satou, Sasakura, et al. 2003; Satou and Satoh 2005; Satou, Wada, et al. 2008). It also includes genes that encoded well-known transcription factors such as Zic, Prdm1, and Snai. Among these genes, only one (*Fgf4/5/6*) was included in an unassembled contig. This indicates that most protein coding genes are included in chromosomal sequences.

The KH version of the assembly predicted that several transcription factor genes, important for fate specification in embryos, are multicopy genes. However, precise genomic structures and copy numbers of *Foxd*, *Tbx6-r.b*, and *Zic-r.b* (formerly *ZicL*) were uncertain in the KH version, due to a large sequence gap near the *Foxd* locus, and because the *Tbx6-r.b* and *Zic-r.b* loci are located near scaffold ends. In the present HT assembly, two copies of *Foxd* were encoded on chromosome 8 as neighbors, and three copies of *Tbx6-r.b* were encoded within a 12-kb region of chromosome 11 (*Tbx6b–d* in supplementary fig. S5, Supplementary Material online). These copy numbers are the same as those predicted in the KH version. However, the current HT assembly revealed that another Tbx6-related gene, *Tbx6-r.a*, was encoded in the vicinity of the *Tbx6-r.b* copies (supplementary fig. S5, Supplementary Material online). The *Tbx6-r.a* locus was located ∼120 kb from the three copies of *Tbx6-r.b*, and seven genes are predicted in the intervening region. Although five copies of *Zic-r.b* were predicted previously (Yamada et al. 2003), the current HT assembly contained six copies of *Zic-r.b* in an 18-kb region of chromosome 6 (*Zic-r.b–g* in supplementary fig. S5, Supplementary Material online). Similarly, two copies of *Prdm1-related* gene, which encodes an important transcriptional repressor in early embryos, have been identified (Ikeda et al. 2013; Ikeda and Satou 2017). The current HT assembly contains another copy of *Prdm1-related* gene near the two copies previously identified on chromosome 12 (*Prdm1-r.a–c* in supplementary fig. S5, Supplementary Material online). There are four copies of type-A ephrin genes, which are thought to have been multiplied in the lineage leading to extant ascidians (Satou, Sasakura, et al. 2003). In the HT assembly, two additional type-A ephrin genes were identified in this gene cluster on chromosome 3 (*Efna.a–f* in supplementary fig. S5, Supplementary Material online). Thus, the current HT assembly contains fewer gaps and is continuous. As a result, the number and position of tandemly repeated copies can be determined unambiguously, showing that this long version of the genome more faithfully reproduces the genomic structure.

### Telomeres, Ribosomal RNA Genes, and *Trans*-Spliced Leader Donor RNA Genes

We found repetitive sequences at either or both ends of 10 chromosomes (table 3). Because the typical repeat sequence, CCCCTAA, was highly similar to telomeric repeats found in many organisms (CCCTAA) (Podlevsky et al. 2007), it is highly likely that these repeats constitute telomeres of these chromosomes. We found this repeat at both ends of chromosomes 3, 9, and 14, indicating that these chromosomes are almost completely assembled (supplementary table S3, Supplementary Material online). We also found this repeat at either end of chromosomes 4, 5, 6, 7, 10, 12, and 13. However, we did not find telomeric repeats at either end of chromosomes 1, 2, 8, and 11, although we found the repeats in three unassembled contigs.

**Table 3:** List of Chromosomes and Unassembled Contigs that Contain Telomeres, 18S/28S RNA Genes, and SL RNAs Genes

	Chromosomes/Unassembled Contigs
Chromosomes with telomeres in both ends	Chromosomes 3, 9, 14
Chromosomes with telomeres in either end	Chromosomes 4, 5, 6, 7, 10, 12, 13
Contigs containing 18S/28S RNA genes	UAContigs 2, 6, 7, 13, 17, 22, 28, 31, 32, 33, 34, 36, 38, 39, 41, 47, 49, 51, 53
Chromosomes/contigs containing SL RNA gene clusters	Chromosome 8, UAContigs 11, 12

The short arms of chromosomes 4, 5, and 6 contain 18S/28S ribosomal RNA genes (Shoguchi et al. 2005). These genes were not included in chromosomal sequences of the current assembly. Instead, they were found in 19 unassembled contigs (table 3). Probably because of their highly repetitive nature, these contigs were not successfully assembled.

Previous studies estimated that more than half of *Ciona* mRNA species have an SL at their 5′-ends (Vandenberghe et al. 2001; Satou et al. 2006; Matsumoto et al. 2010). This SL is added by *trans*-splicing, and its donor RNA is encoded by a multi-copy SL gene (Yeats et al. 2010). FISH analysis showed that these copies are located as a cluster in the short arm of chromosome 8 (Yeats et al. 2010). Indeed, we found 32 genes with high similarity to the previously identified SL gene in a 300-kb region near one end of chromosome 8. Two unassembled contigs contained two additional clusters, each of which contained 16 SL genes within a region of ∼90 kb (table 3). Although it is likely that these contigs encode sequences of the short arm of chromosome 8, we were not able to determine their precise locations, order, and orientations.

### Re-estimation of the Genome Size

The genome size of *C.intestinalis* (types unidentified) has been estimated between 140 and 190 Mb per haploid (Atkin and Ohno 1967; Laird 1971; Simmen et al. 1998). However, as described above, our assembly (∼123 Mb) contained almost the entire genome sequence, including highly repetitive segments. These observations suggested that the genome size had been overestimated. To test this hypothesis, we first re-estimated the genome size of the inbred strain. We adopted a method using k-mer profiles, implemented in GenomeScope (Vurture et al. 2017). We used two sets of Illumina sequencing reads obtained from two individuals of the 11th generation (F11a and F11b in supplementary fig. S2*B*, Supplementary Material online). According to GenomeScope, genome sizes of the two siblings were estimated at 116 and 114 Mb, respectively. To confirm that the inbred strain has the same genome size as individuals from natural populations, we prepared two specimens from a different geographic location than the source of the inbred line. The genome sizes of these specimens were estimated at 119 and 120 Mb. Therefore, it is likely that the actual genome size in natural populations is smaller than previously estimated. This means that the current assembly covers the vast majority of the genome of *C.intestinalis* type A (*C. robusta*).

### A 600-kb Inversion in the Inbred Strain

As mentioned above, Hi-C data mapping indicated an inversion in chromosome 4 (fig. 2*A* and *B*). This inversion was confirmed by aligning the new version and the KH/Hi-C-linked scaffold version of chromosome 4 (fig. 2*C*). These data consistently indicated that a region of ∼600 kb between nucleotide positions 1.9 and 2.5 M of chromosome 4 is inverted. Because the Hi-C data were obtained from tailbud embryos derived from eggs and sperm of wild-caught animals, it is possible that only the inbred animals have this large inversion. To test this possibility, we designed three PCR primers (fig. 2*D*), which were designated For, Test, and Rev. Because we did not retain DNA used for the genomic sequencing, we used a different animal of this inbred line (F8b in supplementary fig. S2*B*, Supplementary Material online). For comparison, we also used seven wild-caught animals. From the inbred line, we obtained a band with the set of primers For and Test, but not with the set of primers Rev and Test (fig. 2*E*). On the other hand, from wild-caught animals, we obtained a band with the set of primers Rev and Test, but not with the primers For and Test (fig. 2*E*). Therefore, this inversion occurred in the inbred line.

### A Comparison of Chromosomal Structures Between Two *Ciona* Species

Next, we compared these new chromosomes of *C.intestinalis* type A (*C. robusta*) with scaffolds of a species in the same genus, *C.savignyi*. Although a previous study performed a similar comparison and showed extensive intrachromosomal rearrangements between these two species (Hill et al. 2008), we expected that we could obtain much better resolution using the present assembly. For this purpose, we first mapped *C. savignyi* gene models predicted on scaffold sequences called reftigs (Small et al. 2007; Zerbino et al. 2018) to the KY gene model set.

We found that 122 reftigs contained 10 or more genes with putative orthologs among the KY gene models. Genes encoded by each of 106 reftigs (of the above 122) were found preferentially in one of the 14 HT-chromosomes (Fisher’s exact tests with Bonferroni correction <4.1e−4 = 0.05/122). Rank order positions of orthologous gene pairs in the 14 HT-chromosomes and the 106 reftigs of *C. savignyi* are shown in supplementary figure S6, Supplementary Material online. As previously shown (Hill et al. 2008), gene rearrangements were extensive within chromosomes, but not between pairs of the 14 chromosomes.

We noticed small inversions in our new mapping data. In chromosome 10, a region containing six orthologous gene pairs was clearly inverted between the two species (fig. 3*A* and *B*). Figure 3*C* shows a similar instance, in which a region that contained five orthologous gene pairs was inverted. Additional examples are shown in supplementary figure S7, Supplementary Material online. We also found a gene arrangement that can be explained by two serial inversions (fig. 3*D*), although one translocation could also explain the phenomenon. These examples indicate that inversions have occurred frequently and have contributed to intrachromosomal gene rearrangements in *Ciona* species.

## Discussion

### The Genome of *C. intestinalis* Type A (*C. robusta*) Is Smaller than Previously Estimated

We found telomeres on both ends of chromosomes of 3, 9, and 14. This indicates that these chromosomes are assembled almost completely from one end to the other. Their lengths were 11.2, 8.32, and 6.29 Mb, respectively, smaller than our previous estimates using cytogenetic data (13.0, 10.7, and 7.92 Mb, respectively) (Shoguchi et al. 2006). Therefore, the lengths of these chromosomes in the present assembly are 78–86% (mean = 81%) of the previously estimated sizes. Because this previous estimate was based on an assumption that the genome size was 162 Mb (Simmen et al. 1998), ∼132 Mb (162 Mb× 81%) is a rough estimate for the actual genome size of *C. intestinalis* type A (*C. robusta*).

Independently of the genome assembly, we used Illumina sequencing reads to estimate the genome size. Data from two siblings of the inbred strain and two wild-caught specimens gave estimates of 114–120 Mb per haploid. These values are close to the aforementioned estimate and to the total length of the current assembly (123 Mb).

In this way, our present data indicate that the actual genome size of *C.intestinalis* type A (*C. robusta*) is smaller than previous estimates (Atkin and Ohno 1967; Laird 1971; Simmen et al. 1998). However, because short arms of chromosomes 4, 5, and 6, which encode 18S/28S rDNA genes, show size polymorphisms (Shoguchi et al. 2005, 2006), the actual genome size may vary among individuals. Sequencing of genomes of seven larvacean species indicates that transposable elements contribute to interspecies variation in genome size (Naville et al. 2019). It is possible that repetitive sequences similarly contribute to intraspecies variation in genome size in ascidians. It is also possible that these earlier studies used animals different from *C.intestinalis* type A (*C. robusta*), and that the genome size of these animals is indeed larger than that of *C.intestinalis* type A (*C. robusta*).

### Quality of the Assembly

In the present assembly version, over 95% of nucleotides are included in chromosomal sequences, which are accessible through the DDBJ/EMBL/Genbank database and also through the Ghost database (Satou et al. 2005) (http://ghost.zool.kyoto-u.ac.jp/default_ht.html; last accessed October 24, 2019). In addition, 13,801 (98%) of 14,072 predicted genes are included in the chromosomes. Consistently, among 318 genes that encode transcription factors or signaling ligands, 317 are included in the chromosomes. Thus, most protein coding genes are included in chromosomal sequences of the current assembly. Unassembled contigs, in which the remaining 2% of genes are encoded, may not have been assembled into chromosomes due to technical problems, although we cannot rule out the possibility that some of these contigs constitute minichromosomes. Meanwhile, many genes encoding 18S/28S ribosomal RNAs and SL RNAs were found in unassembled contigs. rRNA genes are encoded in the short arms of chromosomes 4, 5, and 6, the total length of which is estimated at over 13 Mb (Shoguchi et al. 2006). The genome contains ∼670 copies of the genes for SL RNAs, most of which are encoded in the short arm of chromosome 8 (Yeats et al. 2010). Even with the long sequencing reads obtained with a PacBio RSII sequencer, such long repetitive sequences were difficult to reconstruct. 

In the present study, we constructed the assembly using PacBio RSII sequencing, Illumina sequencing, Hi-C, and FISH data. Such a combinatorial method worked efficiently, because long contigs obtained from long sequencing reads were greatly improved with Hi-C and FISH data. Specifically, the N50 value increased from 3.7 to 8.3 Mb. In the present study, Hi-C data were used to build reference scaffolds from an earlier version of the assembly, but not directly for connecting contigs. The resulting scaffolds were helpful for screening partially heterozygous regions. This method will be applicable for improving genome assemblies of other organisms.

*Ciona* has one of the simplest and most compact chordate genomes, which makes it a useful model system for analyzing higher-order genome structure. This genome may enable us to analyze global genomic regulatory landscapes more easily.

Copy numbers of multicopy genes that perform essential functions in embryonic development have been determined in the inbred strain. In the previous version of the genome assembly, many multicopy genes were located near sequencing gaps and scaffold ends, which prevented determination of exact copy numbers. We show here that copy numbers of the *Zic-r.b*, *Efna*, and *Prdm1-r* genes differ from those predicted in the previous version, although we do not know why these key genes are multicopy genes.

### An Inversion in the Inbred Line and Inversions between Two *Ciona* Species

We found a large inversion in the genome of the inbred line. Because we did not retain the DNA of the F0 animal, we are unable to determine whether the F0 animal had this inversion or whether it occurred during inbreeding. In the former scenario, this inversion may have occurred in a natural population. Although we cannot completely rule out this possibility, the latter scenario seems more likely because seven wild-caught individuals did not contain this inversion. If the latter scenario is the case, it may suggest that inversions occur frequently. Although such inversions may become fixed by genetic drift in natural populations if they are neutral, they will be fixed much more frequently in inbred lines established by self-crossing.

A previous study suggested that extensive intrachromosomal rearrangements have occurred after the split of the two *Ciona* species (Hill et al. 2008). This observation is best explained by the occurrence of frequent inversions, but not by frequent translocations, because inversions are intrachromosomal events, but translocations can occur both within and between chromosomes. We found several inversions in the genomes of the two *Ciona* species. This provides evidence for the contribution of inversions to gene rearrangements in *Ciona* chromosomes.

Chromosomal inversions have been implicated in speciation and local adaptation (Kirkpatrick 2010; Wellenreuther and Bernatchez 2018). In *Ciona* species, inversions may have contributed similarly to speciation and environmental adaptation. Inversions may also have shuffled genes within chromosomes both during and after speciation in the genus *Ciona*.

## Supplementary Material

Supplementary data are available at *Genome Biology and Evolution* online.

## Supplementary Material

evz228_Supplementary_Data

## Notes

**Data deposition:** This project has been deposited at the DDBJ BioProject database under the accessions PRJDB8078 (whole genome) and PRJDB8620 (Hi-C data). Accession numbers for genome sequence data are BJTB01000001–BJTB01000067; accession numbers for raw sequencing data are DRA008508 (genomic sequencing data for two wild-caught animals) and DRA008742 (Hi-C data).

## References

[evz228-B1] Abitua PB, et al 2015 The pre-vertebrate origins of neurogenic placodes. Nature 524(7566):462–465. 26258298 10.1038/nature14657 PMC5008972

[evz228-B2] Abitua PB, Wagner E, Navarrete IA, Levine M. 2012 Identification of a rudimentary neural crest in a non-vertebrate chordate. Nature 492(7427):104–107. 23135395 10.1038/nature11589 PMC4257486

[evz228-B3] Altschul SF, Gish W, Miller W, Myers EW, Lipman DJ. 1990 Basic local alignment search tool. J Mol Biol 215(3):403–410. 2231712 10.1016/S0022-2836(05)80360-2

[evz228-B4] Atkin NB, Ohno S. 1967 DNA values of four primitive chordates. Chromosoma 23(1):10–13.

[evz228-B5] Bouchemousse S, Liautard-Haag C, Bierne N, Viard F. 2016 Distinguishing contemporary hybridization from past introgression with postgenomic ancestry-informative SNPs in strongly differentiated *Ciona* species. Mol Ecol. 25(21):5527–5542. 27662427 10.1111/mec.13854

[evz228-B6] Brunetti R, et al 2015 Morphological evidence that the molecularly determined *Ciona intestinalis* type A and type B are different species: *Ciona robusta* and *Ciona intestinalis*. J Zool Syst Evol Res. 53(3):186–193.

[evz228-B7] Cao C, et al 2019 Comprehensive single-cell transcriptome lineages of a proto-vertebrate. Nature 571(7765):349–354. 31292549 10.1038/s41586-019-1385-y PMC6978789

[evz228-B8] Caputi L, et al 2007 Cryptic speciation in a model invertebrate chordate. Proc Natl Acad Sci U S A. 104(22):9364–9369. 17517633 10.1073/pnas.0610158104 PMC1890500

[evz228-B9] Carver T, Harris SR, Berriman M, Parkhill J, McQuillan JA. 2012 Artemis: an integrated platform for visualization and analysis of high-throughput sequence-based experimental data. Bioinformatics 28(4):464–469. 22199388 10.1093/bioinformatics/btr703 PMC3278759

[evz228-B10] Clark AG, et al 2007 Evolution of genes and genomes on the *Drosophila* phylogeny. Nature 450(7167):203–218. 17994087 10.1038/nature06341

[evz228-B11] Conklin EG. 1905 Mosaic development in ascidian eggs. J Exp Zool. 2(2):145–223.

[evz228-B12] Conklin EG. 1905 Organ-forming substances in the eggs of ascidians. Biol Bull. 8(4):205–230.

[evz228-B13] Dehal P, et al 2002 The draft genome of *Ciona intestinalis*: insights into chordate and vertebrate origins. Science 298(5601):2157–2167. 12481130 10.1126/science.1080049

[evz228-B14] Delsuc F, Brinkmann H, Chourrout D, Philippe H. 2006 Tunicates and not cephalochordates are the closest living relatives of vertebrates. Nature 439(7079):965–968. 16495997 10.1038/nature04336

[evz228-B15] Dudchenko O, et al 2017 De novo assembly of the *Aedes aegypti* genome using Hi-C yields chromosome-length scaffolds. Science 356(6333):92–95. 28336562 10.1126/science.aal3327 PMC5635820

[evz228-B16] Durand NC, et al 2016 Juicebox provides a visualization system for Hi-C contact maps with unlimited zoom. Cell Syst. 3(1):99–101. 27467250 10.1016/j.cels.2015.07.012 PMC5596920

[evz228-B17] Guindon S, Gascuel O. 2003 A simple, fast, and accurate algorithm to estimate large phylogenies by maximum likelihood. Syst Biol. 52(5):696–704. 14530136 10.1080/10635150390235520

[evz228-B18] Harada Y, et al 2008 Mechanism of self-sterility in a hermaphroditic chordate. Science. 320(5875):548–550. 18356489 10.1126/science.1152488

[evz228-B19] Hill MM, et al 2008 The *C. savignyi* genetic map and its integration with the reference sequence facilitates insights into chordate genome evolution. Genome Res. 18(8):1369–1379. 18519652 10.1101/gr.078576.108 PMC2493423

[evz228-B20] Hillier LW, et al 2007 Comparison of *C. elegans* and C. briggsae genome sequences reveals extensive conservation of chromosome organization and synteny. PLoS Biol. 5(7):e167–1616. 17608563 10.1371/journal.pbio.0050167 PMC1914384

[evz228-B21] Hino K, Satou Y, Yagi K, Satoh N. 2003 A genomewide survey of developmentally relevant genes in *Ciona intestinalis*. VI. Genes for Wnt, TGFbeta, Hedgehog and JAK/STAT signaling pathways. Dev Genes Evol. 213(5-6):264–272. 12739142 10.1007/s00427-003-0318-8

[evz228-B22] Horie R, et al 2018 Shared evolutionary origin of vertebrate neural crest and cranial placodes. Nature 560(7717):228–232. 30069052 10.1038/s41586-018-0385-7 PMC6390964

[evz228-B23] Ikeda T, Matsuoka T, Satou Y. 2013 A time delay gene circuit is required for palp formation in the ascidian embryo. Development 140(23):4703–4708. 24255097 10.1242/dev.100339

[evz228-B24] Ikeda T, Satou Y. 2017 Differential temporal control of *Foxa.a* and *Zic-r.b* specifies brain versus notochord fate in the ascidian embryo. Development 144(1):38–43. 27888196 10.1242/dev.142174

[evz228-B25] Imai KS, Hino K, Yagi K, Satoh N, Satou Y. 2004 Gene expression profiles of transcription factors and signaling molecules in the ascidian embryo: towards a comprehensive understanding of gene networks. Development 131(16):4047–4058. 15269171 10.1242/dev.01270

[evz228-B26] Imai KS, Levine M, Satoh N, Satou Y. 2006 Regulatory blueprint for a chordate embryo. Science 312(5777):1183–1187. 16728634 10.1126/science.1123404

[evz228-B27] Jeffery WR, et al 2008 Trunk lateral cells are neural crest-like cells in the ascidian *Ciona intestinalis*: insights into the ancestry and evolution of the neural crest. Dev Biol. 324(1):152–160. 18801357 10.1016/j.ydbio.2008.08.022

[evz228-B28] Kasahara M, et al 2007 The medaka draft genome and insights into vertebrate genome evolution. Nature 447(7145):714–719. 17554307 10.1038/nature05846

[evz228-B29] Kent WJ. 2002 BLAT—the BLAST-like alignment tool. Genome Res. 12(4):656–664. 11932250 10.1101/gr.229202 PMC187518

[evz228-B30] Kirkpatrick M. 2010 How and why chromosome inversions evolve. PLoS Biol. 8(9):e1000501. 20927412 10.1371/journal.pbio.1000501 PMC2946949

[evz228-B31] Kobayashi M, et al 2002 Construction of BAC libraries derived from the ascidian *Ciona intestinalis*. Genes Genet Syst. 77(4):283–285. 12419901 10.1266/ggs.77.283

[evz228-B32] Kourakis MJ, et al 2019 Parallel visual circuitry in a basal chordate. Elife 8: e44753. 10.7554/eLife.44753 PMC6499539 30998184

[evz228-B33] Kurtz S, et al 2004 Versatile and open software for comparing large genomes. Genome Biol. 5(2):R12. 14759262 10.1186/gb-2004-5-2-r12 PMC395750

[evz228-B34] Laird CD. 1971 Chromatid structure: relationship between DNA content and nucleotide sequence diversity. Chromosoma 32(4):378–406. 4995642 10.1007/BF00285251

[evz228-B35] Langmead B, Wilks C, Antonescu V, Charles R. 2019 Scaling read aligners to hundreds of threads on general-purpose processors. Bioinformatics 35(3):421–432. 30020410 10.1093/bioinformatics/bty648 PMC6361242

[evz228-B36] Lemaire P. 2011 Evolutionary crossroads in developmental biology: the tunicates. Development 138(11):2143–2152. 21558365 10.1242/dev.048975

[evz228-B37] Madgwick A, et al 2019 Evolution of embryonic cis-regulatory landscapes between divergent *Phallusia* and *Ciona ascidians*. Dev Biol. 448(2):71–87. 30661644 10.1016/j.ydbio.2019.01.003

[evz228-B38] Manni L, et al 2004 Neurogenic and non-neurogenic placodes in ascidians. J Exp Zool B Mol Dev Evol. 302(5):483–504. 15384166 10.1002/jez.b.21013

[evz228-B39] Marcais G, Kingsford C. 2011 A fast, lock-free approach for efficient parallel counting of occurrences of k-mers. Bioinformatics 27:764–770. 21217122 10.1093/bioinformatics/btr011 PMC3051319

[evz228-B40] Matsubara S, Shiraishi A, Osugi T, Kawada T, Satake H. 2019 The regulation of oocyte maturation and ovulation in the closest sister group of vertebrates. Elife 8. 10.7554/eLife.49062 PMC6786877 31573508

[evz228-B41] Matsumoto J, et al 2010 High-throughput sequence analysis of *Ciona intestinalis* SL trans-spliced mRNAs: alternative expression modes and gene function correlates. Genome Res. 20(5):636–645. 20212022 10.1101/gr.100271.109 PMC2860165

[evz228-B42] Mazet F, et al 2005 Molecular evidence from *Ciona intestinalis* for the evolutionary origin of vertebrate sensory placodes. Dev Biol. 282(2):494–508. 15950613 10.1016/j.ydbio.2005.02.021

[evz228-B43] Mizutani N, Okochi Y, Okamura Y. 2019 Distinct functional properties of two electrogenic isoforms of the SLC34 Na-Pi cotransporter. Physiol Rep. 7(14):e14156. 31342668 10.14814/phy2.14156 PMC6656865

[evz228-B44] Naville M, et al 2019 Massive changes of genome size driven by expansions of non-autonomous transposable elements. Curr Biol. 29(7):1161. 30880010 10.1016/j.cub.2019.01.080

[evz228-B45] Nydam ML, Harrison RG. 2007 Genealogical relationships within and among shallow-water *Ciona* species (Ascidiacea). Mar Biol. 151(5):1839–1847.

[evz228-B46] Nydam ML, Harrison RG. 2010 Polymorphism and divergence within the ascidian genus *Ciona*. Mol Phylogenet Evol. 56(2):718–726. 20403444 10.1016/j.ympev.2010.03.042

[evz228-B47] Nydam ML, Harrison RG. 2011a Introgression despite substantial divergence in a broadcast spawning marine invertebrate. Evolution 65(2):429–442. 21044056 10.1111/j.1558-5646.2010.01153.x

[evz228-B48] Nydam ML, Harrison RG. 2011b Reproductive protein evolution in two cryptic species of marine chordate. BMC Evol Biol. 11(1). 10.1186/1471-2148-11-18 PMC3036616 21247489

[evz228-B49] O’Leary NA, et al 2016 Reference sequence (RefSeq) database at NCBI: current status, taxonomic expansion, and functional annotation. Nucleic Acids Res. 44(D1):D733–D745. 26553804 10.1093/nar/gkv1189 PMC4702849

[evz228-B50] Oonuma K, Kusakabe TG. 2019 Spatio-temporal regulation of *Rx* and mitotic patterns shape the eye-cup of the photoreceptor cells in *Ciona*. Dev Biol. 445(2):245–255. 30502325 10.1016/j.ydbio.2018.11.011

[evz228-B51] Podlevsky JD, Bley CJ, Omana RV, Qi X, Chen JJ. 2007 The telomerase database. Nucleic Acids Res. 36(Database):D339–343. 18073191 10.1093/nar/gkm700 PMC2238860

[evz228-B52] Putnam NH, et al 2008 The amphioxus genome and the evolution of the chordate karyotype. Nature 453(7198):1064–1071. 18563158 10.1038/nature06967

[evz228-B53] Rao SSP, et al 2014 A 3D map of the human genome at kilobase resolution reveals principles of chromatin looping. Cell 159(7):1665–1680. 25497547 10.1016/j.cell.2014.11.021 PMC5635824

[evz228-B54] Roux C, Tsagkogeorga G, Bierne N, Galtier N. 2013 Crossing the species barrier: genomic hotspots of introgression between two highly divergent *Ciona intestinalis* species. Mol Biol Evol. 30(7):1574–1587. 23564941 10.1093/molbev/mst066

[evz228-B55] Sato A, Satoh N, Bishop JDD. 2012 Field identification of ‘types’ A and B of the ascidian *Ciona intestinalis* in a region of sympatry. Mar Biol. 159(7):1611–1619.

[evz228-B56] Sato A, Shimeld SM, Bishop JDD. 2014 Symmetrical reproductive compatibility of two species in the *Ciona intestinalis* (Ascidiacea) species complex, a model for marine genomics and developmental biology. Zool Sci. 31(6):369–374. 24882097 10.2108/zs130249

[evz228-B57] Satoh N. 2003 The ascidian tadpole larva: comparative molecular development and genomics. Nat Rev Genet. 4(4):285–295. 12671659 10.1038/nrg1042

[evz228-B58] Satoh N. 2013 Developmental Genomics of Ascidians. Hoboken, New Jersey: Wiley‐Blackwell.

[evz228-B59] Satoh N. 2016 Chordate Origins and Evolution: the Molecular Evolutionary Road to Vertebrates. London: Elsevier.

[evz228-B60] Satoh T, et al 2018 piRNA-like small RNAs are responsible for the maternal-specific knockdown in the ascidian *Ciona intestinalis* type A. Sci Rep. 8(1):5869. 29651003 10.1038/s41598-018-24319-w PMC5897368

[evz228-B61] Satou Y, Hamaguchi M, Takeuchi K, Hastings KEM, Satoh N. 2006 Genomic overview of mRNA 5′-leader trans-splicing in the ascidian *Ciona intestinalis*. Nucleic Acids Res. 34(11):3378–3388. 16822859 10.1093/nar/gkl418 PMC1488885

[evz228-B62] Satou Y, et al 2015 Sustained heterozygosity across a self-incompatibility locus in an inbred ascidian. Mol Biol Evol. 32(1):81–90. 25234703 10.1093/molbev/msu268

[evz228-B63] Satou Y, Imai KS et al 2003 A genomewide survey of developmentally relevant genes in *Ciona intestinalis*. I. Genes for bHLH transcription factors. Dev Genes Evol. 213(5–6):213–221. 12736824 10.1007/s00427-003-0319-7

[evz228-B64] Satou Y, Kawashima T, Shoguchi E, Nakayama A, Satoh N. 2005 An integrated database of the ascidian, *Ciona intestinalis*: towards functional genomics. Zool Sci. 22(8):837–843. 16141696 10.2108/zsj.22.837

[evz228-B65] Satou Y, Mineta K, et al 2008 Improved genome assembly and evidence-based global gene model set for the chordate *Ciona intestinalis*: new insight into intron and operon populations. Genome Biol. 9(10):R152. 18854010 10.1186/gb-2008-9-10-r152 PMC2760879

[evz228-B66] Satou Y, Sasakura Y, et al 2003 A genomewide survey of developmentally relevant genes in *Ciona intestinalis*. V. Genes for receptor tyrosine kinase pathway and Notch signaling pathway. Dev Genes Evol. 213(5–6):254–263. 12739141 10.1007/s00427-003-0317-9

[evz228-B67] Satou Y, Satoh N. 2005 Cataloging transcription factor and major signaling molecule genes for functional genomic studies in *Ciona intestinalis*. Dev Genes Evol. 215(11):580–596. 16252120 10.1007/s00427-005-0016-9

[evz228-B68] Satou Y, Shin-i T, Kohara Y, Satoh N, Chiba S. 2012 A genomic overview of short genetic variations in a basal chordate, *Ciona intestinalis*. BMC Genomics. 13(1):208. 22646724 10.1186/1471-2164-13-208 PMC3424144

[evz228-B69] Satou Y, Wada S, Sasakura Y, Satoh N. 2008 Regulatory genes in the ancestral chordate genomes. Dev Genes Evol. 218(11–12):715–721. 18773221 10.1007/s00427-008-0219-y

[evz228-B70] Shoguchi E, et al 2004 Fluorescent in situ hybridization to ascidian chromosomes. Zool Sci. 21(2):153–157. 14993826 10.2108/zsj.21.153

[evz228-B71] Shoguchi E, Kawashima T, Nishida-Umehara C, Matsuda Y, Satoh N. 2005 Molecular cytogenetic characterization of *Ciona intestinalis* chromosomes. Zool Sci. 22(5):511–516. 15930823 10.2108/zsj.22.511

[evz228-B72] Shoguchi E, et al 2006 Chromosomal mapping of 170 BAC clones in the ascidian *Ciona intestinalis*. Genome Res. 16(2):297–303. 16354750 10.1101/gr.4156606 PMC1361726

[evz228-B73] Sievers F, et al 2011 Fast, scalable generation of high-quality protein multiple sequence alignments using Clustal Omega. Mol Syst Biol. 7. 10.1038/msb.2011.75 PMC3261699 21988835

[evz228-B74] Simao FA, Waterhouse RM, Ioannidis P, Kriventseva EV, Zdobnov EM. 2015 BUSCO: assessing genome assembly and annotation completeness with single-copy orthologs. Bioinformatics 31:3210–3212. 26059717 10.1093/bioinformatics/btv351

[evz228-B75] Simmen MW, Leitgeb S, Clark VH, Jones SJ, Bird A. 1998 Gene number in an invertebrate chordate, *Ciona intestinalis*. Proc Natl Acad Sci U S A. 95(8):4437–4440. 9539755 10.1073/pnas.95.8.4437 PMC22507

[evz228-B76] Small KS, Brudno M, Hill MM, Sidow A. 2007 A haplome alignment and reference sequence of the highly polymorphic *Ciona savignyi* genome. Genome Biol. 8(3):R41. 17374142 10.1186/gb-2007-8-3-r41 PMC1868934

[evz228-B77] Stanke M, Diekhans M, Baertsch R, Haussler D. 2008 Using native and syntenically mapped cDNA alignments to improve de novo gene finding. Bioinformatics. 24(5):637–644. 18218656 10.1093/bioinformatics/btn013

[evz228-B78] Stolfi A, Ryan K, Meinertzhagen IA, Christiaen L. 2015 Migratory neuronal progenitors arise from the neural plate borders in tunicates. Nature 527(7578):371–374. 26524532 10.1038/nature15758 PMC4654654

[evz228-B79] Suzuki MM, Kerr ARW, De Sousa D, Bird A. 2007 CpG methylation is targeted to transcription units in an invertebrate genome. Genome Res. 17(5):625–631. 17420183 10.1101/gr.6163007 PMC1855171

[evz228-B80] Tamazian G, et al 2016 Chromosomer: a reference-based genome arrangement tool for producing draft chromosome sequences. GigaScience 5(1):38. 27549770 10.1186/s13742-016-0141-6 PMC4994284

[evz228-B81] Tresser J, et al 2010 doublesex/mab3 related-1 (dmrt1) is essential for development of anterior neural plate derivatives in *Ciona*. Development 137(13):2197–2203. 20530547 10.1242/dev.045302 PMC2882137

[evz228-B82] Vandenberghe AE, Meedel TH, Hastings KE. 2001 mRNA 5′-leader trans-splicing in the chordates. Genes Dev. 15(3):294–303. 11159910 10.1101/gad.865401 PMC312621

[evz228-B83] Vurture GW, et al 2017 GenomeScope: fast reference-free genome profiling from short reads. Bioinformatics 33(14):2202–2204. 28369201 10.1093/bioinformatics/btx153 PMC5870704

[evz228-B84] Wada S, et al 2003 A genomewide survey of developmentally relevant genes in *Ciona intestinalis*. II. Genes for homeobox transcription factors. Dev Genes Evol. 213(5–6):222–234. 12736825 10.1007/s00427-003-0321-0

[evz228-B85] Wagner E, Levine M. 2012 FGF signaling establishes the anterior border of the *Ciona* neural tube. Development 139(13):2351–2359. 22627287 10.1242/dev.078485 PMC6514301

[evz228-B86] Waki K, Imai KS, Satou Y. 2015 Genetic pathways for differentiation of the peripheral nervous system in ascidians. Nat Commun. 6:8719. 26515371 10.1038/ncomms9719 PMC4640076

[evz228-B87] Walker BJ, et al 2014 Pilon: an integrated tool for comprehensive microbial variant detection and genome assembly improvement. PLoS ONE 9(11):e112963. 25409509 10.1371/journal.pone.0112963 PMC4237348

[evz228-B88] Waterston RH, et al 2002 Initial sequencing and comparative analysis of the mouse genome. Nature 420(6915):520–562. 12466850 10.1038/nature01262

[evz228-B89] Wellenreuther M, Bernatchez L. 2018 Eco-evolutionary genomics of chromosomal inversions. Trends Ecol Evol (Amst). 33(6):427–440. 29731154 10.1016/j.tree.2018.04.002

[evz228-B90] Xiao CL, et al 2017 MECAT: fast mapping, error correction, and de novo assembly for single-molecule sequencing reads. Nat Methods 14(11):1072–1074. 28945707 10.1038/nmeth.4432

[evz228-B91] Yagi K, et al 2003 A genomewide survey of developmentally relevant genes in *Ciona intestinalis*. III. Genes for Fox, ETS, nuclear receptors and NFkappaB. Dev Genes Evol. 213(5–6):235–244. 12743820 10.1007/s00427-003-0322-z

[evz228-B92] Yamada L, Kobayashi K, Degnan B, Satoh N, Satou Y. 2003 A genomewide survey of developmentally relevant genes in *Ciona intestinalis*. IV. Genes for HMG transcriptional regulators, bZip and GATA/Gli/Zic/Snail. Dev Genes Evol. 213(5–6):245–253. 12743819 10.1007/s00427-003-0316-x

[evz228-B93] Yeats B, et al 2010 SL RNA genes of the Ascidian tunicates *Ciona intestinalis* and *Ciona savignyi*. Zool Sci. 27(2):171–180. 20141422 10.2108/zsj.27.171

[evz228-B94] Yokomori R, et al 2016 Genome-wide identification and characterization of transcription start sites and promoters in the tunicate *Ciona intestinalis*. Genome Res. 26(1):140–150. 26668163 10.1101/gr.184648.114 PMC4691747

[evz228-B95] Yoshida K, et al 2017 Hox-mediated endodermal identity patterns pharyngeal muscle formation in the chordate pharynx. Development 144(9):1629–1634. 28289133 10.1242/dev.144436

[evz228-B96] Zerbino DR, et al 2018 Ensembl 2018. Nucleic Acids Res. 46(D1):D754–D761. 29155950 10.1093/nar/gkx1098 PMC5753206

