# Supplementary material for: A Nearly Complete Genome of Ciona intestinalis Type A (C. robusta) Reveals the Contribution of Inversion to Chromosomal Evolution in the Genus Ciona
Source: Genome Biol Evol. 2019 Oct 22;11(11):3144–57. doi: 10.1093/gbe/evz228 (PMC6836712; doi:10.1093/gbe/evz228)
Supplement: evz228_Supplementary_Data [file evz228_supplementary_data.pdf]

Supplementary figure S1. Molecular phylogenetic analyses to confirm that the animal we used is *Ciona intestinalis* type A (*C. robusta*). Molecular phylogenetic analyses to confirm that the animal we used is *Ciona intestinalis* type A (*C. robusta*). The sequences retrieved from the HT assembly is shown by magenta. Labels for other sequences indicate their accession numbers for the DDBJ/EMBL/Genbank database. Sequences for type A and type B animals are shown in blue and green letters, respectively. These classifications are based on a previous study (Nydam and Harrison, 2011). Note that HQ219485, which has been annotated as type A, is likely to be a type B locus. The number noted at a given branch indicates the percentage of times that a node was supported in 100 bootstrap pseudoreplications. These numbers are provided only when the values are 100%. The alignments before removing gaps are shown in the subsequent pages.

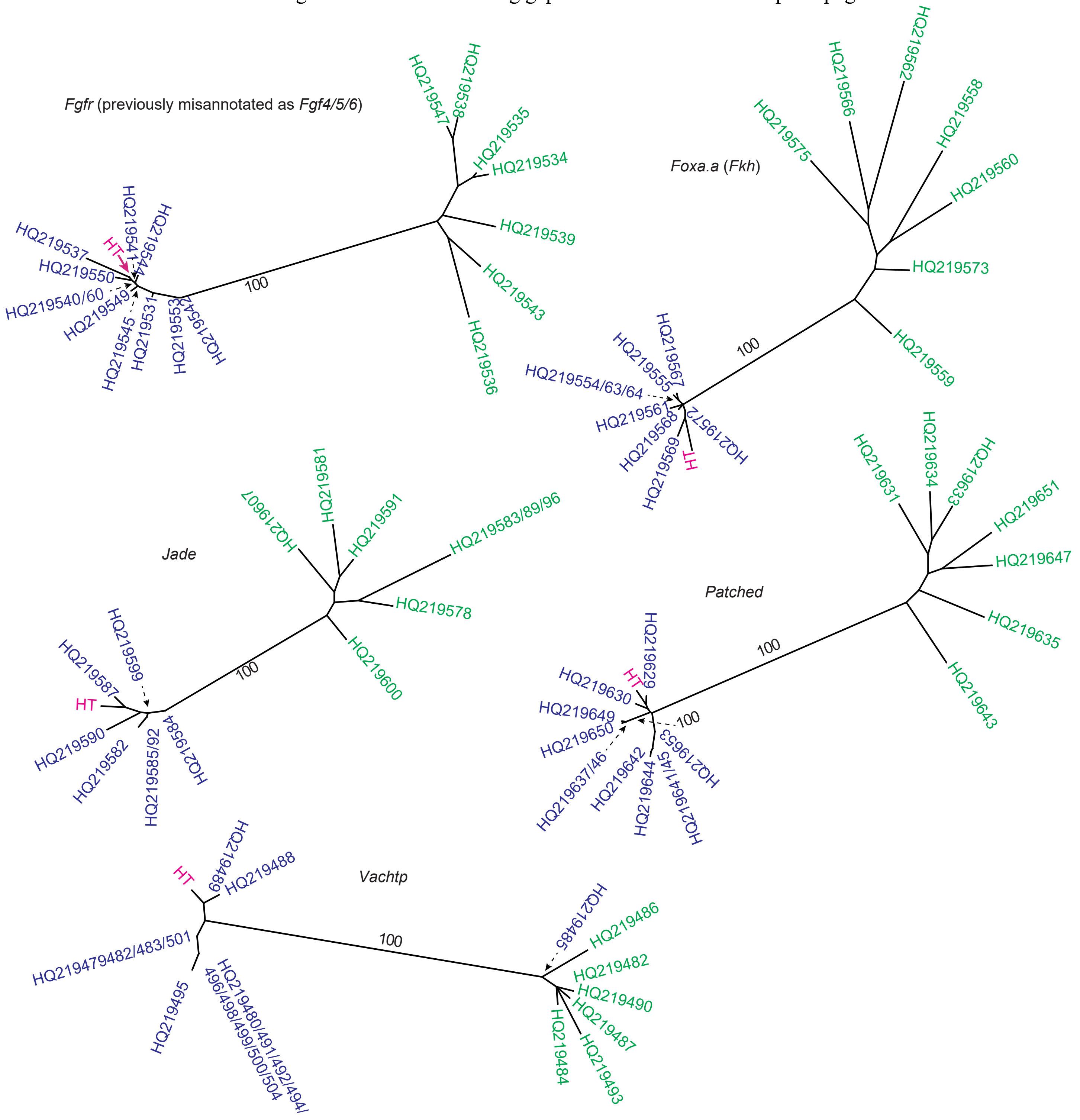

Fgf4/5/6 alignment

```
HQ219541_A GACAATTT-CCTCGGGTAGTTTT-ACCCGCAATGTG-TTTTAACGACCGTTCTGCTGCTAA-TCTATTTTCTTTGTTGTT
HQ219537_A GACAATTTTCCTCGAGTAGTTTT-ACCCGCAATGTGTTTTTAACGACCGTTCTGCTGCTAATTCTATTTTCTTTGTTGTT
HQ219553_A GACAATTTTCCTCGGGTAGTTTT-ACCCGCAATGTGTTTTTAACGACCGTTCTGCTGCTAATTCTATTTTCTTTGTTGTT
HQ219542_A GACAATTTTCCTCGGGTAGTTTT-ACCCGCAATGTGTTTTTAACGACCGTTCTGCTGCTAATTCTATTTTCTTTGTTGTT
HQ219531_A GACAATTTTCCTCGGGTAGTTTT-ACCCGCAATGTGTTTTTAACGACCGTTCTGCTGCTAATTCTATTTTCTTTGTTGTT
HQ219550_A GACAATTTTCCTCGGGTAGTTTT-ACCCGCAATGTGTTTTTAACGACCGTTCTGCTGCTAATTCTATTTTCTTTGTTGTT
HT         GACAATTTTCCTCGAGTAGTTTT-ACCCGCAATGTGTTTTTAACGACCGTTCTGCTGCTAATTCTATTTTCTTTGTTGTT
HQ219544_A GACAATTTTCCTCGGGTAGTTTT-ACCCGCAATGTGTTTTTAACGACCGTTCTGCTGCTAATTCTATTTTCTTTGTTGTT
HQ219549_A GACAATTTTCCTCGGGTAGTTTT-ACCCGCAATGTGTTTTTAACGACCGTTCTGCTGCTAATTCTATTTTCTTTGTTGTT
HQ219546_A GACAATTTTCCTCGGGTAGTTTT-ACCCGCAATGTGTTTTTAACGACCGTTCTGCTGCTAATTCTATTTTCTTTGTTGTT
HQ219540_A GACAATTTTCCTCGGGTAGTTTT-ACCCGCAATGTGTTTTTAACGACCGTTCTGCTGCTAATTCTATTTTCTTTGTTGTT
HQ219545_A GACAATTTTCCTCGGGTAGTTTT-ACCCGCAATGTGTTTTTAACGACCGTTCTGCTGCTAATTCTATTTTCTTTGTTGTT
HQ219543_B GACAATTTTCCTCGGGTAGTTTTTACCCGCGGCGTGATTTTAACGACCGTTTTGCTGCTAACTCCATTTTCTTTGTTGTT
HQ219539_B GACAATTTTCCTCGGGTAGTTTTTTCCCGCAGC-TGTTTTTAACGACCGTTTTGCTGCTAACTCCATTTTCTTTGTTGTT
HQ219536_B GACAATTTTCCTCGAGTAGTTTTTACCCGCGGCATGATTTTAACGACCGTTTTGCTGCTAACTCCATTTTCTTCGTTGTT
HQ219547_B GACAATTTTCCTCGGGTAGTTTTTACCCGCGGCGTGATTTTAACGACCGTTTTGCTGCTAACTCCATTTTCTTCGTTGTT
HQ219538_B GACAATTTTCCTCGGGTAGTTTTTACCCGCGGCGTGATTTTAACGACCGTTTTGCTGCTAACTCCATTTTCTTTGTTGTT
HQ219534_B GACAATTTTCCTCGAGTAGTTTTTACCCGCGGCGTGATTTTAACGACCGTTTTGCTGCTAACTCCATTTTCTTTGTTGTT
HQ219535_B GACAATTTTCCTCGGGTAGTTTTTACCCGCGGG-TG-TTTTAACGACCGTTTTGCTGCTAACTCCATTTTCTTTGTTGTT

HQ219541_A TTAATTCTCGTATTCGAAAAGATAAATAAAATTCACGGTTTTCA---ATACTGGATGCTTATA-AA-ACATGAAAATATA
HQ219537_A TTAATTCTCGTATTCGAAAAGATAAATAAAATTCACGGTTTTCA---ATACTGGATGCTTATACAACACATGAAAATATA
HQ219553_A TTAATTCTCGTATTCGAAAAGATAAATAAAATTCACGGTTTTCA---ATACTGGATGCTTATACAACACATGAAAATATA
HQ219542_A TTAATTCTCGTATTCGAAAAGATAAATAAAATTCACGGTTTTCA---ATACTGGATGCTTATATAATACATGAAAATATA
HQ219531_A TTAATTCTCGTATTCGAAAAGATAAATAAAATTCACGGTTTTCA---ATACTGGATGCTTATACAACACATGAAAATATA
HQ219550_A TTAATTCTCGTATTCGAAAAGATAAATAAAATTCACGGTTTTCA---ATACTGGATGCTTATACAACACATGAAAATATA
HT         TTAATTCTCGTATTCGAAAAGATAAATAAAATTCACGGTTTTCA---ATACTGGATGCTTATACAACACATGAAAATATA
HQ219544_A TTAATTCTCGTATTCGAAAAGATAAATAAAATTCACGGTTTTCA---ATACTGGATGCTTATACAACACATGAAAATATA
HQ219549_A TTAATTCTCGTATTCGAAAAGATAAATAAAATTCACGGTTTTCA---ATACTGGATGCTTATACAACACATGAAAATATA
HQ219546_A TTAATTCTCGTATTCGAAAAGATAAATAAAATTCACGGTTTTCA---ATACTGGATGCTTATACAACACATGAAAATATA
HQ219540_A TTAATTCTCGTATTCGAAAAGATAAATAAAATTCACGGTTTTCA---ATACTGGATGCTTATACAACACATGAAAATATA
HQ219545_A TTAATTCTCGTATTCGAAAAGATAAATAAAATTCACGGTTTTCA---ATACTGGATGCTTATACAACACATGAAAATATA
HQ219543_B TTAATTCTCGTACTCGAAAAGATA-ATAAATTTCACGATTATCAGTAATATTGGATGCTTATGTAATGCATGAAAATATA
HQ219539_B TTAATTCTCGTATTCGAAAAGAC---AAAATTTCACGCTTATCAGTAATATTGGATGCTTATGTAATGCATGAAAATATA
HQ219536_B TTAATTCTCGTATTCGAAAAGATAAATAAATTTCACGGTTATCAGTAATATTGGATGCTTATGTAATGCATGAAAATATA
HQ219547_B TTAATTCTCGTATTCGAAAAGACAAATAAATTTCACGGTTATCAGTAATATTGGATGCTTATGTAATACATGAAAATATA
HQ219538_B TTAATTCTCGTATTCGAAAAGATAAATAAATTTCACGGTTATCAGTAATATAGGATGCTTATGTAATACATGAAAATATA
HQ219534_B TTAATTCTCGTATTCGAAAAGATAAATAAATTTCACGGTTATCAGTAATATTGGATGCTTATGTAATACATGAAAATATA
HQ219535_B TTAATTCTCGTATTCGAAAAGATAAATAAATTTCACGGTTATCAGTAATATTGGATGCTTATGTAATACATGAAAATATA

HQ219541_A GCTGCGTTTGGTATTTGGTTATATATTATATAACAGACAACATTATAATACAGCTAT---------AGCTTGTTTAGCTA
HQ219537_A GCTGCGTTTGGTATTTGGTTATATATTATATAACAGACAACATTATAATACAGCTAT---------AGCTTGTTTAGCTA
HQ219553_A GCTGCGTTTGGTATTTGGTTATATATTATATAACAGACAACATTATAATACAGCTAT---------AGCTTGTTTAGCTA
HQ219542_A GCTGCGTTTGGTATTTGGTTATATATTATATAACAGACAACATTATAATACAGCTAT---------AGCTTGTTTAGCTA
HQ219531_A GCTGCGTTTGGTATTTGGTAATATATGATATAACAGACAACATTATAATACAGCTAT---------AGCTTGTTTAGCTA
HQ219550_A GCTGCGTTTGGTATTTGGTTATATATTATATAACAGACAACATTATAATACAGCTAT---------AGCTTGTTTAGCTA
HT         GCTGCGTTTGGTATTTGGTTATATATTATATAACAGACAACATTATAATACAGCTAT---------AGCTTGTTTAGCTA
HQ219544_A GCTGCGTTTGGTATTTGGTTATATATTATATAACAGACAACATTATAATACAGCTAT---------AGCTTGTTTAGCTA
HQ219549_A GCTGCGTTTGTAATTTGGTTATATATGATATAACAGACAACATTATAATACAGCTAT---------AGCTTGTTTAGCTA
HQ219546_A GCTGCGTTTGGTATTTGGTTATATATTATATAACAGACAACATTATAATACAGCTAT---------AGCTTGTTTAGCTA
HQ219540_A GCTGCGTTTGGTATTTGGTTATATATTATATAACAGACAACATTATAATACAGCTAT---------AGCTTGTTTAGCTA
HQ219545_A GCTGCGTTTGGTATTTGGTTATATATGATATAACAGACAACATTATAATACAGCTAT---------AGCTTGTTTAGCTA
HQ219543_B GCTGCGTTTGGTATTTGGTAATCTATTATGTAACAGACAATATTATAATACAGCTAT----AACATAACTTGTTTATCTC
HQ219539_B GCTGCGTTTGGTATTTGGTAATGTATTATGTAACAGACAATATTATGATACAGCTAT----AACATAACTTGTTTATCTC
HQ219536_B GCTGGGTTTGGTATTTGGTAATCTATTATGTAACAGACAATATTATAATACAGCTAT----AACATAACTTATTTATCTC
HQ219547_B GCTGGGTTTGGTATTTGGCAATCTATTATATAACAGACAATATTATAATACAGCTAT----AACATAACTTGTTTATCTC
HQ219538_B GCTGGGTTTGGTATTAGGAAATCTATTATGTAACAGACAATATTATAATACAGCTAT----AACATAACTTGTTTATCTC
HQ219534_B GCTGGGTTTGATATTTGGTAATATATTATGTAACAGACAATATTATAATACAGCTATATATAACATAACTTGTTTATCTC
HQ219535_B GCTGCGTTTGGTATTTGGTAATATATTATGTAACAGACAATATTATAATACAGCTAT----AGCATAACTTGTTTATCTC

HQ219541_A TTTCGTTACGGTAGCGAAGATTAATA---ATGGACACGAGCGAAACGACGGGATATAGCGACAAAACAACAGTCGTTAAA
HQ219537_A TTTCGTTACGGTAGCGAAGATTAATA---ATGGACACGAGCGAAACGACGGGATATAGCGACAAAACAACAGTCGTTAAA
HQ219553_A TTTCGTTACGGTAGCGAAGATTAATA---ATGGACACGAGCGAAACGACGGGATATAGCGACAAAACAACAGTCGTTAAA
HQ219542_A TTTCGTTACGGTAGCGAAGATTAATA---ATGGACACGAGCGAAACGACGGGATATAGCGACAAAACAACAGTCGTTAAA
HQ219531_A TTTCGTTACGGTAGCGAAGATTAATA---ATGGACACGAGCGAAACGACGGGATATAGCGACAAAACAACAGTCGTTAAA
HQ219550_A TTTCGTTACGGTAGCGAAGATTAATA---ATGGACACGAGCGAAACGACGGGATATAGCGACAAAACAACAGTCGTTAAA
HT         TTTCGTTACGGTAGCGAAGATTAATA---ATGGACACGAGCGAAACGACGGGATATAGCGACAAAACAACAGTCGTTAAA
HQ219544_A TTTCGTTACGGTAGCGAAGATTAATA---ATGGACACGAGCGAAACGACGGGATATAGCGACAAAACAACAGTCGTTAAA
HQ219549_A TTTCGTTACGGTAGCGAAGATTAATA---ATGGACACGAGCGAAACGACGGGATATAGCGACAAAACAACAGTCGTTAAA
HQ219546_A TTTCGTTACGGTAGCGAAGATTAATA---ATGGACACGAGCGAAACGACGGGATATAGCGACAAAACAACAGTCGTTAAA
HQ219540_A TTTCGTTACGGTAGCGAAGATTAATA---ATGGACACGAGCGAAACGACGGGATATAGCGACAAAACAACAGTCGTTAAA
HQ219545_A TTTCGTTACGGTAGCGAAGATTAATA---ATGGACACGAGCGAAACGACGGGATATAGCGACAAAACAACAGTCGTTAAA
HQ219543_B TTCGATTACGTTAGCGAAGATTAATAATAATGGACACGAACGAAACGACGGGATATAGCGACAAAACAACAGTCGTTAAA
HQ219539_B CTCGATTACGTTAGCGAAGATTAATAATAATGGACACGAACGAAACGACGGGATATAGCGACAAAACAACAGTCGTTAAA
HQ219536_B TTCGATTACGTTAGCGAAGATTAATA---ATGGACACGAACAAAACGACGAGATATAGCGACAAAACAACAGTCGTTAAA
HQ219547_B TTCGATTACGTTAGCGAAGATTAATAATGATGGACACAAACGAAACGACTGGATATAGCGACAAAACAACAGTCATTAAA
HQ219538_B TTCGATTACGTTAGCGAAGATTAATAATGATGGACACAAACGAAACGACTGGATATAGCGACAAAACAACAGTCATTAAA
HQ219534_B TTCGATTACGTTAGCGAAGATTAATA---ATGGACACAAACGAAACGACTGGATATAGCGACAAAACAACAGTCGTTAAA
HQ219535_B TTCGATTACGTTAGCGAAGATTAATA---ATGGACACGAACGAAACGACTGGATATAGCGACAAAACAACAGTCGTTAAA

HQ219541_A ACACGCTCACGGTTAAAAACGA--------ACATTACTGAACAATTAAAAAGAGTGGTTGCTACACATAGCAGACAAATA
HQ219537_A ACACGCTCACGGTTAAAAACGA--------ACATTACTGAATGATTAAAAAGAGTGGTTGCTACACCTAGCAGACAAATA
HQ219553_A ACACGCTCACGGTTAAAAACGA--------ACATTACTGAACAATTAAAAAGAGTGGTTGCTACACATAGCAGACAAATA
HQ219542_A ACACGCTCACGGTTAAAAACGA--------ACATTACTGAACAATTAAAAAGAGTGGTTGCTACACATAGCAGACAAATA
HQ219531_A ACACGCTCACGGTTAAAAACGA--------ACATTACTGAACAATTAAAAAGAGTGGTTGCTACACATAGCAGACAAATA
HQ219550_A ACACGCTCACGGTTAAAAACGA--------ACATTACTGAACAATTAAAAAGAGTGGTTGCTACACATAGCAGACAAATA
HT         ACACGCTCACGGTTAAAAACGA--------ACATTACTGAACAATTAAAAAGAGTGGTTGCTACACATAGCAGACAAATA
HQ219544_A ACACGCTCACGGTTAAAAACGA--------ACATTACTGAACAATTAAAAAGAGTGGTTGCTACACATAGCAGACAAATA
HQ219549_A ACACGCTCACGGTTAAAAACGA--------ACATTACTGAACAATTAAAAAGAGTGGTTGCTACACATAGCAGACAAATA
HQ219546_A ACACGCTCACGGTTAAAAACGA--------ACATTACTGAACAATTAAAAAGAGTGGTTGCTACACATAGCAGACAAATA
HQ219540_A ACACGCTCACGGTTAAAAACGA--------ACATTACTGAACAATTAAAAAGAGTGGTTGCTACACATAGCAGACAAATA
HQ219545_A ACACGCTCACGGTTAAAAACGA--------ACATTACTGAACAATTAAAAAGAGTGGTTGCTACACATAGCAGACAAATA
HQ219543_B ACACGCTCATGTTT-AATACGT--------ACATAACTAAACGATTAAAATGTGTG-TTGCTACACCTAACAGGCAAATA
HQ219539_B ACACGCTCATGTTT-AATACGA--------ACATAACTAAACGATTAAAATGTGTG-TTGCTACACCTAACTGACAAATA
HQ219536_B ACACGCTCACGGTTAAAAACGA--------ACATAACTAAATGATTAAAATGCATGGTTGCTACACCTAACTGACAAATA
HQ219547_B ACACGCTCATGCT--AATACGTACATTGGTACATAACTAAACAATTAAAATGCATGGTTGCTTCGCCTAACAGGCAAATA
HQ219538_B ACACGCTCATGCT--AATACGTACATTGGTACATAACTAAACAATTAAAATGCATGGTTGCTTCGCCTAACAGGCAAATA
HQ219534_B ACACGCTCACGGTT-AAAACGA--------ACATAACCAAACGATTAAAAAGCGTGGTTGCTACACCTAACAGGCAAATA
HQ219535_B ACACGCTCACGGTTAAAAACGA--------ACATAACCAAACGATTAAAAAGCGTGGTTGCTACACCTAACAGGCAAATA
```

```
HQ219541_A CCTTTACAAGTTAACACACACTTATTAGTTATTACTGTAATAATAATTATG-----AAAAAAAACAA-----TATTTTTA
HQ219537_A CCTTTACAAGTTAACACACACCTATTAGTTATTACTGTAATAATAATTATG----AAACAAAAACAA-----TATTTTTA
HQ219553_A CCTTTACAAGTTAACACACACTTATTAGTTATTACTGTAATAATAATTATG-----AAAAAAAACAA-----TATTTTTA
HQ219542_A CCTTTACAAGTTAACACACACTTATTAGTTATTACTGTAATAATAATTATG-----AAAAAAAACAA-----TATTTTTA
HQ219531_A CCTTTACAAGTTAACACACACTTATTAGTTATTACTGTAATAATAATTATG-----AAAAAAAACAA-----TATTTTTA
HQ219550_A CCTTTACAAGTTAACACACACTTATTAGTTATTACTGTAATAATAATTATG-----AAAAAAAACAA-----TATTTTTA
HT         CCTTTACAAGTTAACACACACTTATTAGTTATTACTGTAATAATAATTATG-----AAAAAAAACAA-----TATTTTTA
HQ219544_A CCTTTACAAGTTAACACACACTTATTAGTTATTACTGTAATAATAATTATG-----AAAAAAAACGA-----TATTTTTA
HQ219549_A CCTTTACAAGTTAACACACACTTATTAGTTATTACTGTAATAATAATTATG-----AAAAAAAACAA-----TATTTTTA
HQ219546_A CCTTTACAAGTTAACACACACTTATTAGTTATTACTGTAATAATAATTATG-----AAAAAAAACAA-----TATTTTTA
HQ219540_A CCTTTACAAGTTAACACACACTTATTAGTTATTACTGTAATAATAATTATG-----AAAAAAAACAA-----TATTTTTA
HQ219545_A CCTTTACAAGTTAACACACACTTATTAGTTATTACTGTAATAATAATTATG-----AAAAAAAACAA-----TATTTTTA
HQ219543_B ACTTTACAAGTTAACACACACTTATTA-------CTGTAATATTAATTA-CATATAAAAAAAAACTATTTCGTATTTTTA
HQ219539_B ACTTTACAAATTAA--CACAATTATTA-------CTGTAATATTAATTA-CATATAAAAAAAAACTATTTCGTATTTTTA
HQ219536_B ACTTTACAATATAA--CACAATTATTA-------CTGTAATATTAATTA---TAT-AAAAAAAACTATTTCGTATTTTTA
HQ219547_B ACTTTACAATATAA--CACAATTATTA-------CTGTAATATTAATTA-CATAT-AAAAAAAACTATTTCGTATTTTTA
HQ219538_B ACTTTACAATATAA--CACAATTATTA-------CTGTAATATTAATTA-CATAT-AAAAAAAACTATTTCGTATTTTTA
HQ219534_B ACTTTACAATATAA--CACAATTATTA-------CTGTAATATTAATTA-CATAT-AAAAAAAACTATTTCGTATTTTTA
HQ219535_B ACTTTACAATATAA--CACAATTATTA-------CTGTAATATTAATTA-CATAT-AAAAAAAACTATTTCGTATTTTTA

HQ219541_A CTGTAGGGTTAGGCTTTGGCGTTAAGTTATGGAA-TGTTATTACACGGTTTGCATGTACGTTTTTCCCTAGTGTAAAACA
HQ219537_A CTCTAGGGTTAGGCTTTGGCGTTAAGTTATGGAA-TGTTATTACACGATTTGCATATACGTTTTTTCCTAGTGTAAAACA
HQ219553_A CTGTAGGGTTAGGCTTTGGCGTTAAGTTATGGAA-TGTTATTACACGGTTTGCATGTACGTTTTTCCCTAGTGTAAAACA
HQ219542_A CTGTAGGGTTAGGCTTTGGCGTTAAGTTATGGAA-TGTTATTACACGGTTTGCATGTACGTTTTTTCCTAGTGTAAAACA
HQ219531_A CTGTAGGGTTAGGCTTTGGCGTTAAGTTATGGAA-TGTTATTACACGGTTTGCATGTACGTTTTTCCCTAGTGTAAAACA
HQ219550_A CTGTAGGGTTAGGCTTTGGCGTTAAGTTATGGAA-TGTTATTACACGGTTTGCATGTACGTTTTTTCCTAGTGTAAAACA
HT         CTGTAGGGTTAGGCTTTGGCGTTAAGTTATGGAA-TGTTATTACACGGTTTGCATGTACGTTTTTTCCTAGTGTAAAACA
HQ219544_A CTGTAGGGTTAGGCTTTGGCGTTAAGTTATGGAA-TGTTATTACACGGTTTGCATGTACGTTTTTCCCTAGTGTAAAACA
HQ219549_A CTGTAGGGTTAGGCTTTGGCGTTAAGTTATGGAA-TGTTATTACACGGTTTGCATGTACGTTTTTCCCTAGTGTAAAACA
HQ219546_A CTGTAGGGTTAGGCTTTGGCGTTAAGTTATGGAA-TGTTATTACACGGTTTGCATGTACGTTTTTCCCTAGTGTAAAACA
HQ219540_A CTGTAGGGTTAGGCTTTGGCGTTAAGTTATGGAA-TGTTATTACACGGTTTGCATGTACGTTTTTCCCTAGTGTAAAACA
HQ219545_A CTGTAGGGTTAGGCTTTGGCGTTAAGTTATGGAA-TGTTATTACACGGTTTGCATGTACGTTTTTCCCTAGTGTAAAACA
HQ219543_B CATTACGG------TTTGGCGTTAAGGTATAGAA-ATTTATTACACGATTTGCATATACGTTTTATCC-ATTGTAAAACA
HQ219539_B CATTACGG------TTTGGCGTTAAGGTATAGAA-TTTTATTACACGATTTGCATATACGTTTTATCC-ATTGTAAAACA
HQ219536_B CATTAGGG------TTTGGCGTTAAGGTATAGAAATTTTAATACACGATTTGCATATACGTTTTATCC-ATTGTAAAACA
HQ219547_B CATTACGG------TTTGGCGTTAAGGTATAGAA-ATTTATTACACCATTTGCATATACGTTTTAACC-ATTGTAAAACA
HQ219538_B CATTACGG------TTTGGCGTTAAGGTATAGAA-ATTTATTACACCATTTGCATATACGTTTTAACC-ATTGTAAAACA
HQ219534_B CATTACGG------TTTGGCGTTAAGGTATAGAA-ATTTATTACACCATTTGCATATACGTTTTAACC-ATTGTAAAACA
HQ219535_B CATTACGG------TTTGGCGTTAAGGTATAGAA-ATTTATTACACCATTTGCATATACGTTTTAACC-ATTGTAAAACA

HQ219541_A GGACACAATTATACCGTAGCCTATGCTATATAA-GGTTAAGGTATTTTAATAAATTAAGAAATATAAATTTAATATTTAT
HQ219537_A GGACACAATTATACCGTAGCCTATGCTATATAA-GGTTAAGGTATTTTAATAAATTAGAAAATATAAATTTAATATTTAT
HQ219553_A GGACACAATTATACCGTAGCCTATGCTATATAA-GGTTAAGGTATTTTAATAAATTAAGAAATATAAATTTAATACTTAT
HQ219542_A GGACACAATTATACCGTAGCCTATGCTATATAA-GGTTAAGGTATTTTAATAAATTAAGAAATATAAATTTAATACTTAT
HQ219531_A GGACACAATTATACCGTAGCCTATGCTATATAA-GGTTAAGGTATTTTAATAAATTAAGAAATATAAATTTAATATTTAT
HQ219550_A GGACACAATTATACCGTAGCCTATGCTATATAA-GGTTAAGGTATTTTAATAAATTAAGAAATATAAATTTAATATTTAT
HT         GGACACAATTATACCGTAGCCTATGCTATATAA-GGTTAAGGTATTTTAATAAATTAAGAAATATAAATTTAATATTTAT
HQ219544_A GGACACAATTATACCGTAGCCTACGCTATATAA-GGTTAAGGTATTTTAATAAATTAAGAAATATAAATTTAATATTTAT
HQ219549_A GGACACAATTATACCGTAGCCTATGCTATATAA-GGTTAAGGTATTTTAATAAATTAAGAAATATAAATTTAATATTTAT
HQ219546_A GGACACAATTATACCGTAGCCTATGCTATATAA-GGTTAAGGTATTTTAATAAATTAAGAAATATAAATTTAATATTTAT
HQ219540_A GGACACAATTATACCGTAGCCTATGCTATATAA-GGTTAAGGTATTTTAATAAATTAAGAAATATAAATTTAATATTTAT
HQ219545_A GGACACAATTATACCGTAGCCTATGCTATATAA-GGTTAAGGTATTTTAATAAATTAAGAAATATAAATTTAATATTTAT
HQ219543_B GGGCAAAATTATACCGTAGCCTACGCTATATAAAGGTCCAGATATTTTAATAAATTAAGAAAAATAAATTTAATACTTAT
HQ219539_B GGGCAAAATTATACCGTAGCCTATGCTATATAAAGGTCAAAATATTTTAATAAATTAAGAAATATAAATTGAATACTTAT
HQ219536_B GGGCAAAATTATACCGTAGCCTACGCTATATAAAGGTCCAGATATTTTAATAAATTAAGAAAAATAAATTTAATACTTAT
HQ219547_B GGGCAAAATTATACCGTAGCCTATGCTATATAAAGGTCAAAATATTTTAATAAATTAAGAAATATAAATTGAATACTTAT
HQ219538_B GGGCAAAATTATACCGTAGCCTATGCTATATAAAGGTCAAAATATTGTAATAAATTAAGAAATATAAATTGAATACTTAT
HQ219534_B GGGCAAAATTATACCGTAGCCTATGCTATATAAAGGTCAAAATATTTTAATAAATTAAGAAATATAAATTGAATACTTAT
HQ219535_B GGGCAAAATTATACCGTAGCCTATGCTATATAAAGGTCAAAATATTTTAATAAATTAAGAAATATAAATTGAATACTTAT

HQ219541_A TGATTTTGAATTAAATAAACAGGAAGTGGCACACAGGTAACAATATTTTATTAGGAATTATATTTTAATTAAATATTTCT
HQ219537_A TGATTTTGAATTAAATAAACAGGAAGTGGCACACAGGTTACAATATTTTATTAGGAATTATATTTTAATTAAATATTTCT
HQ219553_A TGATTTTGAATTAAATAAACAGGATGTGGCATAAAGGTTACAATATTTTATTGGGAATTAAATTTTAATTAAATATTTCT
HQ219542_A TGATTTTGAATTAAATAAACAGGATGTGGCATAAAGGTTACAATATTTTATTGGGAATTAAATTTTAATTAAATATTTCT
HQ219531_A TGATTTTGAATTAAATAAACAGGATGTGGCATAAAGGTAACAATATTTTATTAGGAATTATATTTTAATTAAATATTTCT
HQ219550_A TGATTTTGAATTAAATAAACAGGAAGTGGCACACAGGTAACAATATTTTATTAGGAATTATATTTTAATTAAATATTTCT
HT         TGATTTTGAATTAAATAAACAGGAAGTGGCACACAGGTAACAATATTTTATTAGGAATTATATTTTAATTAAATATTTCT
HQ219544_A TGATTTTGAATTAAATAAACAGGAAGTGGCACACAGGTAACAATATTTTATTAGGAATTATATTTTAATTAAATATTTCT
HQ219549_A TGATTTTGAATTAAATAAACAGGAAGTGGCACACAGGTAACAATATTTTATTAGGAATTATATTTTAATTAAATATTTCT
HQ219546_A TGATTTTGAATTAAATAAACAGGAAGTGGCACACAGGTAACAATATTTTATTAGGAATTATATTTTAATTAAATATTTCT
HQ219540_A TGATTTTGAATTAAATAAACAGGAAGTGGCACACAGGTAACAATATTTTATTAGGAATTATATTTTAATTAAATATTTCT
HQ219545_A TGATTTTGAATTAAATAAACAGGAAGTGGCACACAGGTAACAATATTTTATTAGGAATTATATTTTAATTAAATATTTCT
HQ219543_B TGATTTTGAATTAAATAAACAGGATGTGGCCTAAAGGTTACAATATTTTATTGGGAATTAAATTTTAATTAAATATTTCT
HQ219539_B TGATTTTGAATTAAATAAACAGGATGTGGCATAAAGGTTACAATATTTTATTGGGAATTAAATTTTAATTAAATATTTCT
HQ219536_B TGATTTTGAACTAAATAAACAGGATGTGGCCTAAAGGTTACAATGTTTTATTGGGAATTATATTTTAATTAAATATTTCT
HQ219547_B TGATTTTGAATTAAATAAACAGGATGTGGCATAAAGGTTACAATATTTTATTGGGAATTAAATTTTAATTAAATATTTCT
HQ219538_B TGATTTTGAATTAAATAAACAGGATGTGGCATAAAGGTTACAATATTTTATTGGGAATTAAATTTTAATTAAATATTTCT
HQ219534_B TGATTTTGAATTAAATAAACAGGATGTGGCATAAAGGTTACAATATTTTATTGGGAATTAAATTTTAATTAAATATTTCT
HQ219535_B TGATTTTGAATTAAATAAACAGGATGTGGCATAAAGGTTACAATATTTTATTGGGAATTAAATTTTAATTAAATATTTCT

HQ219541_A TCGGGGGGTTTGTTTGGATATCATAAATTGTTACGGAAGGGAAGGTTTTATGACGTAATAAATAAGA-------------
HQ219537_A TCGGGGGGTTTGTTTGGATATCATAAATTGTTACGGAAGGGAAGGTTTTATGACGTAATAAATAAGA-------------
HQ219553_A TTGAGGGGTTTGTTTGGATATCATAAATTGTTACGGAAGGGAAGGTTTTATGACGTAATAAATAAGATGCTTTGT-----
HQ219542_A TTGAGGGGTTTGTTTGGATATCATAAATTGTTACGGAAGGGAAGGTTTTATGACGTAATAAATAAGATGCTTTGT-----
HQ219531_A TTGGGGGGTTTGTTTGGATATCATAAATTGTTACGGAAGGGAAGGTTTTATGACGTAATAAATAAGAT------------
HQ219550_A TCGGGGGGTTTGTTTGGATATCATAAATTGTTACGGAAGGGAAGGTTTTATGACGTAATCACTAAGG-------------
HT         TCGGGGGGTTTGTTTGGATATCATAAATTGTTACGGAAGGGAAGGTTTTATGACGTAATAAATAAGA-------------
HQ219544_A TCGGGGGGTTTGTTTGGATATCATAAATTGTTACGGAAGGGAAGGTTTTATGACGTAATAAATAAGA-------------
HQ219549_A TCGGGGGGTTTGTTTGGATATCATAAATTGTTACGGAAGGGAAGGTTTTATGACGTAATAAATAAGA-------------
HQ219546_A TCGGGGGGTTTGTTTGGATATCATAAATTGTTACGGAAGGGAAGGTTTTATGACGTAATAAATAAGA-------------
HQ219540_A TCGGGGGGTTTGTTTGGATATCATAAATTGTTACGGAAGGGAAGGTTTTATGACGTAATAAATAAGA-------------
HQ219545_A TCGGGGGGTTTGTTTGGATATCATAAATTGTTACGGAAGGGAAGGTTTTATGACGTAATAAATAAGA-------------
HQ219543_B TCGAGGGGTTTGTTTGGATATCATAAATTGTTACGGAAGGGAAGTTTTTATGACGTAATAAATAAGATGCTTTGTTCGTC
HQ219539_B TTGAGGGGTTTGTTTGGATATCATAAATTGTTACGGAAGGGAAGGTTTTATGACGTAATAAATAAGAAGCTTTGTTCGTC
HQ219536_B TTGAGGGGTTTGTTTGGATATCATAAATTGTTACGGAAGGGAAGGTTTTATGACGTAATAAATAAGAAGCTTTGTTCGTC
HQ219547_B TTGAGGGGTTTGTTTGGATATCATAAATTGTTACGGAAGGGAAGGTTTTATGACGTAATAAATAAGATGCTTTGTTCGTC
HQ219538_B TTGAGGGGTTTGTTTGGATATCATAAATTGTTACGGAAGGGAAGGTTTTATGACCTAATAAATAAGAAGCTTTGTTCGTC
HQ219534_B TTGAGGGGTTTGTTTGGATATCATAAATTGTTACGGAAGGGAAGGTTTTATGACGTAATAAATAAGATGCTTTGTTCGTC
HQ219535_B TTGAGGGGTTTGTTTGGATATCATAAATTGTTACGGAAGGGAAGGTTTTATGACGTAATAAATAAGATGCTTTGTTCGTC
```

```
HQ219541_A -TACAGA
HQ219537_A -TACAGA
HQ219553_A -TACAGA
HQ219542_A -TACAGA
HQ219531_A -TACAGA
HQ219550_A -TGCAGA
HT         -TACAGA
HQ219544_A -TACAGA
HQ219549_A -TACAGA
HQ219546_A -TACAGA
HQ219540_A -TACAGA
HQ219545_A -TACAGA
HQ219543_B ATACAGA
HQ219539_B ATACAGA
HQ219536_B ATACAGA
HQ219547_B ATACAGA
HQ219538_B ATACAGA
HQ219534_B ATACAGA
HQ219535_B ATACAGA
```

Fkh Alignment

```
HQ219561_A_AAG-AGGAA-GTGGGCGCTACTCCAAAGTCGGCATTCCGTCTTATAAGAGAGCTGGAATTTATTGGAAGCACAATTCAGT
HT         AAGAAGGAAAGTGGGCGCTACTCCAAAGTCGGCATTCCGTCTTATAAGAGAGCTGGAATTTATTGGAAGCACAATTCAGT
HQ219572_A_AAGAAGGAAAGTGGGCGCTACTCCAAAGTCGGCATTCCGTCTTATAAGAGAGCTGGAATTTATTGGAAGCACAATTCAGT
HQ219569_A_AAGAAGGAAAGTGGGCGCTACTCCAAAGTCGGCATTCCGTCTTATAAGAGAGCTGGAATTTATTGGAAGCACAATTCAGT
HQ219568_A_AAGAAGGAAAGTGGGCGCTACTCCAAAGTCGGCATTCCGTCTTATAAGAGAGCTGGAATTTATTGGAAGCACAATTCAGT
HQ219555_A_AAGAAGGAAAGTGGGCGCTACTCCAAAGTCGGCATTCCGTCTTATAAGAGAGCTGGAATTTATTGGAAGCACAATTCAGT
HQ219567_A_AAGAAGGAAAGTGGGCGCTACTCCAAAGTCGGCATTCCGTCTTATAAGAGAACTGGAATTTATTGGAAGCACAATTCAGT
HQ219564_A_AAGAAGGAAAGTGGGCGCTACTCCAAAGTCGGCATTCCGTCTTATAAGAGAGCTGGAATTTATTGGAAGCACAATTCAGT
HQ219563_A_AAGAAGGAAAGTGGGCGCTACTCCAAAGTCGGCATTCCGTCTTATAAGAGAGCTGGAATTTATTGGAAGCACAATTCAGT
HQ219554_A_AAGAAGGAAAGTGGGCGCTACTCCAAAGTCGGCATTCCGTCTTATAAGAGAGCTGGAATTTATTGGAAGCACAATTCAGT
HQ219558_B_AAGAAGGAAAGTGGGCGCTACTCCAAAGTCGGCATTCCGTCTTATAAGAGAACTGGAATTTATTGGAAGCACAATTCAGT
HQ219559_B_AAGAAGGAAAGTGGGCGCTACTCCAAAGTCGGCATTCCGTCTTATAAGAGAACTGGAATTTATTGGAAGCACAATTCAGT
HQ219560_B_AAGAAGGAAAGTGGGCGCTACTCCAAAGTCGGCATTCCGTCTTATAAGAGAACTGGAATTTATTGGAAGCACAATTTAGT
HQ219573_B_AAAAAGGAA-GTGGGCGCTACTCCAAAGTCGGCATTCCGTCTTATAAGAGAACTGGAATTTATTGGAAGCACAATTCAGT
HQ219562_B_AAGAAGGAAAGTGGGCGCTACTCCAAAGTCGGCATTCCGTCTTATAAGAGAACTGGAATTTATTGGAAGCACAATTCAGT
HQ219575_B_AAGAAGGAAAGTGGGCGCTACTCCAAAGTCGGCATTCCGTCTTATAAGAGAACTGGAATTTATTGGAAGCACAATTCAGT
HQ219566_B_AAGAAGGAAAGTGGGCGCTACTCCAAAGTCGGCATTCCGTCTTATAAGAGAACTGGAATTTATTGGAAGCACAATTCAGT

HQ219561_A_TTGTTCAATAGTGAACAGGGTAGTGGTTTCGTATTTAATTTAGATCTACATCTTACTTGAATTAGTATAGGTTTTCACTA
HT         TTGTTCAATAGTGAACAGTGTAGTGGTTTCGTATTTAATTTAGATCTACATCTTACTTGAATTAGTATAGGTTTTCACTA
HQ219572_A_TTGTTCAATAGTGAACAGTGTAGTGGTTTCGTATTTAATTTAGATCTACATCTTACTTGAATTAGTATAGGTTTTCACTA
HQ219569_A_TTGTTCAATAGTGAACAGTGTAGTGGTTTCGTATTTAATTTAGATCTACATCTTACTTGAATTAGTATAGGTTTTCACTA
HQ219568_A_TTGTTCAATAGTGAACAGTGTAGTGGTTTCGTATTTAATTTAGATCTACATCTTACTTGAATTAGTATAGGTTTTCACTA
HQ219555_A_TTGTTCAATAGTGAACAGTGTAGTGGTTTCGTATTTAATTTAGATCTACATCTAACTTGAATTAGTATAGGTTTTCACTA
HQ219567_A_TTGTTCAATAGTGAACAGTGTAGTGGTTTCGTATTTAATTTAGATCTACATCTTACTTGAATTAGTATAGGTTTTCACTA
HQ219564_A_TTGTTCAATAGTGAACAGTGTAGTGGTTTCGTATTTAATTTAGATCTACATCTTACTTGAATTAGTATAGGTTTTCACTA
HQ219563_A_TTGTTCAATAGTGAACAGTGTAGTGGTTTCGTATTTAATTTAGATCTACATCTTACTTGAATTAGTATAGGTTTTCACTA
HQ219554_A_TTGTTCAATAGTGAACAGTGTAGTGGTTTCGTATTTAATTTAGATCTACATCTTACTTGAATTAGTATAGGTTTTCACTA
HQ219558_B_TTGTTCAATAGGGAACAGTGTAGTGGTTTCGTATTTAATTTAGATCTCTATCTTATTCGAATTAGTATAGGTTTTCACTA
HQ219559_B_TTGTTCAATAGTGAACAGTGTAGTGGTTTCGTATTTAATTTAGATCTACATCTTACTTGAATTAGTATAGGTTTTCACTA
HQ219560_B_TTGTTCAATAGTGAACAGTGTAGTGGTTTCGTATTTAATTTAGATCTATATCTTACTTGAATTAGTATAGGTTTTCACTA
HQ219573_B_TTGTTCAATAGTGAACAGTGTAGTGGTTTCGAATTTAATTTAGATCTACATCTTACTTGAATTAGTATAGGTTTTCACTA
HQ219562_B_TTGTTCAATAGTGAACAGTGTAGTGGTTTCGTATTTAATTTAGATCTATATCTTACTTGAATTAGTATAGGTTTTCACTA
HQ219575_B_TTGTTCAATAGTGAACAGTGTAGTGGTTTCGTATTTAATTTAGATCTACATCTTACTTGAATTAGTATAGGTTTTCACTA
HQ219566_B_TTGTTCAATAGTGAACAGTGTAGTGGTTTCGTATTTAATTTAGATCTACATCTTACTTGAATTAGTATAGGTTTTCACTA

HQ219561_A_AAGAGTCGAATTTAAATCGCAAGTATTTAACAGGTAATTGAGTTAC---TTTTGCGAAATAGAACAATTGCTGGATGCAG
HT         AAGAGTCGAATTTAAATCGCAAGTATTTAATAGGTAATTGAGTTAC---TTTTGCGAAATGGAACAATTGCTGGATGCAG
HQ219572_A_AAGAGTCGAATTTAAATCGCAAGTATTTAACAGGTAATTGAGTTAC---TTTTGCGAAATAGAACAATTGCTGGATGCAG
HQ219569_A_AAGAGTCGAATTTAAATCGCAAGTATTTAACAGGTAATTGAGTTAC---TTTTGCGAAATAGAACAATTGCTGGATGCAG
HQ219568_A_AAGAGTCGAATTTAAATCGCAAGTATTTAACAGGTAATTGAGTTAC---TTTTGCGAAACAGAACAATTGCTGGATGCAG
HQ219555_A_AAGAGTCGAATTTAAATCGCAAGTATTTAACAGGTAATTGAGTTAC---TTTTGCGAAATAGAACAATTGCTGGATGCAG
HQ219567_A_AAGAGTCGAATTTAAATCGCAAGTATTTAACAGGTAATTGAGTTAC---TTTTGCGAAATAGAACAATTGCTGGATGCAG
HQ219564_A_AAGAGTCGAATTTAAATCGCAAGTATTTAACAGGTAATTGAGTTAC---TTTTGCGAAATAGAACAATTGCTGGATGCAG
HQ219563_A_AAGAGTCGAATTTAAATCGCAAGTATTTAACAGGTAATTGAGTTAC---TTTTGCGAAATAGAACAATTGCTGGATGCAG
HQ219554_A_AAGAGTCGAATTTAAATCGCAAGTATTTAACAGGTAATTGAGTTAC---TTTTGCGAAATAGAACAATTGCTGGATGCAG
HQ219558_B_AAGAGTCGAATTTA-TTCGCAAGTATTTAACAGATAATTGAGTTAC--TTTTTGCCAAATAGAATAATTGCTAGATGCAG
HQ219559_B_AAGAGTCGAATTTA-ATCGCAAGTATTTAACAGGTAATTGAGTTAC--ATTTTACGAAATAGAACAATTGCTAGATGCAG
HQ219560_B_-AGAGTCGAATTTA-ATCGCAAGTGTTTAACAAATAATTGAGTTAC--TTTTTGCGAAATAGAACAATTGCTAGATGCAG
HQ219573_B_AAGAGTCGAATTTA-ATCGCAAGTATTTAACAGGTAATTGAGTTAC--TTTTTGCGAAATAGAACAATTGCTAGATGCAG
HQ219562_B_AAGAGTCGAATTTA-ATCGCAAGTATTTAACAGGTAATTCAGTTACTTTTTTTGCGAAATAGAACAATTGATAGATGCAG
HQ219575_B_AAGAGTCGAATTTA-ATCGCAAGTATTTAACAGGTAATTCAGTTACTTTTTTTGCGAAATAGAACAATTGATAAATGCAG
HQ219566_B_AAGAGTCGAATTTA-ATCGCAAGTATTTAACAGGTAATTCAGTTACTTTTTTTGCGAAATAGAACAATTGATAGATGCAG

HQ219561_A_TTAGCTTAGAAATACTTG---------CAG----CCTAAAATTTGATTCAGAATTATTAAAGTT----------TTAAAC
HT         TTAGCTTAGAAATACTTGCAGCCCTTGCAG----CCTAAAATTTGATTCAGAATTATTAAAGTT----------TTAAAC
HQ219572_A_TTAGCTTAGAAATACTTG---------CAG----CCTAAAATTTGATTCAGAATTATTAAAGTT----------TTAAAC
HQ219569_A_TTAGCTTAGAAATACTTG---------CAG----CCTAAAATTTGATTCAGAATTATTAAAGTT----------TTAAAC
HQ219568_A_TTAGCTTAGAAATACTTG---------CAG----CCTAAAATTTGATTCAGAATTATTAAAGTT----------TTAAAC
HQ219555_A_TTAGCTTAGAAATACTTG---------CAG----CCTAAAATTTGATTCAGAATTATTAAAGTT----------TTAAAC
HQ219567_A_TTAGCTTAGAAATACTTG---------CAG----CCTAAAATTTGATTCAGAATTATTAAAGTT----------TTAAAC
HQ219564_A_TTAGCTTAGAAATACTTG---------CAG----CCTAAAATTTGATTCAGAATTATTAAAGTT----------TTAAAC
HQ219563_A_TTAGCTTAGAAATACTTG---------CAG----CCTAAAATTTGATTCAGAATTATTAAAGTT----------TTAAAC
HQ219554_A_TTAGCTTAGAAATACTTG---------CAG----CCTAAAATTTGATTCAGAATTATTAAAGTT----------TTAAAC
HQ219558_B_TTAGCTTAGAAATGTTTG---------CAG----CTTAAAATTCGATCCAGAATTATTGAAGTTTTAAAACTTATTAAAC
HQ219559_B_TTAGCTTAGAAATACTTG---------CAG----CCTAAAATTCGATCCAGAATTATAGAAGTTTTAAAACTTATTAAAC
HQ219560_B_TTAGCTTAGAAATGTTTG---------CAG----CCTAAAATTCGATCCAGAATTATTGAAGTTTTAAAACTTATTAAAC
HQ219573_B_TTAGCTTAGAAATGTTTG---------CAG----CCTAAAATTCGATCCAGAATTATAGAAGTTTTAAAACTTATTAAAC
HQ219562_B_ATAGCTTAGAAATGTTTG---------CAG----CCTAAAATTCGATCCAGAATTATTGAAGTTTTAAAACTCATTAAAC
HQ219575_B_TTAGCTTAGAAATGTTTG---------CAG----CCTAAAATTCGATCCAGAATTATCGAAGTTTTAA-ACTTATTAAAC
HQ219566_B_TTAGCTTAGAAATATTTG---------CAG----CCTAAAATTAGATCCAGAATTATTGAAGTTTTAA-ACTTATTAAAC

HQ219561_A_TTTAACCGCAAGAGGTCGCCAAATATTCAGTAGAATAAGCACAAAACTATTGCTGGTTAAATAGTGAGTGCTCCGTTACT
HT         TTTAACCGCAAGAGGTCGCCAAATATTCAGTAGAATAAGCACAAAACTATTGCTGGTTAAATAGTGAGTGCTCCGTTACT
HQ219572_A_TTTAACCGCAAGAGGTCGCCAAATATTCAGTAGAATAAGCACAAAACTATTGCTGGTTAAATAGTGAGTGCTCCGTTACT
HQ219569_A_TTTAACCGCAAGAGGTCGCCAAATATTCAGTAGAATAAGCACAAAACTATTGCTGGTTAAATAGTGAGTGCTCCGTTACT
HQ219568_A_TTTAACCGCAAGAGGTCGCCAAATATTCAGTAGAATAAGCACAAAACTATTGCTGGTTAAATAGTGAGTGCTCCGTTACT
HQ219555_A_TTTAACCGCAAGAGGTCGCCAAATATTCAGTAGAATAAGCACAAAACTATTGCTGGTTAAATAGTGAGTGCTCCGTTACT
HQ219567_A_TTTAACCGCAAGAGGTCGCCAAATATTCAGTAGAATAAGCACAAAACTATTGCTGGTTAAATAGTGAGTGCTCCGTTACT
HQ219564_A_TTTAACCGCAAGAGGTCGCCAAATATTCAGTAGAATAAGCACAAAACTATTGCTGGTTAAATAGTGAGTGCTCCGTTACT
HQ219563_A_TTTAACCGCAAGAGGTCGCCAAATATTCAGTAGAATAAGCACAAAACTATTGCTGGTTAAATAGTGAGTGCTCCGTTACT
HQ219554_A_TTTAACCGCAAGAGGTCGCCAAATATTCAGTAGAATAAGCACAAAACTATTGCTGGTTAAATAGTGAGTGCTCCGTTACT
HQ219558_B_TTTACCCGCAAGAGGTCGCCAAATACTCAGTAGAATAAGCACAAAACTATTGCTGGTTAAATAGTGAGTGCTCCGTTACT
HQ219559_B_TTTACCCGCAAGAGGTCGCCAAATATTCAGTAGAATAAGCACAAAACTATTGCTGGTTAAATAGTGAGTGCTCTGTTACT
HQ219560_B_TTTACCCGCAAGAGGTCGCCAAATACTCAGTAGAATAAGCACAAAACTATTACTGGTTAAATAGTGAGTGCTCCGTTACT
HQ219573_B_TTTACCCGCAAGAGGTCGCCAAATATTCAGTAGAATAAGCACAAAACTATTGCTGGTTAAATAGTGAGTGCTCCGTTACT
HQ219562_B_TTTATCCGCAAGAGGTCGCCAAATATTCAGTAGAATAAGCACAAAACTATTGCCGGTTAAATAGTGAGTTCTCCGTTACT
HQ219575_B_TTTACCCGCAAGGGGTCGCCAAATACTCAGTAGAATAAGCACAAAAGTATTGCTGGTTAAATAGTGAGTGCTCCGTTACT
HQ219566_B_TTTACCCGCAAGAGGTCGCCACATACTCAGTAGAATAAGCACAAAACTATTGCTGGTTAAATAGTGGGTGCTCCGTTACT

HQ219561_A_ACTTGTTGTTTGCGTCCGGAGAGATTTGCGCTGAACCACGTCGACTTTAATTCAGGTGTCGATTCAATTACCAGCTTTAT
HT         ACTTGTTGTTTGCGTCCGGAGAGATTTGCGCTGAACCACGTCGACTTTAATTCAGGTGTCGATTCAATTACCAGCTTTAT
HQ219572_A_ACTTGTTGTTTGCGTCCGGAGAGATTTGCGCTGAACCACGTCGACTTTAATTCAGGTGTCGATTCAATTACCAGCTTTAT
HQ219569_A_ACTTGTTGTTTGCGTCCGGAGAGATTTGCGCTGAACCACGTCGACTTTAATTCAGGTGTCGATTCAATTACCAGCTTTAT
HQ219568_A_ACTTGTTGTTTGCGTCCGGAGAGATTTGCGCTGAACCACGTCGACTTTAATTCAGGTGTCGATTCAATTACCAGCTTTAT
HQ219555_A_ACTTGTTGTTTGCGTCCGGAGAGATTTGCGCTGAACCACGTCGACTTTAATTCAGGTGTCGATTCAATTACCAGCTTTAT
HQ219567_A_ACTTGTTGTTTGCGTCCGGAGAGATTTGCGCTGAACCACGTCGACTTTAATTCAGGTGTCGATTCAATTACCAGCTTTAT
HQ219564_A_ACTTGTTGTTTGCGTCCGGAGAGATTTGCGCTGAACCACGTCGACTTTAATTCAGGTGTCGATTCAATTACCAGCTTTAT
HQ219563_A_ACTTGTTGTTTGCGTCCGGAGAGATTTGCGCTGAACCACGTCGACTTTAATTCAGGTGTCGATTCAATTACCAGCTTTAT
HQ219554_A_ACTTGTTGTTTGCGTCCGGAGAGATTTGCGCTGAACCACGTCGACTTTAATTCAGGTGTCGATTCAATTACCAGCTTTAT
HQ219558_B_ACTTGTTGTTTGCGTCCGGGGAGATTTGCGCTGAACCACGGCGACTTTAATTCAGGTGTCGATTCAATTACCAG-TTTAT
HQ219559_B_ACTTGTTGTTTGCGTCCGGGGAGATTTGCGCTGAACCACGTCGACTTTAATTCAGGTGTCGATTCAATTACCAG-TTTAT
HQ219560_B_ACTTGTTGTTTGCGTCCGGGGAGATTTGCGCTGAACCACGGCGACTTTAATTCAGGTGTCGATTCAATTACCAGCTTTAT
HQ219573_B_ACTTGTTGTTTGCGTCCGGGGAGATTTGCGCTGAACCACGGCGACTTTAATTCAGGTGTCGATTCAATTACCAGCTTTAT
HQ219562_B_ACTTGTTGTTTGCGTCCGGAGAGATTTGCGCTGAACCACGGCGACTTTAATTCAGGTGTCGATTCAATTACTAGCTTTAT
HQ219575_B_ACTTGTTGTTTGCGCCCGGGGAGATTTGCGCTGAACCACGGCGACTTTAATTCAGGTGTCGATTCAATTACTAGCTTTAT
HQ219566_B_ACTTGTTGTTTGCGTCCGGGGAGATTTGCGCTGAACCACGTCGACTTTAATTCAGGTGTCGATTCAATTACCAG-TTTAT
```

```
HQ219561_A_TAGTTTTATTTACTTCAGCTAGGTGACTCAGAACTGGCTCAAGTTGTATGTGTGTGGTAAGCAAGATTGTGTTGGGGTTA
HT         TAGTTTTATTTACTTCAGCTAGGTGACTCAGAACTGGCTCAAGTTGTATGTGGGGGGTAAGCAAGATTGTGTTGGGGTTA
HQ219572_A_TAGTTTTATTTACTTCAGCTAGGTGACTCAGAACTGGCTCAAGTTGTATGTGTGTGGTAAGCAAGATTGTGTTGGGGTTA
HQ219569_A_TAGTTTTATTTACTTCAGCTAGGTGACTCAGAACTGGCTCAAGTTGTATGTGTGTGGTAAGCAAGATTGTGTTGGGGTTA
HQ219568_A_TAGTTTTATTTACTTCAGCTAGGTGACTCAGAACTGGCTCAAGTTGTATGTGTGTGGTAAGCAAGATTGTGTTGGGGTTA
HQ219555_A_TAGTTTTATTTACCTCAGCTAGGTGACTCAGAACTGGCTCAAGTTGTATGTGTGTGGTAAGCAAGATTGTGTTGGGGTTA
HQ219567_A_TAGTTTTATTTACCTCAGCTAGGTGACTCAGAACTGGCTCAAGTTGTATGTGTGTGGTAAGCAAGATTGTGTTGGGGTTA
HQ219564_A_TAGTTTTATTTACCTCAGCTAGGTGACTCAGAACTGGCTCAAGTTGTATGTGTGTGGTAAGCAAGATTGTGTTGGGGTTA
HQ219563_A_TAGTTTTATTTACCTCAGCTAGGTGACTCAGAACTGGCTCAAGTTGTATGTGTGTGGTAAGCAAGATTGTGTTGGGGTTA
HQ219554_A_TAGTTTTATTTACCTCAGCTAGGTGACTCAGAACTGGCTCAAGTTGTATGTGTGTGGTAAGCAAGATTGTGTTGGGGTTA
HQ219558_B_TAGTTTTATTTACTTCAGCCAGGTGACTCAGAATTGTCTCAAGTTGTATGTGTGTGGTAAGCAAGTCTGTGTTGGGGTTA
HQ219559_B_TAGTTTTATTTACTTCAGTCAGGTGACTCAGAATTGTCTCAAGTTATATGTGTGTGGTAAGCAAGTCTTTGTTGGGGTTA
HQ219560_B_TAGTTTTATTTACTTCAGCAAGGTGACTCAGAATTGTCTCAAGTTATATGTGTGTGGTAAGCAAGTCTTTGTTGGGGTTA
HQ219573_B_TAGTTTTATTTACTTCAGTCAGGTGACTCAGAATTGTCTCAAGTTATATGTGTGTGGTAAACAAGACTGTGTTGGGGTTA
HQ219562_B_TAGTTTTATTTACTTCAGCAAGGTGACTCAGAATTGTCTCAAGTTATATGTGTGTGGCAAACAAGTCTTTGTTGGGGTTA
HQ219575_B_TAGTTTTATTTACCTCAGCCAGGCGACTCAGAATTGTCTCAAGTTATACGTGTGTGGTAAGCAAGACTGTGTTGGGGTTA
HQ219566_B_TAGTTTTATTTACTACAGCAAGGTGACTCAGAATTGTCTCAAGTTATACGTGTGTGGTAAGCAAGTCTTTGTTGGGGTTA

HQ219561_A_CAATCCAGTTATACGCTTCGGTTAACTTAAAACACAATTTAGAATCTTGAAATTTAATATCCGAGAAACAGATTTGTAAC
HT         CAATCCAGTTATACGCTTCGGTTAACTTAAAACACAATTTAGAATCTTGAAATTTAATATCCGAAAAACAGATTTGTAAC
HQ219572_A_CAATCCAGTTATACGCTTCGGTTAACTTAAAACACAATTTAGAATCTTGAAATTTAATATCAGAGAAACAGATTTGTAAC
HQ219569_A_CAATCCAGTTATACGCTTCGGTTAACTTAAAACACAATTTAGAATCTTGAAATTTAATATCCGAGAAACAGATTTGTAAC
HQ219568_A_CAATCCAGTTATACGCTTCGGTTAACTTAAAACACAATTTAGAATCTTGAAATTTAATATCAGAGAAACAGATTTGTAAC
HQ219555_A_CAATCCAGTTATACGCTTCGGTTAACTTAAAACACAATTTAGAATCTTGAAATTTAATATCAGAGAAACAGATTTGTAAC
HQ219567_A_CAATCCAGTTATACGCTTCGGTTAACTTAAAACACAATTTAGAATCTTGAAATTTAATATCAGAGAAACAGATTTGTAAC
HQ219564_A_CAATCCAGTTATACGCTTCGGTTAACTTAAAACACAATTTAGAATCTTGAAATTTAATATCAGAGAAACAGATTTGTAAC
HQ219563_A_CAATCCAGTTATACGCTTCGGTTAACTTAAAACACAATTTAGAATCTTGAAATTTAATATCAGAGAAACAGATTTGTAAC
HQ219554_A_CAATCCAGTTATACGCTTCGGTTAACTTAAAACACAATTTAGAATCTTGAAATTTAATATCAGAGAAACAGATTTGTAAC
HQ219558_B_CAATCCAGTTATACGCTTCGGTTAACTTAAAACGCAATTTAGAATCTTGGAATTTAATATCAGAGAATAAGATTTGTAAC
HQ219559_B_CAATCCAGTTATACGCTTCGGTTAACTTAAAACACAATTTAGAATCTTGGAATTTAATATCAGAGAAAAAGATTTGTAAC
HQ219560_B_CAATCCAGTTATACGCTTCGGTTAACTTAAAACGCAATTTAGAATCTTGGAATTTAATATCAGAGAAAAAGATTTGTAAC
HQ219573_B_CAATCCAGTTATACGCTTCGGTTAACTTAAAACGCAATTTAGAATCTTGGAATTTAATATCAGAGAAAAAGATTTGTAAC
HQ219562_B_CAATCCAGTTATACGCTTCGGTTAACTTAAAACGCAATTTAGAATCTTGGAATTTAATATCAGAGAAAAAGATTTGTAAC
HQ219575_B_CAATCCAGTTATACGCTTCGGTTAACTTAAAACGCAATTTAGAATCTTGGAATTTAATATCAGAGAAAAAGATTTGTAAC
HQ219566_B_CAATCCAGTTATACGCTTCGGTTAACTTAAAACGCAATTTAAAATCTTGAAATTTAATATCAGAGAATAAGATTTGTAAC

HQ219561_A_AGGAAGTTAATTCAGTAGCAAAGCGGAAACTTATCTTGACATATTTTCGTTATTTTCAGTTTTATATCTTTGACCAATAA
HT         AGGAAGTTAATTCAGTAGCAAAGCGGAAACTTATCTTGACATATTTTCGTTATTTTCAGTTTTATATCTTTGACCAATAA
HQ219572_A_AGGAAGTTAATTCAGTAGCAAAGCGGAAACTTATCTTGACATATTTTCGTTATTTTCAGTTTTATATCTTTGACCAATAA
HQ219569_A_AGGAAGTTAATTCAGTAGCAAAGCAGAAACTTATCTTAACATATTTTCGTTATTTTCAGTTTTATATCTTTGACCAATAA
HQ219568_A_AGGAAGTTAATTCAGTAGCAAAGCGGAAACTTATCTTGACATATTTTCGTTATTTTCAGTTTTATATCTTTGACCAATAA
HQ219555_A_AGGAAGTTAATTCAGTAGCAAAGCGGAAACTTATCTTGACATATTTTCGTTATTTTCAGTTTTATATCTTTGACCAATAA
HQ219567_A_AGGAAGTTAATTCAGTAGCAAAGCGGAAACTTATCTTGACATATTTTCGTTATTTTCAGTTTTATATCTTTGACCAATAA
HQ219564_A_AGGAAGTTAATTCAGTAGCAAAGCGGAAACTTATCTTGACATATTTTCGTTATTTTCAGTTTTATATCTTTGACCAATAA
HQ219563_A_AGGAAGTTAATTCAGTAGCAAAGCGGAAACTTATCTTGACATATTTTCGTTATTTTCAGTTTTATATCTTTGACCAATAA
HQ219554_A_AGGAAGTTAATTCAGTAGCAAAGCGGAAACTTATCTTGACATATTTTCGTTATTTTCAGTTTTATATCTTTGACCAATAA
HQ219558_B_AGGAAGTTATTTCAGTAGCAAAGCGGAAACTTAACTTGACATATTTTCGTTATTTTTAGTTTTATATTTTTGACCAATAA
HQ219559_B_AGGAAGTTATTTCAGTAGCAAAGCGGAAACTTAACTTGACATATTTTCGTTATTTTTAGTTTTATATTTTTGACCAATAA
HQ219560_B_AGGAAGTTATTTCAGTAGCAAAGCGGAAACTTAACTTGACTTATTTTCGTTATTTTCAGTTCTATATTTTCGACCAATAA
HQ219573_B_AGGAAGTTATTTCAGTAGCAAAGCGGAAACTTAACTTGACATATTTTCGTTATTTTTAGTTCTATATTTTCGACCAATAA
HQ219562_B_AGGAAGTTATTTCAGTAGCCAAGCGGAAACTTAACTCGACATATTTTCGTTATTTTTAGTTTTATATTTTCGACCAATAA
HQ219575_B_AGGAAGCTATTTCAGTAGCAAAGCGGAAACTTAACTCGACATATTTTCGTTATTTTTAGTTCTATATTTTCGACCAATAA
HQ219566_B_AGGAAGTTATTTCAATAGCAAAGCGGAAACTTAACTCGACATATTTTCGTTATTTTTAGTTTTATATTTTCGACCAATAA

HQ219561_A_TTTCGCCGCCATTTTTTTCTTAAAATATCACGGCCAGTTTTTAACAAGG-TTATTTTTCTAGCTTTCTATTTGAAATATT
HT         TTTCGCCGCCATTTTTTTCTTGAAATATCACGGCCAGTTTTTAACAAG-TTTTTTTTTCTAGCTTTCTATTTGAAATATT
HQ219572_A_TTTCGCCGCCATTTTTTTCTTGAAATATCACGGCCAGTTTTTAACAAG-TTTTTTTTTCTAGCTTTCTATTTGAAATATT
HQ219569_A_TTTCGCCGCCATTTTTTTCTTGAAATATCACGGCCAGTTTTTAACAAG--TTTTTTTTCTAGCTTTCTATTTGAAATATT
HQ219568_A_TTTCGCCGCCATTTTTTTCTTAAAATATCACGGCCAGTTTTTAACAAG--TTATTTTTCTAGCTTTCTATTTGAAATATT
HQ219555_A_TTTCGCCGCCATTTTTTTCTTAAAATATCACGGCCAGTTTTTAACAAG--TTATTTTTCTAGCTTTCTATTTGAAATATT
HQ219567_A_TTTCGCCGCCATTTTTTTCTTAAAATATCACGGCCAGTTTTTAACAAG--TTATTTTTCTAGCTTTCTATTTGAAATATT
HQ219564_A_TTTCGCCGCCATTTTTTTCTTAAAATATCACGGCCAGTTTTTAACAAG--TTATTTTTCTAGCTTTCTATTTGAAATATT
HQ219563_A_TTTCGCCGCCATTTTTTTCTTAAAATATCACGGCCAGTTTTTAACAAG--TTATTTTTCTAGCTTTCTATTTGAAATATT
HQ219554_A_TTTCGCCGCCATTTTTTTCTTAAAATATCACGGCCAGTTTTTAACAAG--TTATTTTTCTAGCTTTCTATTTGAAATATT
HQ219558_B_TTTCGCCGCCATTTTTTTTTCAAAATGCCACGGCCAGTTTTTAACAAGTTTTATTTTTCCAGCTTTCTATTTGAAATATC
HQ219559_B_TTTCGCCGCCATTTTTTTTTCAAAATGCCACTGCCAGTTTTTAACAAGTTTTATTTTTCCAGCTTTCTATTTGAAATATC
HQ219560_B_TTTCGCCGCCATTTTTTTTTCAAAATATCACGGCCAGTTTTTAACAAGTTTTATTTTTCCAGCTTTCTATTTGAAATACC
HQ219573_B_TTTCGCCGCCATTTTTTTTTCAAAATATCACGGCCAGTTTTTAACAAGTTTTATTTTTCCAGCTTTCTATTTGAAATATC
HQ219562_B_TTTCGCCGCCATTTTTTTT---AAATATCACGACCAGTTTTTA-ACAAGTTTTTTTTTTCCAGCTTTCTATTTGAAATACC
HQ219575_B_TTTCGCCGCCATTTTTTTT-CAAAATATTACGACCAGTTTTTAACAAGTTTTATTTTTCCAGCTTTCTATTTGAAATATC
HQ219566_B_TTTCGCCGCCATTTTTTTT-CAAAATATCACGATCAGTTTTTAACAAGTTTTATTTTTCCAGCTTTCTATTTGAAATATC

HQ219561_A_ATACACAACTGCTGCTGTAATTTCTGAGAAAGCTTTTGAAATCGGATTACTTTTGCAAGTACAAAAATGATGTTGTCGTC
HT         ATACACAACTGCTGCTGTAATTTCTGAGAAAGCTTTTGAAATCGGATTACTTTTGCAAGTACAAAAATGATGTTGTCGTC
HQ219572_A_ATACACAACTGCTGCTGTAATTTCTGAGAAAGCTTTTGAAATCGGATTACTTTTGCAAGTACAAAAATGATGTTGTCGTC
HQ219569_A_ATACACAACTGCTGCTGTAATTTCTGAGAAAGCTTTTGAAATCGGATTACTTTTGCAAGTACAAAAATGATGTTGTCGTC
HQ219568_A_ATACACAACTGCTGCTGTAATTTCTGAGAAAGCTTTTGAAATCGGATTACTTTTGCAAGTACAAAAATGATGTTGTCGTC
HQ219555_A_ATACACAACTGCTGCTGTAATTTCTGAGAAAGCTTTTGAAATCGGATTACTTTTGCAAGTACAAAAATGATGTTGTCGTC
HQ219567_A_ATACACAACTGCTGCTGTAATTTCTGAGAAAGCTTTTGAAATCGGATTACTTTTGCAAGTACAAAAATGATGTTGTCGTC
HQ219564_A_ATACACAACTGCTGCTGTAATTTCTGAGAAAGCTTTTGAAATCGGATTACTTTTGCAAGTACAAAAATGATGTTGTCGTC
HQ219563_A_ATACACAACTGCTGCTGTAATTTCTGAGAAAGCTTTTGAAATCGGATTACTTTTGCAAGTACAAAAATGATGTTGTCGTC
HQ219554_A_ATACACAACTGCTGCTGTAATTTCTGAGAAAGCTTTTGAAATCGGATTACTTTTGCAAGTACAAAAATGATGTTGTCGTC
HQ219558_B_CCATACAACTGCTGCTGTAATTTCTGAGAGAGCTTTTGAAATCGGATTACTTTTTCAAGTACAAAAATGATGTTGTCGTC
HQ219559_B_CCATACAACTGCTGCTGTAATTTCTGAGAGAGCTTTTGAAATCGGATTACTTTTGCAAGTACAAAAATGATGTTGTCGTC
HQ219560_B_CAATACAACTGCTGCTGTAATTTCTGAGAGAGCTTTTGAAATCGGATTACTTTTTCAAGTACAAAAATGATGTTGTCGTC
HQ219573_B_CCATACAACTGCTGCTGTAATTTCTGAGAGAGCTTTTGAAATCGGATTACTTTTGCAAGTACAAAAATGATGTTGTCGTC
HQ219562_B_CCATACAACTGCTGCTGTAATTTCTGAGAGAGCTTTTGAAATCGGATTACTTTTGCAAGTACAAAAATGATGTTGTCGTC
HQ219575_B_CCATACAACTGCTGCTGTAATTTCTGAGAGAGCTTTTGAAATCGGATTACTTTTGCAAGTACAAAAATGATGTTGTCGTC
HQ219566_B_CCATACAACTACTGCTGTAATTTCTGAGAGAGCTTTTGAAATCGGATTACTTTTGCAAGTACAAAAATGATGTTGTCGTC

HQ219561_A TCCACCGTCAAAGTACCAACCCTTCCAACAGTCTTATGCTACTGGTATGAACA
HT         TCCACCGTCAAAGTACCAACCCTTCCAACAGTCTTATGCTACTGGTATGAACA
HQ219572_A TCCACCGTCAAAGTACCAACCCTTCCAACAGTCTTATGCTACTGGTATGAACA
HQ219569_A TCCACCGTCAAAGTACCAACCCTTCCAACAGTCTTGTGCTACTGGTATGAACA
HQ219568_A TCCACCGTCAAAGTACCAACCCTTCCAACAGTCTTATGCTACTGGTATGAACA
HQ219555_A TCCACCGTCAAAGTACCAACCCTTCCAACAGTCTTATGCTACTGGTATGAACA
HQ219567_A TCCACCGTCAAAGTACCAACCCTTCCAACAGTCTTATGCTACTGGTATGAACA
HQ219564_A TCCACCGTCAAAGTACCAACCCTTCCAACAGTCTTATGCTACTGGTATGAACA
HQ219563_A TCCACCGTCAAAGTACCAACCCTTCCAACAGTCTTATGCTACTGGTATGAACA
HQ219554_A TCCACCGTCAAAGTACCAACCCTTCCAACAGTCTTATGCTACTGGTATGAACA
HQ219558_B TCCACCGTCAAAGTACCAACCATTCCAACAGTCTTATGCTACTGGTATGAACA
HQ219559_B TCCACCGTCAAAGTACCAACCCTTCCAACAGTCTTATGCTACTGGTATGAACA
HQ219560_B TCCACCGTCAAAGTACCAACCCTTCCAACAGTCTTATGCTACTGGTATGAACA
HQ219573_B TCCACCGTCAAAGTACCAACCCTTCCAACAGTCTTATGCTACTGGTATGAACA
HQ219562_B TCCACCGTCAAAGTATCAACCATTCCAACAGTCTTATGCTACTGGTATGAACA
HQ219575_B TCCACCATCAAAGTACCAACCCTTCCAACAGTCTTATGCTACTGGTATGAACA
HQ219566_B TCCACCGTCAAAGTACCAACCCTTCCAACAGTCTTATGCTACTGGTATGAACA
```

Jade alignment

```
HT          CGATAACATGCAGCTTGCCATCAAAACCAAGGAAGGATTAGGAATCGAATATGACGAAGATGTTGTCTGTGATGTTTGTA
HQ219587_A  CGATAACATGCAGCTTGCCATCAAAACCAAGGAAGGATTAGGAATCGAATATGACGAAGATGTTGTCTGTGATGTTTGTA
HQ219590_A  CGATAACATGCAGCTTGCCACCAAAACCAAGGAAGGATTAGGAATCGAATATGACGAAGATGTTGTCTGTGATGTTTGTA
HQ219582_A  CGATAACATGCAGCTTGCCATCAAAACCAAGGAAGGATTAGGAATCGAATATGACGAAGATGTTGTCTGTGATGTTTGTA
HQ219584_A  CGATAACATGCAGCTTGCCATCAAAACCAAGGAAGGATTAGGAATCGAATATGACGAAGATGTTGTCTGTGATGTTTGTA
HQ219592_A  CGATAACATGCAGCTTGCCATCAAAACCAAGGAAGGATTAGGAATCGAATATGACGAAGATGTTGTCTGTGATGTTTGTA
HQ219599_A  CGATAACATGCAGCTTGCCATCAAAACCAAGGAAGGATTAGGAATCGAATATGACGAAGATGTTGTCTGTGATGTTTGTA
HQ219585_A  CGATAACATGCAGCTTGCCATCAAAACCAAGGAAGGATTAGGAATCGAATATGACGAAGATGTTGTCTGTGATGTTTGTA
HQ219581_B  CGATAACATGCAGCTTGCCATCAAAACCAAGGAAGGTTTAGGAATCGAATATGACGAAGATGTTGTCTGTGATGTTTGTA
HQ219596_B  CGATAACATGCAGCTTGCCATCAAAACCAAGGAAGGATTAGGAATCGAATATGACGAGGATGTTGTCTGTGATGTTTGCA
HQ219583_B  CGATAACATGCAGCTTGCCATCAAAACCAAGGAAGGATTAGGAATCGAATATGACGAGGATGTTGTCTGTGATGTTTGCA
HQ219589_B  CGATAACATGCAGCTTGCCATCAAAACCAAGGAAGGATTAGGAATCGAATATGACGAGGATGTTGTCTGTGATGTTTGCA
HQ219600_B  CGATAACATGCAGCTTGCCATCAAAACCAAGGAAGGATTAGGAATCGAATATGACGAAGATGTTGTCTGTGATGTTTGTA
HQ219591_B  CGATAACATGCAGCTTGCCATCAAAACCAAGGAAGGATTAGGAATCGAATATGATGAAGATGTTGTCTGTGATGTTTGTA
HQ219578_B  CGATAACATGCAGCTTGCCATCAAAACCAAGGAAGGATTAGGAATCGAATATGATGAAGATGTTGTCTGTGATGTTTGTA
HQ219607_B  TGATAACATGCAGCTTGCCATCAAAACCAAGGAAGGATTAGGAATCGAATATGATGAAGATGTTGTCTGTGATGTTTGTA

HT          GGATAGTAAGTTCATTTTT--CCTATTC-TACCATGCGTATAAGTACATTAAAGCCATTGTATAATGCTAGGCAGCTGGG
HQ219587_A  GGATAGTAAGTTCATTTTT--CCTATTC-TACCATGCGTATAAGTACATTAAAGCCATTGTATAACGCTAGGCAT-----
HQ219590_A  GGATAGTAAGTTCATTTTTTTCCTATTC-TACCATGCGTATAAGTACATTAAAGCCATTGTATAACGCTAGGCAT-----
HQ219582_A  GGATAGTAAGTTCATTTTTTTCCTATTCGTACCATGCGTATAAGTACATTAAAGCCATTGTATAACGCTAGGCAT-----
HQ219584_A  GGATAGTAAGTTCATTTTTTTCCTATTC-TACCATGCGTATAAGTACATTAAAGCCATTGTATAACGCTAGGCAT-----
HQ219592_A  GGATAGTAAGTTCATTTTTTTCCTATTC-TACCATGCGTATAAGTACATTAAAGCCATTGTATAACGCTAGGCAT-----
HQ219599_A  GGATAGTAAGTTCATTTTTTTCCTATTC-TACCATGCGTATAAGTACATTAAAGCCATTGTATAACGCTAGGCAT-----
HQ219585_A  GGATAGTAAGTTCATTTTTTTCCTATTC-TACCATGCGTATAAGTACATTAAAGCCATTGTATAACGCTAGGCAT-----
HQ219581_B  GGATAGTAAGTTCATTTTT--CCTATTT-TTGTTTATTTATATGTACATTAAAACCATTGTATAATGCTAAT-GT-----
HQ219596_B  GGATAGTAAGTTCATTTT--CCCCATTT-TTTTTTATGTATAGGTACATTAAAGTCATTGTATAATGCTAAACGT-----
HQ219583_B  GGATAGTAAGTTCATTTT--CCCCATTT-TTTTTTATGTATAGGTACATTAAAGTCATTGTATAATGCTAAACGT-----
HQ219589_B  GGATAGTAAGTTCATTTT--CCCCATTT-TTTTTTATGTATAGGTACATTAAAGTCATTGTATAATGCTAAACGT-----
HQ219600_B  GGATAGTAAGTTCATTTTT--CCTATTC-TATTTTATTTATAAGTAAATTAAAGCCATTGTATAATGCTAAACTT-----
HQ219591_B  GGATAGTAAGTTCATTTTT--CCTATTT-TTGTTTATTTATAAGTACATTAAAGCCATTGTATAATGCTAAACTT-----
HQ219578_B  GGATAGTAAGTTCATTTTT--CCTATTC-TATTTTATTTATAAGTACATTTAAGCCATTGTATAATGCTAAACGT-----
HQ219607_B  GGATAGTAAGTTCATTTTT--CCTATTC-TATTTTATTTATAAGTAAATTAAAGCCATTGTATAATGCTAAACGA-----

HT          CATATTTTATTTGCAA-GGTTGTTAATAC---TTTTTTCCGTTTTTTTGTGCTTAATGTCTGATGATTCATTTTTGATTT
HQ219587_A  ---ATTTTATTTGCAA-GGTTGTTAATACA--TTTTTTTCTTTTTTTTGTGCTTAATGTCTGATGATTCATTTTTGATTT
HQ219590_A  ---ATTTTATTTGCAA-GGTTGTTAATACA--TTTTTTTCTTTTTTTTGTGCTTAATGTCTGATGATTCATTTTTGATTT
HQ219582_A  ---ATTTTATTTGCAA-GGTTGGTTAAATACATTTTTTTCTTTTTTTTGTGCTTAATGTCTGATGATTCA-TTTTGATTT
HQ219584_A  ---ATTTTATTTGCAA-GGTTGTTAATACA--TTTTTTTC-TTTTTTTGTGCTTAATGTCTGATGATTCATTTTTGATTT
HQ219592_A  ---ATTTTATTTGCAA-GGTTGTTAATACA--TTTTTTTCTTTTTTTTGTGCTTAATGTCTGATGATTCATTTTTGATTT
HQ219599_A  ---ATTTTATTTGCAA-GGTTGTTAATACA--TTTTTTTCTTTTTTTTGTGCTTAATGTCTGATGATTCATTTTTGATTT
HQ219585_A  ---ATTTTATTTGCAA-GGTTGTTAATACA--TTTTTTTCTTTTTTTTGTGCTTAATGTCTGATGATTCATTTTTGATTT
HQ219581_B  ---ATTTTTTTTGCAAAGGT-GTTGATAC-------TTTTTTTTTTTTGTGCTTAATGTCTGATGGTTCATTTTTGTTTT
HQ219596_B  ---ATTTT--TTGCAAAGGTT-TTGATAC----TTTTTTCTTTGTTTTATGCTTAATGTCTGATGA----TTTTTGTTTT
HQ219583_B  ---ATTTT--TTGCAAAGGTT-TTGATAC----TTTTTTCTTTGTTTTATGCTTAATGTCTGATGA----TTTTTGTTTT
HQ219589_B  ---ATTTT--TTGCAAAGGTT-TTGATAC----TTTTTTCTTTGTTTTATGCTTAATGTCTGATGA----TTTTTGTTTT
HQ219600_B  ---ATTTT--TTGCAA-GGGTGTTGATAC----TTTTTTCTTTTTTTTGTGCTTAATGACTGATGGTTCATTTTTGTTTT
HQ219591_B  ---ATTTT--TTGCAA-GGGTGTTGATAC--TTTTTTTTCTTTTTTTTGTGCTTAATGACTGATGGTTCATTTTTGTTTT
HQ219578_B  ---ATTTT--TTGCTT-GGGTGTTGATAC--TTTTTTTTCTTTTTTTTGTGCTTAATGTCTGATGGTTCATTTTTGTTTT
HQ219607_B  ---ATTTT-TTTGAAA-GAGTGTTGATAC------TTTTC----TTTTGTGCTTAATGACTGATAGTTCATTTTTATTTT

HT          GACGTATTTCTC------AGTGTGTTTTAA--------CCATCTCATTTTCAATGCAGCCAGATTGTGAGGAAGGGAATG
HQ219587_A  GACGTATTTCTC------AGTATGTTTTAA--------CCATCTCATTTTCAATGCAGCCAGATTGTGAGGAAGGGAATG
HQ219590_A  GACGTATTTTTC------AGTGTGTTTTAA--------CCATCTCATTTTCAATGCAGCCAGATTGTGAGGAAGGGAATG
HQ219582_A  GACGTATTTTTC------AGTGTGTTTTAA--------CCATCTCATTTTCAATGCAGCCAGATTGTGAGGAAGGGAATG
HQ219584_A  GACGTATTTTTC------AGTGTGTTTTAA--------CCATCTCATTTTCAATGCAGCCAGATTGTGAGGAAGGGAATG
HQ219592_A  GACGTATTTTTC------AGTGTGTTTTAA--------CCATCTCATTTTCAATGCAGCCAGATTGTGAGGAAGGGAATG
HQ219599_A  GACGTATTTTTC------AGTGTGTTTTAA--------CCATCTCATTTTCAATGCAGCCAGATTGTGAGGAAGGGAATG
HQ219585_A  GACGTATTTTTC------AGTGTGTTTTAA--------CCATCTCATTTTCAATGCAGCCAGATTGTGAGGAAGGGAATG
HQ219581_B  GATGTATTTCTCAATTTGAATGTATTTTAA--------CCATCTCATTTTCATTGCAGCCAGATTGTGAGGAAGGGAATG
HQ219596_B  GACGTTTTTCTAAATTTGAATGTAT-TTAA--------CCATTCCATTTTCAATGCAGCCAGATTGTGAGGAAGGGAATG
HQ219583_B  GACGTTTTTCTAAATTTGAATGTAT-TTAA--------CCATTCCATTTTCAATGCAGCCAGATTGTGAGGAAGGGAATG
HQ219589_B  GACGTTTTTCTAAATTTGAATGTAT-TTAA--------CCATTCCATTTTCAATGCAGCCAGATTGTGAGGAAGGGAATG
HQ219600_B  GACGTATTTCTTAATTTTAAGGTAT-TTAA--------CCATCTCATTTTCAATGCAGCCAGATTGTGAGGAAGGGAATG
HQ219591_B  GTCGTATTTCTCAATTTGAATGTATTTTAA--------CCATCTCATTTTCAATGCAGCCAGATTGTGAGGAAGGGAATG
HQ219578_B  GACGTATTTCTCAATTTGAATGTATTTTAA--------CCATCTCATTTTCATTGCAGCCAGATTGTGAGGAAGGGAATG
HQ219607_B  GATGTATTTCTCAATTTGAATGTATTTTAAGTTTTTAACCATCTCATTTTCAATGCAGCCTGATTGTGAGGAAGGGAATG

HT          AAATGGTTTTCTGTGATGGGTGTAACTTATGTGTTCACCAAGCTTGTTATGGGATTTTGAAGGTCAGTGTTATCAGTTAT
HQ219587_A  AAATGGTTTTCTGTGATGGATGTAACTTATGTGTTCACCAAGCTTGTTATGGGATTTTGAAGGTCAGTGTTATCAGTTAT
HQ219590_A  AAATGGTTTTCTGTGATGGGTGTAACTTATGTGTTCACCAAGCTTGTTATGGGATTTTGAAGGTCAGTGTTATCAGTTAT
HQ219582_A  AAATGGTTTTCTGTGATGGGTGTAACTTATGTGTTCACCAAGCTTGTTATGGGATTTTGAAGGTCAGTGTTATCAGTTAT
HQ219584_A  AAATGGTTTTCTGTGATGGGTGTAACTTATGTGTTCACCAAGCTTGTTATGGGATTTTGAAGGTCAGTGTTATCAGTTAT
HQ219592_A  AAATGGTTTTCTGTGATGGGTGTAACTTATGTGTTCACCAAGCTTGTTATGGGATTTTGAAGGTCAGTGTTATCAGTTAT
HQ219599_A  AAATGGTTTTCTGTGATGGGTGTAACTTATGTGTTCACCAAGCTTGTTATGGGATTTTGAAGGTCAGTGTTATCAGTTAT
HQ219585_A  AAATGGTTTTCTGTGATGGGTGTAACTTATGTGTTCACCAAGCTTGTTATGGGATTTTGAAGGTCAGTGTTATCAGTTAT
HQ219581_B  AAATGGTTTTCTGTGATGGATGTAACTTATGTGTGCACCAAGCTTGTTATGGAATTTTAAAGGTCAGTGTTATCTCTTAT
HQ219596_B  AAATGGTTTTCTGTGATGGATGTAACTTATGTGTGCACCAAGCTTGTTATGGAATTTTAAAGGTCAGTGTTATCTGTTAT
HQ219583_B  AAATGGTTTTCTGTGATGGATGTAACTTATGTGTGCACCAAGCTTGTTATGGAATTTTAAAGGTCAGTGTTATCTGTTAT
HQ219589_B  AAATGGTTTTCTGTGATGGATGTAACTTATGTGTGCACCAAGCTTGTTATGGAATTTTAAAGGTCAGTGTTATCTGTTAT
HQ219600_B  AAATGGTTTTCTGTGATGGATGTAACTTATGTGTGCACCAAGCTTGTTATGGAATTTTGAAGGTCAGTGTTATCAGTTAT
HQ219591_B  AAATGGTTTTCTGTGATGGATGTAACTTATGTGTGCACCAAGCTTGTTATGGAATTTTAAAGGTCAGTGTTATCTCTTAT
HQ219578_B  AAATGGTTTTCTGTGATGGATGTAACTTATGTGTGCACCAAGCTTGTTATGGAATTTTAAAGGTCAGTGTTATCTCTTAT
HQ219607_B  AAATGGTTTTCTGTGATGGATGTAACTTATGTGTGCACCAAGCTTGTTATGGAATTTTGAAGGTCAGTGTTATCTCTTAT

HT          ------------GCTACAACAAACATAATCAAAAAATTGTTTATGCAGCTTTTTATAATGAATTCGGTACACG-CA----
HQ219587_A  ------------ACTATAACAAACATAATCAAAAAATTGTTTATGCAGCTTTTTATAATGAATTCGGTACACA-CA----
HQ219590_A  ------------GCTACAACAAACATAATCAAAAATTTGTTTATGCAGCTTTTTATAATGAATTCCTTACACA-CA----
HQ219582_A  ------------ACTATAACAAACATAATCAAAAAATTGTTTATGCAGCTTTTTATAATGAATTCAGTACACA-CA----
HQ219584_A  ------------ACTATAACAAACATAATCAAAAAATTGTTTATGCAGCTTTTTATAATGAATTCAGTACACA-CA----
HQ219592_A  ------------ACTATAACAAACATAATCAAAAAATTGTTTATGCAGCTTTTTATAATGAATTCAGTACACA-CA----
HQ219599_A  ------------ACTATAACAAACATAATCAAAAAATTGTTTATGCAGCTTTTTATAATGAATTCAGTACACA-CA----
HQ219585_A  ------------ACTATAACAAACATAATCAAAAAATTGTTTATGCAGCTTTTTATAATGAATTCAGTACACA-CA----
HQ219581_B  GCTTCTTGTTATGCTATATCAAACATAATCAAAAAATTGTTTATGCAGCTTTTTATAATGAATTCGATACACAACAA---
HQ219596_B  GCTTATTT----CTAATATCAAACAAAATCAAAAAAGTGTTTATGCAGCTTTTTATAATGAATTCGATACACA-CA----
HQ219583_B  GCTTATTT----CTAATATCAAACAAAATCAAAAAAGTGTTTATGCAGCTTTTTATAATGAATTCGATACACA-CA----
HQ219589_B  GCTTATTT----CTAATATCAAACAAAATCAAAAAAGTGTTTATGCAGCTTTTTATAATGAATTCGATACACA-CA----
HQ219600_B  GCTTCTTGTTATGCTATATCAAACATAATCAGAAAATTGTTTATGCAGCTTTTTATAATGAATTCGATACACA-CA-CA-
HQ219591_B  GCTTCTTGTTATGCTATATCAAACATAATCAAAAAATTGTTTATGCAGCTTTTTATAATGAATTCGATACACAACAA---
HQ219578_B  GCTTATTT----ATAATATCAAACAAAATCAAAAAAGTGTTTATGCAGCTTTTTATAATGAATTCGATACACA-CATTGA
HQ219607_B  GCTTCTTGTTATGCTATATCAAACATAATCAAAAAAGTGTTTATGCAGCTTTTTATAATGAATTCGATACACA-CAA---
```

```
HT           ---------TTAAAAA-TATTTTAGTATAACGCACCT-----GGGACAAAAATCTTCCGCGGATCTGCTGATTTCAGAAC
HQ219587_A -TTATTTTTTAAAAAA-TATTTTAGTATAACGCACCT-----GGGACGAAAATCTTCCGCGGATCTGCTGATTTCAGAAC
HQ219590_A -TTATTTCTTTTTAAG-TATTTTAGTATAACCCACCTGGCATGGGACAAAAATCTTTCGCAGATCTGCTGATTTCAGAAC
HQ219582_A -TTATTTCTTTTTAAA-TATTTTAGTATAACGCACCT-----GGGACGAAAATCTTCCGCGGATCA--------------
HQ219584_A -TTATTTCTTTTTAAA-TATTTTAGTATAACGCACCT-----GGGACGAAAATCTTCCGCGGATCA--------------
HQ219592_A -TTATTTCTTTTTAAA-TATTTTAGTATAACGCACCT-----GGGACGAAAATCTTCCGCGGATCA--------------
HQ219599_A -TTATTTCTTTTTAAA-TATTTTAGTATAACGCACCT-----GGGACGAAAATCTTCCGCGGATCA--------------
HQ219585_A -TTATTTCTTTTTAAA-TATTTTAGTATAACGCACCT-----GGGACGAAAATCTTCCGCGGATCA--------------
HQ219581_B -TTCTTCT--TTTTGGTTTTCTTAGTATTACACACCT-------------------------------------------
HQ219596_B TTTTTTTC-TTTTGGGTTATCTTAGTGTTATACACCT-------------------------------------------
HQ219583_B TTTTTTTC-TTTTGGGTTATCTTAGTGTTATACACCT-------------------------------------------
HQ219589_B TTTTTTTC-TTTTGGGTTATCTTAGTGTTATACACCT-------------------------------------------
HQ219600_B -TTTTTTCTTTTTGGGTTATCGTAGTATTACACACCT-------------------------------------------
HQ219591_B --TTCTTC-TTTTTGGTTTTCTTAGTATTACACACCT-------------------------------------------
HQ219578_B TTTTCCTC-TTTTGGGTTATCTTAGTATTACACACCT-------------------------------------------
HQ219607_B --TTTTTC-TTTTTGGTTTTCTTAGTATTACACACCT-------------------------------------------

HT           CTTTTTTGTGTTAAAAAGGTCATCGTATAGACTTGTATTTTAGCCTTTACATATGTTGTAGTTTAACCTTTACATGCATC
HQ219587_A CTTTTTTGTGTTAAAAAGGTCATCGTATAGACTTGTATTTTAGCCT-----------------------TTACATGCATC
HQ219590_A CTTTTTTGTGTTAAAAAGGTCATCGTATAGACTTGTATTTTAGCCTTTACATATGTTGTTGTTTAACTTTTACATGCATC
HQ219582_A -----------------GGTCATCGTATAGACTTATATTTTAGCCTTTACACATGTTGTTGTTTAGCCTTTACATGCATC
HQ219584_A -----------------GGTCATCGTATAGACTTATATTTTAGCCTTTACACATGTTGTTGTTTAGCCTTTACATGCATC
HQ219592_A -----------------GGTCATCGTATAGACTTATATTTTAGCCTTTACACATGTTGTTGTTTAGCCTTTACATGCATC
HQ219599_A -----------------GGTCATCGTATAGACTTATATTTTAGCCTTTACACATGTTGTTGTTTAGCCTTTACATGCATC
HQ219585_A -----------------GGTCATCGTATAGACTTATATTTTAGCCTTTACACATGTTGTTGTTTAGCCTTTACATGCATC
HQ219581_B --------------------------------------------------------------------------------
HQ219596_B --------------------------------------------------------------------------------
HQ219583_B --------------------------------------------------------------------------------
HQ219589_B --------------------------------------------------------------------------------
HQ219600_B --------------------------------------------------------------------------------
HQ219591_B --------------------------------------------------------------------------------
HQ219578_B --------------------------------------------------------------------------------
HQ219607_B --------------------------------------------------------------------------------

HT           GTACTTAACGCCCC-AAGACCCCTAGGACAAACATTTTGAGGAATTTGGATC----------------------------
HQ219587_A GTACTTAACGCCCC-AAGACCCCGAGGACAAACATTTTGAGGAATTTTGATC----------------------------
HQ219590_A GTACTTAACGCCCC-AAGACCCCGAGGACAAACATTTTGAGGAATTTTGATCAC--------------------------
HQ219582_A GTACTTAACGCCCC-AAGACCCCGAGGACAAACATTTTGAGGAATTTTGATC----------------------------
HQ219584_A GTACTTAACGCCCC-AAGACCCCGAGGACAAACATTTTGAGGAATTTTGATC----------------------------
HQ219592_A GTACTTAACGCCCC-CAGACCCCGAGGACAAACATTTTGAGGAATTTTGATC----------------------------
HQ219599_A GTACTTAACGCCCC-AAGACCCCGAGGACAAACATTTTGAGGAATTTTGATC----------------------------
HQ219585_A GTACTTAACGCCCC-AAGACCCCGAGGACAAACATTTTGAGGAATTTTGATC----------------------------
HQ219581_B ------------------------------------------------------TAGATTGTGCAAAAGTACTATTTTGC
HQ219596_B ------------------------------------------------------TAGATTGTGCAAAAGTACTATTTTCC
HQ219583_B ------------------------------------------------------TAGATTGTGCAAAAGTACTATTTTCC
HQ219589_B ------------------------------------------------------TAGATTGTGCAAAAGTACTATTTTCC
HQ219600_B ------------------------------------------------------TAGATTGTGCAAAAGTACTATTTTGC
HQ219591_B ------------------------------------------------------TAGATTGTGCAAAAGTACTATTTTGC
HQ219578_B ------------------------------------------------------TAGATTGTGCAAAAGTACTATTTTGC
HQ219607_B ------------------------------------------------------TAGATTGTGCAAAAGTACTATTTTGC

HT           --------------------------------------------------------------------------------
HQ219587_A --------------------------------------------------------------------------------
HQ219590_A --------------------------------------------------------------------------------
HQ219582_A --------------------------------------------------------------------------------
HQ219584_A --------------------------------------------------------------------------------
HQ219592_A --------------------------------------------------------------------------------
HQ219599_A --------------------------------------------------------------------------------
HQ219585_A --------------------------------------------------------------------------------
HQ219581_B AAAAAAAAATTGCTAGCTCGTATTGTGTGCACAATTAAACAAATGTGCGATCCTGTTTATTAATCGCATATAGTTACATG
HQ219596_B AA--AAAAATTGCTAGCTCGTATTGTGTGCACAATTAAACAAATGTGCCATCCTGTTTATTAATCGCATATAGTTACATG
HQ219583_B AA--AAAAATTGCTAGCTCGTATTGTGTGCACAATTAAACAAATGTGCCATCCTGTTTATTAATCGCATATAGTTACATG
HQ219589_B AA--AAAAATTGCTAGCTCGTATTGTGTGCACAATTAAACAAATGTGCCATCCTGTTTATTAATCGCATATAGTTACATG
HQ219600_B AAAGAAAAATTGCTAGCCCGTATTGTGTGCACAATTAAACAAATGTGCCAGCCTGTTTATTAATCGCATATAGTTACATA
HQ219591_B AAA-AAAAATTGCTAGCTCGTATTGTGTGCACAATTAAACAAATGTGCGATCCTGTTTATTAATCGCATATAGTTACATG
HQ219578_B AAA-AAAAATTGCTAGCTCGTATTGTGTGCACAATTAAACAAATGTGCCAGCCTGTTTATTAATCGCATATAGTTACATG
HQ219607_B AAAAAAA-ATTGCTAGCTCGTATTGTGTGCACAATTAAACAAATGTGCCAGCCTGTTTATTAATTGCATACAGTTACATG

HT           --------------------------------------------------------------------------------
HQ219587_A --------------------------------------------------------------------------------
HQ219590_A --------------------------------------------------------------------------------
HQ219582_A --------------------------------------------------------------------------------
HQ219584_A --------------------------------------------------------------------------------
HQ219592_A --------------------------------------------------------------------------------
HQ219599_A --------------------------------------------------------------------------------
HQ219585_A --------------------------------------------------------------------------------
HQ219581_B GCAACGTTAACAAAAGTAATGCTTACAACAGGCATGAGAATCTTCCGTGAATGCTCTGATTTCTGAACCTTCTTCTGTTA
HQ219596_B GCAACGTTACCAAAAGTAATTCTTACAGCAGGGATGAGAATCGTCCGCGGATGCGCTAATTTCCGAATCTTCTTCTGTTA
HQ219583_B GCAACGTTACCAAAAGTAATTCTTACAGCAGGGATGAGAATCGTCCGCGGATGCGCTAATTTCCGAATCTTCTTCTGTTA
HQ219589_B GCAACGTTACCAAAAGTAATTCTTACAGCAGGGATGAGAATCGTCCGCGGATGCGCTAATTTCCGAATCTTCTTCTGTTA
HQ219600_B GCAACGTTACCAAAAGTAATTCTTACAGCAGGGATGAGAATCGTATGCGGATGCACTAATTTCCGAACCTTCTTCTGTTA
HQ219591_B GCAACGTTAACAAAAGTAATGCTTACAACAGGCATGAGAATCTTCCGTGAATGCTCTGATTTCTGAACCTTCTTCTGTTA
HQ219578_B GAAACGTTACCAAAAGTAATTCTTACAATAGGGATGAGAATCGTCCGCGGATGCGCTAATTTCCCAACCTTCTTCTGTTA
HQ219607_B GCAACGTTACCAAAAGTAATGCTTACAATAGGTATGAAATCT-TCCGTGGATGCTCTGATTTCCGAACCTTTTTT-----

HT           --------------------------------------------------------------------------------
HQ219587_A --------------------------------------------------------------------------------
HQ219590_A --------------------------------------------------------------------------------
HQ219582_A --------------------------------------------------------------------------------
HQ219584_A --------------------------------------------------------------------------------
HQ219592_A --------------------------------------------------------------------------------
HQ219599_A --------------------------------------------------------------------------------
HQ219585_A --------------------------------------------------------------------------------
HQ219581_B AAAAGGTCTTCGTCGTATTTTAGCCTTCACATACGTTGAAGTTTAGCCTTTACGTGGGTCGTATTTAACGCCCCCC----
HQ219596_B AAAAGGTCTTCGTCGTATTTTAGCCTTCACATAAGTTGTAGTTTAGCCTTTATGTGCGTCGTATTTAACGCCCC-CAGAC
HQ219583_B AAAAGGTCTTCGTCGTATTTTAGCCTTCACATAAGTTGTAGTTTAGCCTTTATGTGCGTCGTATTTAACGCCCC-CAGAC
HQ219589_B AAAAGGTCTTCGTCGTATTTTAGCCTTCACATAAGTTGTAGTTTAGCCTTTATGTGCGTCGTATTTAACGCCCC-CAGAC
HQ219600_B AAAAGGTCTTCGTCGTATTTTAGCCT-----------------------TT-CATGCGTCGTATTTAACACCCCCAAGAT
HQ219591_B AAAAGGTCTTCGTCGTATTTTAGCCTTCACATACGTTGAAGTTTAGCCTTTACGTGGGTCGTATTTAACACCCCCAAGAT
HQ219578_B AAAAGGTCTTCGTCGTATTTTAGCCTTCACATACGTTGTAGTTTAGCCTTT-CATGCGTCGTATTTAACACCCCCAAGAT
HQ219607_B --------------------------------------------------TACGCGCACCGTATTTAACGCCCCCCAGAC
```

```
HT          ------------------------------------TCTATTCTCATCCTTGTTACAAAATTATCAGCATGCTCACTGAT
HQ219587_A ------------------------------------TCTATTCTCATCCTTGTTACAAAATTATCAGCATGCTCACTGAT
HQ219590_A ------------------------------------TCTGTTCTCATCCTTGTTACAAAATTATCAGCATGCTCACTGAT
HQ219582_A ------------------------------------TCTATTCTCATCCTTGTAACAAAATTATCAGCATGCTCACCGAT
HQ219584_A ------------------------------------TCTATTCTCATCCTTGTAACAAAATTATCAGCATGCACACAGAT
HQ219592_A ------------------------------------TCTATTCTCATCCTTGTAACAAAATTATCAGCATGCTCACCGAT
HQ219599_A ------------------------------------TCTATTCTCATCCTTGTAACAAAATTATCAGCATGCTCACTGAT
HQ219585_A ------------------------------------TCTATTCTCATCCTTGTAACAAAATTATCAGCATGCTCACCGAT
HQ219581_B -----CTACAAACATTTTCAAAAATTTTGAATAAACTCAATTCTCATCCCTGTTACAAAATCATGAGCATCCACACAGAT
HQ219596_B CCCCGCTACAAACATTTTCAGAAATTTTGAACAAACTCAATTCTTATCTCTGTTACAAAATCATTAGCATTCACACAGAT
HQ219583_B CCCCGCTACAAACATTTTCAGAAATTTTGAACAAACTCAATTCTTATCTCTGTTACAAAATCATTAGCATTCACACAGAT
HQ219589_B CCCCGCTACAAACATTTTCAGAAATTTTGAACAAACTCAATTCTTATCTCTGTTATAAAATCATTAGCATTCACACAGAT
HQ219600_B TCCCGCTACAAACATTTTCAGAAATTTTTAATAAACTCAATTCTCATCCCTGTTACAAAATCATTAGCATCCACACAGAT
HQ219591_B CCCCGCTACAAACATTTTCAGAAATTTTGAATAAACTCAATTCTCATTCCCGTTACAAAATCATTAGCATTCACACAGAT
HQ219578_B TCCCGCTACAAACATTTTCAGAAATTTTGAACAAACTCAATTCTCATCCCTGTTC-AAAATCATTAGCAATCACACAGAT
HQ219607_B CCC-GCTACAAACATTTTCAGAAATTTTGAACAAACTCAATTCTCATCTCTGTTACAAAATCATTAGCATTCACACAGAT

HT          GACA
HQ219587_A GACA
HQ219590_A GACA
HQ219582_A GACA
HQ219584_A ATCA
HQ219592_A GACA
HQ219599_A GACA
HQ219585_A GACA
HQ219581_B ATCA
HQ219596_B ATCA
HQ219583_B ATCA
HQ219589_B ATCA
HQ219600_B ATCA
HQ219591_B ATCA
HQ219578_B ATCA
HQ219607_B ATCA
```

Patched alignment

```
HQ219644_A TTCTCCGGCTTGCATCATCATCTGGAGAGTAAAGCCGGAGGACTCGGAGCCAGAAGAGGACTGGCTGGCTGTATATTTCA
HQ219645_A TTCTCCGGCTTGCATCATCATCTGGAGAGTAAATCCGGAGGACTCGGAGCCAGAAGAGGACTGGCTGGCTGTATATTTCA
HQ219641_A TTCTCCGGCTTGCATCATCATCTGGAGAGTAAATCCGGAGGACTCGGAGCCAGAAGAGGACTGGCTGGCTGTATATTTCA
HQ219642_A TTCTCCGGCTTGCATCATCATCTGGAGAGTAAAGCCGGAGGACTCGGAGCCAGAAGAGGACTGGCTGGCTGTATATTTCA
HT         TTCTCCGGCTTGCATCATCATCTGGAGAGTAAAGCCGGAGGACTCGGAGCCAGAAGACGACTGGCTGGCTGTATATTTCA
HQ219629_A TTCTCCGGCTTGCATCATCATCTGGAGAGTAAAGCCGGAGGACTCGGAGCCAGAAGAGGACTGGCTGGCTGTATATTTCA
HQ219653_A TTCTCCGGCTTGCATCATCATCTGGAGAGTAAATCCGGAGGACTCGGAGCCAGAAGAGGACTGGCTGGCTGTATATTTCA
HQ219637_A TTCTCCGGCTTGCATCATCATCTGGAGAGTAAATCCGGAGGACTCGGAGCCAGAAGAGGACTGGCTGGCTGTATATTTCA
HQ219630_A TTCTCCGGCTTGCATCATCATCTGGAGAGTAAAGCCGGAGGACTCGGAGCCAGAAGAGGACTGGCTGGCTGTATATTTCA
HQ219649_A TTCTCCGGCTTGCATCATCATCTGGAGAGTAAATCCGGAGGACTCGGAGCCAGAAGAGGACTGGCTGGCTGTATATTTCA
HQ219650_A TTCTCCGGCTTGCATCATCATCTGGAGAGTAAATCCGGAGGACTCGGAGCCAGAAGAGGACTGGCTGGCTGTATATTTCA
HQ219646_A TTCTCCGGCTTGCATCATCATCTGGAGAGTAAATCCGGAGGACTCGGAGCCAGAAGAGGACTGGCTGGCTGTATATTTCA
HQ219651_B TTCTCCGGCTTGCATCATCATCTGGAGCGTAAATCCGGAGGACTCGGACCCGGAAGAGGACTGGCTAGCCGTGTATTTCA
HQ219647_B TTCTCCGGCTTGCATCATCATCTGGAGAGTAAATCCGGAGGACTCAGACCCGGATGAGGACTGGCTAGCCGTGTATTTCA
HQ219635_B TTCGCCGGCTTGCATCATCATCTGGAGAGTAAATCCGGAGGACTCGGACCCGGAAGAGGACTGGCTAGCCGTGTATTTGA
HQ219643_B TTCTCCGGCTTGCATCATCATCTGGAGAGTAAATCCGGAGGACTCAGACCCGGAAGAGGACTGACTAGCCGTGTATTTGA
HQ219631_B TTCTCCGGCTTGCATCATCATCTGGAGCGTAAATCCGGAGGACTCGGACCCGGAGGAGGACTGGCTAGCCGTGTATTTGA
HQ219634_B TTCTCCGGCTTGCATCATCATCTGGAGCGTAAATCCGGAGGACTCGGACCCGGAAGAGGACTGGCTAGCCGTGTATTTGA
HQ219633_B TTCTCCGGCTTGCATCATCATCTGGAGCGTAAATCCGGAGGACTCGGACCCGGATGAGGACTGACTAGCCGTGTATTTGA

HQ219644_A GTTCGTCTTCAAGTTTTCCGCCAGCTGAAAA--AAAACACAGTTTTTGATAAAGAAGTGAAAATAATTTAAATTAGTCTA
HQ219645_A GTTCGTCTTCAAGTTTTCCGCCAGCTGAAAA--AAAACACAGTTTTTGATAAAGAAGTGAAAATAATTGAAATTAGTCTA
HQ219641_A GTTCGTCTTCAAGTTTTCCGCCAGCTGAAAA--AAAACACAGTTTTTGATAAAGAAGTGAAAATAATTGAAATTAGTCTA
HQ219642_A GTTCGTCTTCAAGTTTTCCGCCAGCTGAAAA--AAAACACAGTTTTTGATAAAGAAGTGAAAATAATTGAAATTAGTCTA
HT         GTTCGTCTTCAAGTTTTCCGCCAGCTGAAAAAAAAAACACAGTTTTTGATAAAGAAGTGAAAATAATTTAATTTAGTCTA
HQ219629_A GTTCGTCTTCAAGTTTTCCGCCAGCTGAAAA--AAAACACAGTTTTTGATAAAGAAGTGAAAATAATTTAATTTAGTCTA
HQ219653_A GTTCGTCTTCAAGTTTTCCGCCAGCTGAAAA-AAAAACACAGTTTTTGATAAAGAAGTGAAAATAATTTAAATTAGTCTA
HQ219637_A GTTCGTCTTCAAGTTTTCCGCCAGCTGAAAA-AAAAACACAGTTTTTGATAAAGAAGTGAAAATAATTTAAATTAGTCTA
HQ219630_A GTTCGTCTTCAAGTTTTCCGCCAGCTGAAAA--AAAACACAGTTTTTGATAAAGAAGTGAAAATAATTGAAATTAGTCTA
HQ219649_A GTTCGTCTTCAAGTTTTCCGCCAGCTGAAAA-AAAAACACAGTTTTTGATAAAGAAGTGAAAATAATTTAAATTAGTCTA
HQ219650_A GTTCGTCTTCAAGTTTTCCGCCAGCTGAAAA-AAAAACACAGTTTTTGATAAAGAAGTGAAAATAATTTAAATTAGTCTA
HQ219646_A GTTCGTCTTCAAGTTTTCCGCCAGCTGAAAA-AAAAACACAGTTTTTGATAAAGAAGTGAAAATAATTTAAATTAGTCTA
HQ219651_B GTTCGTCTTCAAGTTTCCCTCCAGCTGAAAA-GAAAACACAGTTTT-GGTTAAAAACTGAAAAAAATTTAAATCAGTTTA
HQ219647_B GTTCGTCTTCAAGTTTCCCTCCAGCTGAAAAAGAAAACACAGTTTTTGATTAGAAACTGAAAAAAATTTAAATCAGTTTA
HQ219635_B GTTCGTCTTCAAGTTTGCCTCCAGCTAAAAAAGAAAACATAGTTTTTGGTTAAGAACTGAAAAAAATTTAAATCAGTTTA
HQ219643_B GTTCGTCTTCAAGTTTTCCTCCAGCTGAAAA-GAAAACACAGTTTTTGATTAGGAACTGAAAAAAAATTAAATCAGTTTA
HQ219631_B GTTCGTCTTCAAGTTTCCCTCCAGCTGAAAAAGAAAACACAGTTTTTGATTAGAAACTGAAAAAAATTTAAATCAGTTTA
HQ219634_B GTTCGTCTTCAAGTTTCCCTCCAGCTGAAAA-GAAAACACAGTTTTTGGTTAAAAACTGAAAAAAATTTAAATCAGTTTA
HQ219633_B GTTCGTCTTCAAGTTTTCCTCCAGCTGAAAA-GAAAACACAGTTTTTGATTAAAAACTGAAACAAATTTAAATCAGTTTA

HQ219644_A TTTTGTCAATTACG-----------TTTATAACTTTTT-AAACTATAGTTGTTTCAAACTATTACAAACAATTATTTTTC
HQ219645_A TTTTGTCAATTACG-----------TTTATAACTTTTT-AAACTATAGTTGTTTCAAACTATTACAAACAATTATTTTTC
HQ219641_A TTTTGTCAATTACG-----------TTTATAACTTTTT-AAACTATAGTTGTTTCAAACTATTACAAACAATTATTTTTC
HQ219642_A TTTTGTCAATTACG-----------TTTATAACTTTTT-AAACTATAGTTGTTTCAAACTATTACAAACAATTATTTTTC
HT         TTTTGTCAATTACG-----------TTTATAACTTTTT-AAACTATAGTTGTTTCAAACTATTACAAACAATTATTTTTC
HQ219629_A TTTTGTCAATTACG-----------TTTATAACTTTTT-AAACTATAGTTGTTTCAATCTATTACAAACAATTATTTTTC
HQ219653_A TTTTGTCAATTACG-----------TTTATAACTTTTT-AAACTATAGTTGTTTCAAACTATTACAAACAATTATTTTTC
HQ219637_A TTTTGTCAATTACG-----------TTTATAACTTTTT-AAACTATAGTTGTTTCAATCTATTACAAACAATTATTTTTC
HQ219630_A TTTTGTCAATTACG-----------TTTATAACTTTTT-AAACTATAGTTGTTTCAAACTATTACAAACAATTATTTTTC
HQ219649_A TTTTGTCAATTACG-----------TTTATAACTTTTT-AAACTATAGTTGTTTCAATCTATTACAAACAATTATTTTTC
HQ219650_A TTTTGTCAATTACG-----------TTTATAACTTTTT-AAACTATAGTTGTTTCAATCTATTACAAACAATTATTTTTC
HQ219646_A TTTTGTCAATTACG-----------TTTATAACTTTTT-AAACTATAGTTGTTTCAATCTATTACAAACAATTATTTTTC
HQ219651_B TTTGGTCAACCACAATACGCCATATTTTATAAGGTTTTCAAACTACAGATTTTT-AAACTATTACAAACAAATACTTTTC
HQ219647_B TTTGGTCAACCACAATACGCCATATTTTATAAGGTTTTTAAACTACAGATTTTT-AAACTATTACAAACAGTTACTTTTC
HQ219635_B TTTGGTCAACCACAATACGCCATATTTTATAAGGTTTTCAAACTACAGATTTTT-AAACTATAACAAACAGTTACTTTTC
HQ219643_B TTTGGTCCACCACAATACGCCATATTTTATAAGGTTTTCAAACTACAGATTTTT-AAACTATTACAAACAGTTACTTTTC
HQ219631_B TTTGGTCAACCACAATACGCCATATTTTATAAGGTTTTTAAACTACAGATTTTT-AAACTATTACAAACAGTTACTTTTC
HQ219634_B TTTGGACAACCACAATACGCCATATTTTATAAGGTTTTCAAACTACAGATTTTT-AAACTATTACAAACAGTTACTTTTC
HQ219633_B TTTGGTCAACCACAATACGCCATATTTTATAAGGTTTTTAAACTACAGATTTTT-AAACTATTACAAACAGTTACTTTTC

HQ219644_A AAAGAGATAAACAGACATGACGACGCAATGTTATTGTAAACATATTCAGTTACAAGGTGAAA---AAAAGCCAACCGAAC
HQ219645_A AAAGAGATAAACAGACATGACGACGCAATGTTATTGTAAACATATTCAGTTACAAGGTGAAA---AAAAGCCAACCGAAC
HQ219641_A AAAGAGATAAACAGACATGACGACGCAATGTTATTGTAAACATATTCAGTTACAAGGTGAAA---AAAAGCCAACCGAAC
HQ219642_A AAAGAGATAAACAGACATGACGACGCAATGTTATTGTAAACATATTCAGTTACAAGGTGAAA---AAAAGCCAACCGAAC
HT         AAAGAGGTAAACAGACATGACGACGCAATGTTATTGTAAACATATTCAGTTACAAGGTGAAA---AAAAGCCAACCGAAC
HQ219629_A AAAGAGGTAAACAGACATGACGACGCAATGTTATTGTAAACATATTCAGTTACAAGGTGAAA---AAAAGCCAACCGAAC
HQ219653_A AAAGAGATAAACAGACATGACGACGCAATGTTATTGTAAACATATTCAGTTACAAGGTGAAA---AAAAGCCAACCGAAC
HQ219637_A AAAGAGGTAAACAGACATGACGACGCAATGTTATTGTAAACATATTCAGTTACAAGGTGAAA---CAAAGCCAACCGAAC
HQ219630_A AAAGAGATAAACAGACATGACGACGCAATGTTATTGTAAACATATTCAGTTACAAGGTGAAA---AAAAGCCAACCGAAC
HQ219649_A AAAGAGGTAAACAGGCATGACGACGCAATGTTATTGTAAACATATTCAGTTACAAGGTGAAA---CAAAGCCAACCGAAC
HQ219650_A AAAGAGGTAAACAGACATGACGACGCAATGTTATTGTAAACATATTCAGTTACAAGGTGAAA---CAAAGCCAACCGAAC
HQ219646_A AAAGAGGTAAACAGACATGACGACGCAATGTTATTGTAAACATATTCAGTTACAAGGTGAAA---CAAAGCCAACCGAAC
HQ219651_B AAA-AGGTAAACAGACATAATGAGGCAATGTTATTGTAAATATATTTAGTTTCAAGGTGAGAA--AAAAGTCAACCGAAC
HQ219647_B AAA-AGGTAAACAGACATAATGAGGCAATGTTATTGTAAACATATTTAGTCTCAAGGTGAGAAA-AAAAGTCAACCGAAC
HQ219635_B AAA-AGGTAAACAGACATAATGAGGCAATGTTATTGTAAACATATTTAGTTTCAAGGTGAGAAA-AAAAGTCAACCGAAC
HQ219643_B AAA-AGGTAAACAGACATAATGAGGCAATGTTATTGTAAACATATTTAGTTTCAAGGTGAAAAAAAAAAGTCAACCGAAC
HQ219631_B AAA-AGATAAACAGACATAACGAGGCAATGTTATTGTAAACATATTTAGTTTCAAGGTGAGAAA-AAAAGTCAACCGAAC
HQ219634_B AAA-AGGTAAGCAGACATAACGAGTCAATGTTATTGTAAACATATTTAGTTTCAAGGTGAGAAA-AAAAGTCAACCGAAC
HQ219633_B AAA-AGGTAAACAGACATAATGAGGCAATGTTATTGTAAACATATTTAGTTTCAAGGTGAGAAA-AAAAGTCAACCGAAC

HQ219644_A ACATATCAAGATGAGTAAATATACAGTGTCACCAAAGCAACTAAAATATTCGGTTTCAAAGCATTTCACACTCACTGGCG
HQ219645_A ACATATCAAGATGAGTAAATATACAGTGTCACCAAAGCAACTAAAATATTCGGTTTCAAAGCATTTCACACTCACTGGCG
HQ219641_A ACATATCAAGATGAGTAAATATACAGTGTCACCAAAGCAACTAAAATATTCGGTTTCAAAGCATTTCACACTCACTGGCG
HQ219642_A ACATATCAAGATGAGTAAATATACAGTGTCACCAAAGCAACTAAAATATTCGGTTTCAAAGCATTTCACACTCACTGGCG
HT         ACATATCAAGATGAGTAAATACACAGTGTCACCAAAGCAACTAAAATATTCGGTTTCAAAGCATTTCACACTCACTGGCG
HQ219629_A ACATATCAAGATGAGTAAATATACAGTGTCACCAAAGCAACTAAAATATTCGGTTTCAAAGCATTTCACACTCACTGGCG
HQ219653_A ACATATCAAGATGAGTAAATATACAGTGTCACCAAAGCAACTAAAATATTCGGTTTCAAAGCATTTCACACTCACTGGCG
HQ219637_A ACATATCAAGATGAGTAAATATACAGTGTCACCAAAGCAACTAAAATATTCGGTTTTAAAGCATTTCACACTCACTGGCG
HQ219630_A ACATATCAAGATGAGTAAATATACAGTGTCACCAAAGCAACTAAAATATTCGGTTTCAAAGCATTTCACACTCACTGGCG
HQ219649_A ACATATCAAGATGAGTAAATATACAGTGTCACCAAAGCAACTAAAATATTCGGTTTTAAAGCATTTCACACTCACTGGCG
HQ219650_A ACATATCAAGATGAGTAAATATACAGTGTCACCAAAGCAACTAAAATATTCGGTTTTAAAGCATTTCACACTCACTGGCG
HQ219646_A ACATATCAAGATGAGTAAATATACAGTGTCACCAAAGCAACTAAAATATTCGGTTTTAAAGCATTTCACACTCACTGGCG
HQ219651_B ACATATCAGTATGTGTAAATATACAGTGTCACAAAAGCAACTAAAATATTCGGTTTCAAAGCATTTCACACTCAGTGTCA
HQ219647_B ACATATCAGTATGTGTAAATATACAGTGTCACCAAAGCAACTAAAATATTCGGTTTCAAAGCATTTCGCACTCAGTGTCA
HQ219635_B ACATATCAGTATGTGTAAATATACAGTGTCACCAAAGCAACTAAAATATTCGGTTTCAAAGCATTTCGCACTCAGTGTCA
HQ219643_B ACATATCAGTATGTGTAAATATACAGCGTCAACAAAGCAACTAAAATATTCGGTTTCAAAGCATTTCACACTCAGTGTCA
HQ219631_B ACATATCAGTATGTGTAAATATACAGCGTCACCAAAGCAACTAAAATATTCGGTTTCAAAGCA-TTCGCACTCAGTGTCA
HQ219634_B ACATATCAGTATGTGTAAATATACAGTGTCACCAAAGCAACTAAAATATTCGGTTTCAAAGCA-TTCACACTCAGTGGCG
HQ219633_B ACATATCAGTATGTGTAAATATACAGTGTCACCAAAGCAACTAAAATATTCGGTTTCAAAGCA-TTCACACTCAGTGGCG
```

```
HQ219644_A ACTTATTGTTATACACACTTTGTGTGGCACTGCCATATATAGCTTGCTACATTTTGTCGAGAAAAATAACCATCCGTATA
HQ219645_A ACTTATTGTTATACACACTTTGTGTGGCACTGCCATATATAGCTTGCTACATTTTGTCGAGAAAAATAACCATCCGTATA
HQ219641_A ACTTATTGTTATACACACTTTGTGTGGCACTGCCATATATAGCTTGCTACATTTTGTCGAGAAAAATAACCATCCGTATA
HQ219642_A ACTTATTGTTATACACACTTTGTGTGGCACTGCCATATATAGCTTGCTACATTTTGTCGAGAAAAATAACCATCCGTATA
HT         ACTTATTGTTATACACACTTTGTGTGGCACTGCCATATATAGCTTGCTACATTTTGTCGAGAAAAATAACCATCCGTATA
HQ219629_A ACTTATTGTTATACACACTTTGTGTGGCACTGCCATATATAGCTTGCTACATTTTGTCGAGAAAAATAACCATCCGTATA
HQ219653_A ACTTATTGTTATACACACTTTGTGTGGCACTGCCATATATAGCTTGCTACATTTTGTCGAGAAAAATAACCATCCGTATA
HQ219637_A ACTTATGGTTATACACACTTTGTGTGGCACTGCCATATATAGCTTGCTACATTTTGTCGAGAAAAATAACCATCCGTATA
HQ219630_A ACTTATTGTTATACACACTTTGTGTGGCACTGCCATATATAGCTTGCTACATTTTGTCGAGAAAAATAACCATCCGTATA
HQ219649_A ACTTATGGTTATACACACTTTGTGTGGCACTGCCATATATAGCTTGCTACATTTTGTCGAGAAAAATAACCATCCGTATA
HQ219650_A ACTTATGGTTATACACACTTTGTGTGGCACTGCCATATATAGCTTGCTACATTTTGTCGAGAAAAATAACCATCCGTATA
HQ219646_A ACTTATGGTTATACACACTTTGTGTGGCACTGCCATATATAGCTTGCTACATTTTGTCGAGAAAAATAACCATCCGTATA
HQ219651_B ACTTATTGTTATACACACTTTGTGTGGTACAACCATATATAGCTTGCAACATTTTGTGGAGGAAAATAACTTTCTGTATA
HQ219647_B ACTTATTGTTATACACACTTTGTGTGGTACAACCATATATAGCTTGCAACATTTTGTGGAGGAAAATAACCGTCTGTATA
HQ219635_B ACTTATTGTTATACACACTTTGTGTGGTACTGCCATATATAGCTTGCTACATTTAGTAGAGGAAAATAACCGTCTGTATA
HQ219643_B ACTTATTGTTATACAAACTTTGTGTGGTACTGCCATATATAGCTTGCTACATTTGGCAGAGGAAAATAACCGTCTGTATA
HQ219631_B ACTTATTGTTATACACACTTTGTGTGGTACTGCCATATATAGCTTGTAACATTTTTGGGAAGAAAATAACTTTCTGTATA
HQ219634_B ACTTATTGTTGTACAAACTTTGTGTGGTACTGCCATATATAGCTTGTAACATTTTGGGGAAGAAAATAACCGTCTGTATA
HQ219633_B ACTTATTGTTATACACACTTTGTGTGGTACTGCCATATATAGCTTGTAACATTTTTGGGAAGAAAATAACCGTCTGTATA

HQ219644_A TCAGATATGATGTTTTAGATAATGCATCACGAGCAACAGGATGTAGATATG-TAGAA-GTGTCACTCATGTAAATGGATA
HQ219645_A TCAGATATGATGTTTTAGATAATGCATCACGAGCAACAGGATGTAGATATG-TAGAA-GTGTCACTCATGTAAATGGATA
HQ219641_A TCAGATATGATGTTTTAGATAATGCATCACGAGCAACAGGATGTAGATATG-TAGAA-GTGTCACTCATGTAAATGGATA
HQ219642_A TCAGATATGATGTTTTAGATAATGCATCACGAGCAACAGGATGTAGATATG-TAGAA-GTGTCACTCATGTAAATGGATA
HT         TCAGATATGATGTCTTAGATAATGCATCACAAGCAACAGGGTGTAGATATG-TAGAA-GTGTCACTCATGTAAATGGATA
HQ219629_A TCAGATATGATGTCTTAGATAATACATCACAAGCAACC---TGTAGATATG-TAGAA-GTGTCACTCATGTAAATGGATA
HQ219653_A TCAGATATGATGTCTTAGATAATGCATCACAAGCAACAGGATGTAGATATG-TAGAA-GTGTCACTCATGTAAATGGATA
HQ219637_A TCAGATATGATGTCTTAGATAATACATCACAAGCAACAGGATGTAGATATG-TAGAAAGTGTCACTCATGTAAATGGATA
HQ219630_A TCAGATATGATGTCTTAGATAATACATCACAAGCAACAGGATGTAGATATG-TAGAA-GTGTCACTCATGTAAATGGATA
HQ219649_A TCAGATATGATGTCTTAGATAATACATCACAAGCAACAGGATGTAGATATG-TAGAA-GTGTCACTCATGTAAATGGATA
HQ219650_A TCAGATATGATGTCTTAGATAATACATCACAAGCAACAGGATGTAGATATG-TAGAA-GTGTCACTCATGTAAATGGATA
HQ219646_A TCAGATATGATGTCTTAGATAATACATCACAAGCAACAGGATGTAGATATG-TAGAA-GTGTCACTCATGTAAATGGATA
HQ219651_B TCAGATATGATGTCTTAGATAATGCATCACAAGCAACAGGATGTAGATATG-TAGAA-GTGTCACTCATGTAAATGGATA
HQ219647_B TCAGATATGATGTCTTAGATAATGCATCACAAGCAACAGGATGTAGATATG-TAGAA-GTGTCACTCATGTAAATGGATA
HQ219635_B TCAGATATGATGCCTTAGATAATGCATCACAAGCAACAGGATGTAGATATG-TAGAA-GTGTCACTCATGTAAATTGATA
HQ219643_B TCAGATATGATGTCTTAGATAATGCATCACAAGCAACAGGATGTAGATATG-TAGGA-GTGTCACTCATGTAAATGGATA
HQ219631_B TCAGATATGATGTCTTAGATAATGCATCACAAGCAACAGGATGTAGATATG-TAGAT-GTGTCACTCATGTAAATGGATA
HQ219634_B TCAGATATGATGTCTTAGATAATGCATCACAAGCAACAGGATGTAGATATG-TAGAA-GTGTCACTCATGTAAATGGATA
HQ219633_B TCAGATATGATGTCTTAGATAATGCATCACAAGCAACAGGATGTAGATATG-TAGAA-GTGTCACTCATGTAAATGGATA

HQ219644_A CGAGTGTCGGTTAAAGGGCTGGGTGGCCTTACAAGCAACATCTGACTT-------TAACAATGTTTCAT-GGCTACCTAC
HQ219645_A CGAGTGTCGGTTAAAGGGCTGGGTGGCCTTACAAGCAACATCTGACTT-------TAACAATGTTTCAT-GGCTACCTAC
HQ219641_A CGAGTGTCGGTTAAAGGGCTGGGTGGCCTTACAAGCAACATCTGACTT-------TAACAATGTTTCAT-GGCTACCTAC
HQ219642_A CGAGTGTCGGTTAAAGGGCTGGGTGGCCTTACAAGCAACATCTGACTT-------TAACAATGTTTCAT-GGCTACCTAC
HT         CGAATGTCGGTTAAAGGGCTGGGTGGCCTTACAAGCAACATGTGACTTAGCATGTTAACAATGTTTCAT-GGCTACCTAC
HQ219629_A CGAGTGTCGGTTAAAGGGCTGGGTGGCCTTACAAGCAACATGTGACTTAGCATGTTAACAATGTTTCAT-GGCTACCTAC
HQ219653_A CGAGTGTCGGTTAAAGGGCTGGGTGGCCTTACAAGCAACATGTGACTTAGCATGTTAACAATGTTTCAT-GGCTACCTAC
HQ219637_A CGAGTGTCGGTTAAAGGGCTGGGTGGCCTTACAAGCAACATGTGACTTAGCATGTTAACAATGTTTCATAGGCTACCTAC
HQ219630_A CGAGTGTCGGTTAAAGGGCTGGGTGGCCTTACAAGCAACATGTGACTTAGCATGTTAACAATGTTTCAT-GGCTACCTAC
HQ219649_A CGAGTGTCGGTTAAAGGGCTGGGTGGCCTTACAAGCAACATGTGACTTAGCATGTTAACAATGTTTCAT-GGCTACCTAC
HQ219650_A CGAGTGTCGGTTAAAGGGCTGGGTGGCCTTACAAGCAACATGTGACTTAGCATGTTAACAATGTTTCAT-GGCTACCTAC
HQ219646_A CGAGTGTCGGTTAAAGGGCTGGGTGGCCTTACAAGCAACATGTGACTTAGCATGTTAACAATGTTTCAT-GGCTACCTAC
HQ219651_B CGAGTGTCGGTTAAAGGGCTGGGTGGCCTTACAAGTAACATGTGACTTAGCATGTTAACAATGTTTCAT-GGCTACCTAC
HQ219647_B TGAGTGTCGGTTAAAGGGTTGGGTGGCCTTACAAGTAACATGTGACTTAGCATGTTAACAATGTTTCAT-GGCTACCTAC
HQ219635_B TGAGTGTCGGTTAAAGGGCTGGGTGGCCTTACAAGTAACATGTGACTTAGCATGTTAACAATGTTTCAT-GGCTACATAC
HQ219643_B TGAGTGTCGGTTAAAGGGCTGGGTGGCCTTACCAGTAACATGTGACTTAGCATGTTAACAATGTTTCAT-GGCTACATAC
HQ219631_B TGAGTGTCGGTTAAAGGGCTGGGTGGCCTTACAAGTAACATGTGACTTAGCATGTTAACAATGTTTCAT-GGCTACCTAC
HQ219634_B TGAGTGTCGGTTAAAGGGCTGGGTGGCCTTACAAGTAACATGTGACTTAGCATGTTAACAATGTTTCAT-GGCTACCTAC
HQ219633_B CGAGTGTCGGTTAAAGGGCTGGGTGGCCTTACAAGTAACATGTGACTTAGCATGTTAACAATGTTTCAT-GGCTACATAC

HQ219644_A AATGCTAGGATGCCCGTGGCAAAACATAAGCCTGTTTGATATTCTCACGACATTTATTTCACGGGTGCTTTGTATTTTCC
HQ219645_A AATGCTAGGATGCCCGTGGCAAAACATAAGCCTGTTTGATATTCTCACGACATTTATTTCACGGGTGCTTTGTATTTTCC
HQ219641_A AATGCTAGGATGCCCGTGGCAAAACATAAGCCTGTTTGATATTCTCACGACATTTATTTCACGGGTGCTTTGTATTTTCC
HQ219642_A AATGCTAGGATGCCCGTGGCAAAACATAAGCCTGTTTGATATTCTCACGACATTTATTTCACGGGTGCTTTGTATTTTCC
HT         AATGCTAGGATGCCCGTGGCAAAACATAAGCCTGTTTGATATTCTCACGACATTTATTTCACGGGTGCTTTGTATTTTCC
HQ219629_A AATGCTAGGATGCCCGTGGCAAAACATAAGCCTGTTTGATATTCTCACGACATTTATTTCACGGGTGCTTTGTATTTTCC
HQ219653_A AATGCTAGGATGCCCGTGGCAAAACATAAGCCTGTTTGATATTCTCACGACATTTATTTCACGGGTGCTTTGTATTTTCC
HQ219637_A AATGCTAGGATGCCCGTGGCAAAACATACACCTGTTTGATATTCTCACGACATTTATTTCACGGGTGCTTTGTATTTTCC
HQ219630_A AATGCTAGGATGCCCGTGGCAAAACATAAGCCTGTTTGATATTCTCACGACATTTATTTCACGGGTGCTTTGTATTTTCC
HQ219649_A AATGCTAGGATGCCCGTGGCAAAACATACACCTGTTTGATATTCTCACGACATTTATTTCACGGGTGCTTTGTATTTTCC
HQ219650_A GATGCTAGGATGCCCGTGGCAAAACATACACCTGTTTGATATTCTCACGACATTTATTTCACGGGTGCTTTGTATTTTCC
HQ219646_A AATGCTAGGATGCCCGTGGCAAAACATACACCTGTTTGATATTCTCACGACATTTATTTCACGGGTGCTTTGTATTTTCC
HQ219651_B AATGCTAGGGTGACCGTGGCAAAACAAAAGGCTGTTTGATATTCTCACGACATTTATTTCACGGGTGCTTTGTATTTTCC
HQ219647_B AATGCTAGGGTGACCGTGGCAAAACAAAGGGATGTTTGATATTCTCACGACATTTATTTCACGGGTGCTTTGTATTTTCC
HQ219635_B AACGCTAGGGTGACCGTGGCAAAACAAAGGGCTGTTTGATATTCTCACGACATTTATTTCACGGGTCCCTTGTATTTTCC
HQ219643_B ATTGCTAGGGTGATCGTGGCAAAACAAAGGGCTGTTTGATATTCTCACGACATTTATTTCACGGGTGCTTTGTATTTTCC
HQ219631_B AATGCTAGGGTGACCGTGGCAAAACAAAGGGCTGTTTGATATTCTCACGACATTTATTTCACGGGTGCTTTGTATTTTCC
HQ219634_B AATGCTAGGGTGACCGTGGCAAAACAAAAGGCTGTTTTATATTCTCACGACATTTATTTCACGGGTGCTTTGTATTTTCC
HQ219633_B AATGCTAGGGTGACCGTGGCAAAACAAAGGGCTGTTTTATATTCTCACGACATTTATTTCACGGGTGCTTTGTATTTTCC

HQ219644_A CACCGACGCCGGGTTGAAATTATAATATATAAAGGTCCTACTGGGTGGCAAGGGGTGTGAGGTCGTTTATAGTTTTGAAA
HQ219645_A CACCGACGCCGGGTTGAAATTATAATATATAAAGGTCCTACTGGGTGGCAAGGGGTGTGAGGTCGTTTATAGTTTTGAAA
HQ219641_A CACCGACGCCGGGTTGAAATTATAATATATAAAGGTCCTACTGGGTGGCAAGGGGTGTGAGGTCGTTTATAGTTTTGAAA
HQ219642_A CACCGACGCCGGGTTGAAATTATAATATATAAAGGTCCTACTGGGTGGCAAGGGGTGTGAGGTCGTTTATAGTTTTGAAA
HT         CACCGACGCCGGGTTGAAATTATAATATATAAAGGTCCTACTGGGTGGCAAGGGGTGTGAGGTCATTTATAGTTTTGAAA
HQ219629_A CACCGACGCCGGGTTGAAATTATAATATATAAAGGTCCTACTGGGTGGCAAGGGGTGTGAGGTCATTTATAGTTTTGAAA
HQ219653_A CACCGACGCCGGGTTGAAATTATAATATATAAAGGTCCTACTGGGTGGCAAGGGGTGTGAGGTCGTTTATAGTTTTGAAA
HQ219637_A CACCGACGCCGGGTTGAAATTATAATATATAAAGGTCCTACTGGGTGGCAAGGGGTGTGAGGTCATTTATAGTTTTGAAA
HQ219630_A CACTGACGCCGGGTTGAAATTATAATATATAAAGGTCCTACTGGGTGGCAAGGGGTGTGAGGTCATTTATAGTTTTGAAA
HQ219649_A CACCGACGCCGGGTTGAAATTATAATATATAAAGGTCCTACTGGGTGGCAAGGGGTGTGAGGTCATTTATAGTTTTGAAA
HQ219650_A CACCGACGCCGGGTTGAAATTATAATATATAAAGGTCCTACTGGGTGGCAAGGGGTGTGAGGTCATTTATAGTTTTGAAA
HQ219646_A CACCGACGCCGGGTTGAAATTATAATATATAAAGGTCCTACTGGGTGGCAAGGGGTGTGAGGTCATTTATAGTTTTGAAA
HQ219651_B CACCGACGCCGGGTTGAAATTATATAATATAAAGGTCCTACTGGGTGGTAAGGGGTATTGGGTCATTTATAGTTTTGAAA
HQ219647_B CACCGACCCCGGGTTGAAATAATATAATATAAAGGTCCTACTGGGTGGCAAGGGGTGTGGGGTCATTTATAGTTTTGAAA
HQ219635_B CACCGACGCCGGGTTGAAATTATATAATATAAAGGTCCTACTGGGTGGCAAGGGGTATGGGGTCATTTATAGTTTTGAAA
HQ219643_B CACCGACCCCGGGTTGAAATTATAATATATAAAGGTCCTACTGGGTGGCAAGGGGTGTGAGGTCATTTATAGTTTTGAAA
HQ219631_B CACCGACCCCGGGTTGAAATTATATAATATAAAGGTCCTACTGGGTGGCAGGGGGTGTGAGGTCATTTATAGTTTTGAAA
HQ219634_B CACCGACCCCGGGTTGAAATTATATTATATAAAGGTCCTACTGGGTGGTAAGGGGTGTGAGGTCATTTATAGTTTTGAAA
HQ219633_B CACCGACGCCGGGTTGAAATTATATAATATAAAGGTCCTACTGGGTGGCAAGGGGTGCGAGGTCATTTATAGTTTTGAAA
```

```
HQ219644_A TCATGATATCCTGAGCCC-TTGAAAGAAGTTTACAAAATAATTTTGATCACTTGTACTTGGA
HQ219645_A TCATGATATCCTGAGCCC-TTGAAAGAAGTTTACAAAATAATTTTGATCACTTGTACTTGGA
HQ219641_A TCATGATATCCTGAGCCC-TTGAAAGAAGTTTACAAAATAATTTTGATCACTTGTACTTGGA
HQ219642_A TCATGATATCCTGAGCCC-TTGAAAGAAGTTTACAAAATAATTTTGATCACTTGTACTTGGA
HT       _ TCATGATATCCTGAGCCC-TTGAAAGAAGTTTACAAAATAATTTCGATCACTTGTACCTGGA
HQ219629_A TCATGATATCCTGAGCCC-TTGAAAGAAGTTTACAAAATAATTTCGATCACTTGTACCTGGA
HQ219653_A TCATGATATCCTGAGCCC-TTGAAAGAAGTTTACAAAATAATTTTGATCACTTGTACTTGGA
HQ219637_A TCATGATATCCTGAGCCC-TTGAAAGAAGTTTACAAAATAATTTCGATCACTTGTACCTGGA
HQ219630_A TCATGATATCCTGAGCCC-TTGAAAGAAGTTTACAAAATAATTTCGATCACTTGTACCTGGA
HQ219649_A TCATGATATCCTGAGCCC-TTGAAAGAAGTTTACAAAATAATTTCGATCACTTGTACCTGGA
HQ219650_A TCATGATATCCTGAGCCC-TTGAAAGAAGTTTACAAAATAATTTCGATCACTTGTACCTGGA
HQ219646_A TCATGATATCCTGAGCCC-TTGAAAGAAGTTTACAAAATAATTTCGATCACTTGTACCTGGA
HQ219651_B TCATGATATCTCGGGCCC-TTGAAACAAGTTTACAAAATAATTTCGATCACTTGTACCTGAA
HQ219647_B TCACGATATCTCAGGCCC-TTGAAACAAGTTTACAAAATAATTTCGATGACTTGTACCTGAA
HQ219635_B TCATGATATCTCGGGCCC-TTGAAACAAGTTTACAAAATAATTTCGATCACTTGTACCTGAA
HQ219643_B TCATGATATCCCGGGCCC-TTGAAACAAGTTTACAAAATAATTTCGATCACTTGTACCTGAA
HQ219631_B TCATGATATCTCAGGCCCCTTGAA-CAAGGTTACAAAATATTTTCGATCACT-GTACCTGAA
HQ219634_B TCATGATATCCCGGGCCC-TTGAAACAAGTTTACAAAATAATTTCGATCACTTGTACCTGAA
HQ219633_B TCATGATATCTCGGGCCC-TTGAAACAAGTTTACAAAATAATTTCGATCACTTGTACCTGAA
```

Vachtp alignment

```
HQ219486_B TTCATCATGGACGTTTGTAGACAGTACGGTAACATCGTGGCCACCTTTGCTTATAGTTGCATTAATAAACTTAGAGATTT
HQ219493_B TTCATCATGGACGTTTGTAGACAGTACGGTAACATCGTGGCCACCTTTGCTTATAGTTGCATTAATAAGCTTAGAGATTT
HQ219484_B TTCATCATGGACGTTTGTAGACAGTACGGTAACATCGTGGCCACCTTTGCTTATAGTTGCATTAATAAACTTAGAGATTT
HQ219485_A TTCATCATGGACGTTTGTAGACAGTACGGTAACATCGTGGCCACCTTTGCTTATAGTTGCATTAATAAACTTAGAGATTT
HQ219487_B TTCATCATGGACGTTTGTAGACAGTACGGTAACATCGTGGCCACCTTTGCTTATAGTTGCATTAATAAACTTAGAGATTT
HQ219501_B TTCATCATGGACGTTTGTAGACAGTACGGTAACATCGTGGCCACCTTTGCTTATAGTTGCATTAATAAACTTAGAGATTT
HQ219497_B TTCATCATGGACGTTTGTAGACAGTACGGTAACATCGTGGCCACCTTTGCTTATAGTTGCATTAATAAACTTAGAGATTT
HQ219483_B TTCATCATGGACGTTTGTAGACAGTACGGTAACATCGTGGCCACCTTTGCTTATAGTTGCATTAATAAACTTAGAGATTT
HQ219482_B TTCATCATGGACGTTTGTAGACAGTACGGTAACATCGTGGCCACCTTTGCTTATAGTTGCATTAATAAACTTAGAGATTT
HQ219490_B TTCATCATGGACGTTTGTAGACAGTACGGTAACATCGTGGCCACCTTTGCTTATAGTTGCATTAATAAACTTAGAGATTT
HQ219479_A TTCATCATGGACGTTTGTAGACAGTACGGTAACATCGTGGCCACCTTAGCTCATGGTTGCATTAATAAACTTAGAGATTT
HQ219504_A TTCATCATGGACGTTTGTAGACAGTACGGTAACATCGTGGCCACCTTAGCTCATGGTTGCATTAATAAACTTAGAGATTT
HQ219500_A TTCATCATGGACGTTTGTAGACAGTACGGTAACATCGTGGCCACCTTAGCTCATGGTTGCATTAATAAACTTAGAGATTT
HQ219499_A TTCATCATGGACGTTTGTAGACAGTACGGTAACATCGTGGCCACCTTAGCTCATGGTTGCATTAATAAACTTAGAGATTT
HQ219498_A TTCATCATGGACGTTTGTAGACAGTACGGTAACATCGTGGCCACCTTAGCTCATGGTTGCATTAATAAACTTAGAGATTT
HQ219496_A TTCATCATGGACGTTTGTAGACAGTACGGTAACATCGTGGCCACCTTAGCTCATGGTTGCATTAATAAACTTAGAGATTT
HQ219494_A TTCATCATGGACGTTTGTAGACAGTACGGTAACATCGTGGCCACCTTAGCTCATGGTTGCATTAATAAACTTAGAGATTT
HQ219492_A TTCATCATGGACGTTTGTAGACAGTACGGTAACATCGTGGCCACCTTAGCTCATGGTTGCATTAATAAACTTAGAGATTT
HQ219491_A TTCATCATGGACGTTTGTAGACAGTACGGTAACATCGTGGCCACCTTAGCTCATGGTTGCATTAATAAACTTAGAGATTT
HQ219480_A TTCATCATGGACGTTTGTAGACAGTACGGTAACATCGTGGCCACCTTAGCTCATGGTTGCATTAATAAACTTAGAGATTT
HQ219495_A TTCATCATGGACGTTTGTAGACAGTACGGTAACATCGTGGCCACCTTAGCTCATGGTTGCATTAATAAACTTAGAGATTT
HT         TTCATCATGGACGTTTGTAGACAGTACGGTAACATCGTGGCCACCTTAGCTCATGGTTGCATTAATAAACTTAGAGATTT
HQ219489_A TTCATCATGGACGTTTGTAGACAGTACGGTAACATCGTGGCCACCTTAGCTCATGGTTGCATTAATAAACTTAGAGATTT
HQ219488_A TTCATCATGGACGTTTGTAGACAGTACGGTAACATCGTGGCCACCTTAGCTCATGGTTGCATTAATAAACTTAGAGATTT

HQ219486_B AACTGCACCTCTACGTGACCGGATCGGAGATGGAAAATCTCAGCGGAAACTCGTGCTTGTGCTCGTTTGTGTAGCACTAC
HQ219493_B AACTGCACCTCTACGTGACCGGATCGGAGATGGAAAATCTCAGCGGAAACTCGTGCTTGTGATCGTTTGTGTAGCACTAC
HQ219484_B AACTGCACCTCTACGTGACCGGATCGGAGATGGAAAATCTCAGCGGAAACTCGTGCTTGTGATCGTTTGTGTAGCACTAC
HQ219485_A AACTGCACCTCTACGTGACCGGATCGGAGATGGAAAATCTCAGCGGAAACTCGTGCTTGTGATCGTTTGTGTAGCACTAC
HQ219487_B AACTGCACCTCTACGTGACCGGATCGGAGATGGAAAATCTCAGCGGAAACTCGTGCTTGTGATCGTTTGTGTAGCACTTC
HQ219501_B AACTGCACCTCTACGTGACCGGATCGGAGATGGAAAATCTCAGCGGAAACTCGTGCTTGTGATCGTTTGTGTAGCACTAC
HQ219497_B AACTGCACCTCTACGTGACCGGATCGGAGATGGAAAATCTCAGCGGAAACTCGTGCTTGTGATCGTTTGTGTAGCACTAC
HQ219483_B AACTGCACCTCTACGTGACCGGATCGGAGATGGAAAATCTCAGCGGAAACTCGTGCTTGTGATCGTTTGTGTAGCACTAC
HQ219482_B AACTGCACCTCTACGTGACCGGATCGGAGATGGAAAATCTCAGCGGAAACTCGTGCTTGTGATCGTTTGTGTAGCACTAC
HQ219490_B AACTGCACCTCTACGTGACCGGATCGGAGATGGAAAATCTCAGCGGAGACTCGTGCTTGTGATCGTTTGTGTAGCACTAC
HQ219479_A AACTGCACCTCTACGTGACCGGATCAGCGATGGAAAATCTCAGCGGAAACTCGTCCTTGTGATCGTTTGTGTAGCACTAC
HQ219504_A AACTGCACCTCTACGTGACCGGATCAGCGATGGAAAATCTCAGCGGAAACTCGTCCTTGTGATCGTTTGTGTAGCACTAC
HQ219500_A AACTGCACCTCTACGTGACCGGATCAGCGATGGAAAATCTCAGCGGAAACTCGTCCTTGTGATCGTTTGTGTAGCACTAC
HQ219499_A AACTGCACCTCTACGTGACCGGATCAGCGATGGAAAATCTCAGCGGAAACTCGTCCTTGTGATCGTTTGTGTAGCACTAC
HQ219498_A AACTGCACCTCTACGTGACCGGATCAGCGATGGAAAATCTCAGCGGAAACTCGTCCTTGTGATCGTTTGTGTAGCACTAC
HQ219496_A AACTGCACCTCTACGTGACCGGATCAGCGATGGAAAATCTCAGCGGAAACTCGTCCTTGTGATCGTTTGTGTAGCACTAC
HQ219494_A AACTGCACCTCTACGTGACCGGATCAGCGATGGAAAATCTCAGCGGAAACTCGTCCTTGTGATCGTTTGTGTAGCACTAC
HQ219492_A AACTGCACCTCTACGTGACCGGATCAGCGATGGAAAATCTCAGCGGAAACTCGTCCTTGTGATCGTTTGTGTAGCACTAC
HQ219491_A AACTGCACCTCTACGTGACCGGATCAGCGATGGAAAATCTCAGCGGAAACTCGTCCTTGTGATCGTTTGTGTAGCACTAC
HQ219480_A AACTGCACCTCTACGTGACCGGATCAGCGATGGAAAATCTCAGCGGAAACTCGTCCTTGTGATCGTTTGTGTAGCACTAC
HQ219495_A AACTGCACCTCTACGTGACCGGATCAGCGATGGAAAATCTCAGCGGAAACTCGTCCTTGTGATCGTTTGTGTAGCACTAC
HT         AACTGCACCTCTACGTGACCGAATCAGCGATGGAAAATCTCAGCGGAAACTCGTCCTTGTGATCGTTTGTGTAGCACTAC
HQ219489_A AACTGCACCTCTACGTGACCGAATCAGCGATGGAAAATCTCAGCGGAAACTCGTCCTTGTGATCGTTTGTGTAGCACTAC
HQ219488_A AACTGCACCTCTACGTGACCGAATCAGCGATGGAAAATCTCAGCGGAAACTCGTCCTTGTGATCGTTTGTGTAGCACTAC

HQ219486_B TGCTTGACAACATGCTGTACATGGTTATTGTTCCAATCATAACCGAATATTTTAATAAAAACGAAACAAAGTCCGCAGTG
HQ219493_B TGCTTGACAACATGCTGTACATGGTTATTGTTCCAATCATAACCGAATATTTCAATAAAAACGTAACAAAGTCCGCAGTG
HQ219484_B TGCTTGACAACATGCTGTACATGGTTATTGTTCCAATCATAACCGAATATTTCAATAAAAACGAAACAAAGTCCGCAGTG
HQ219485_A TGCTTGACAACATGCTGTACATGGTTATTGTTCCAATCATAACCGAATATTTTAATAAAAACGAAACAAAGTCCGCAGTG
HQ219487_B TGCTTGACAACATGCTGTACATGGTTATTGTTCCAATCATAACCGAATATTTCAATAAAAACGAAACAAAGTCCGCAGTG
HQ219501_B TGCTTGACAACATGCTGTACATGGTTATTGTTCCAATCATAACCGAATATTTCAATAAAAACGAAACAAAGTCCGCAGTG
HQ219497_B TGCTTGACAACATGCTGTACATGGTTATTGTTCCAATCATAACCGAATATTTCAATAAAAACGAAACAAAGTCCGCAGTG
HQ219483_B TGCTTGACAACATGCTGTACATGGTTATTGTTCCAATCATAACCGAATATTTCAATAAAAACGAAACAAAGTCCGCAGTG
HQ219482_B TGCTTGACAACATGCTGTACATGGTTATTGTTCCAATCATAACCGAATATTTCAATAAAAACGAAACAAAGTCCGCAGTG
HQ219490_B TGCTTGACAACATGCTGTACATGGTTATTGTTCCAATCATAACCGAATATTTCAATAAAAACGAAACAAAGTCCGCAGTG
HQ219479_A TACTTGACAACATGCTGTACATGGTTATTGTTCCGATCATAACCGAATATTTTAATAAAAACGAAACAAAGTCCGCAGTG
HQ219504_A TACTTGACAACATGCTGTACATGGTTATTGTTCCGATCATAACCGAATATTTTAATAAAAACGAAACAAAGTCCGCAATG
HQ219500_A TACTTGACAACATGCTGTACATGGTTATTGTTCCGATCATAACCGAATATTTTAATAAAAACGAAACAAAGTCCGCAATG
HQ219499_A TACTTGACAACATGCTGTACATGGTTATTGTTCCGATCATAACCGAATATTTTAATAAAAACGAAACAAAGTCCGCAATG
HQ219498_A TACTTGACAACATGCTGTACATGGTTATTGTTCCGATCATAACCGAATATTTTAATAAAAACGAAACAAAGTCCGCAATG
HQ219496_A TACTTGACAACATGCTGTACATGGTTATTGTTCCGATCATAACCGAATATTTTAATAAAAACGAAACAAAGTCCGCAATG
HQ219494_A TACTTGACAACATGCTGTACATGGTTATTGTTCCGATCATAACCGAATATTTTAATAAAAACGAAACAAAGTCCGCAATG
HQ219492_A TACTTGACAACATGCTGTACATGGTTATTGTTCCGATCATAACCGAATATTTTAATAAAAACGAAACAAAGTCCGCAATG
HQ219491_A TACTTGACAACATGCTGTACATGGTTATTGTTCCGATCATAACCGAATATTTTAATAAAAACGAAACAAAGTCCGCAATG
HQ219480_A TACTTGACAACATGCTGTACATGGTTATTGTTCCGATCATAACCGAATATTTTAATAAAAACGAAACAAAGTCCGCAATG
HQ219495_A TACTTGACAACATGCTGTACATGGTTATTGTTCCGATCATAACCGAATATTTTAATAAAAACGAAACAAAGTCCGCAATG
HT         TACTTGACAACATGCTGTACATGGTTATTGTTCCGATCATAACCGAATATTTTAATAAAAACGAAACAAAGTCCGCAGTG
HQ219489_A TACTTGACAACATGCTGTACATGGTTATTGTTCCGATCATAACCGAATATTTTAATAAAAACGAAACAAAGTCCGCAGTG
HQ219488_A TACTTGACAACATGCTGTACATGGTTATTGTTCCGATCATAACCGAATATTTTAATAAAAACGAAACAAAGTCCGCAGTG

HQ219486_B CTTTCAACAACATCGCAAATAATGAATGACTTTATCAGTACCCCATACACCAATAACGTCACA---CCAGCAGCAATTCA
HQ219493_B CTTTCAACAACATCGCAAATAATGAATGACTTTATCAGTACCCCATACACCAATAACGTCACA---CCAGCAGCACTTCA
HQ219484_B CTTTCAACAACATCGCAAATAATGAATGACTTTATCAGTACCCCATACACCAATAACGTCACA---CCAGCAGCACTTCA
HQ219485_A CTTTCAACAACATCGCAAATAATGAATGACTTTATCAGTACCCCATACACCAATAACGTCACA---CCAGCAGCACTTCA
HQ219487_B CTTTCAACAACATCGCAAATAATGAATGACTTTATCAGTACCCCATACACCAATAACGTCACA---CCAGCAGCACTTCA
HQ219501_B CTTTCAACAACATCGCAAATAATGAATGACTTTATCAGTACCCCATACACCAATAACGTCACA---CCAGCAGCACTTCA
HQ219497_B CTTTCAACAACATCGCAAATAATGAATGACTTTATCAGTACCCCATACACCAATAACGTCACA---CCAGCAGCACTTCA
HQ219483_B CTTTCAACAACATCGCAAATAATGAATGACTTTATCAGTACCCCATACACCAATAACGTCACA---CCAGCAGCACTTCA
HQ219482_B CTTTCAACAACATCGCAAATAATGAATGACTTTATCAGTACCCCATACACCAATAACGTCACA---CCAGCAGCACTTCA
HQ219490_B CTTTCAACAACATCGCAAATAATGAATGACTTTATCAGTACCCCATACACCAATAACGTCACA---CCAGCAGCACTTCA
HQ219479_A CTTTCAACAACATCGCAAATAATGAATGACTTTATCAGTACCCCATACATCAATAACGTCACACCCCCAGCAGCACTCCA
HQ219504_A CTTTCAACAACATCGCAAATAATGAATGACTTTATCAGTACCCCATACATCAATAACGTCACACCCCCAGCAGCACTCCA
HQ219500_A CTTTCAACAACATCGCAAATAATGAATGACTTTATCAGTACCCCATACATCAATAACGTCACACCCCCAGCAGCACTCCA
HQ219499_A CTTTCAACAACATCGCAAATAATGAATGACTTTATCAGTACCCCATACATCAATAACGTCACACCCCCAGCAGCACTCCA
HQ219498_A CTTTCAACAACATCGCAAATAATGAATGACTTTATCAGTACCCCATACATCAATAACGTCACACCCCCAGCAGCACTCCA
HQ219496_A CTTTCAACAACATCGCAAATAATGAATGACTTTATCAGTACCCCATACATCAATAACGTCACACCCCCAGCAGCACTCCA
HQ219494_A CTTTCAACAACATCGCAAATAATGAATGACTTTATCAGTACCCCATACATCAATAACGTCACACCCCCAGCAGCACTCCA
HQ219492_A CTTTCAACAACATCGCAAATAATGAATGACTTTATCAGTACCCCATACATCAATAACGTCACACCCCCAGCAGCACTCCA
HQ219491_A CTTTCAACAACATCGCAAATAATGAATGACTTTATCAGTACCCCATACATCAATAACGTCACACCCCCAGCAGCACTCCA
HQ219480_A CTTTCAACAACATCGCAAATAATGAATGACTTTATCAGTACCCCATACATCAATAACGTCACACCCCCAGCAGCACTCCA
HQ219495_A CTTTCAACAACATCGCAAATAATGAATGACTTTATCAGTACCCCATACATCAATAACGTCACACCCCCAGCAGCACTCCA
HT         CTTTCAACAACATCGCAAATAATGAATGACTTTATCAGTACCCCATACATCAATAACGCCACACCCCCAGCAGCACTCCA
HQ219489_A CTTTCAACAACATCGCAAATAATGAATGACTTTATCAGTACCCCATACATCAATAACGTCACACCCCCAGCAGCACTCCA
HQ219488_A CTTTCAACAACATCGCAAATAATGAATGACTTTATCAGTACCCCATACATCAATAACGTCACACCCCCAGCAGCACTCCA
```

```
HQ219486_B ATCTCATCTACAAACTTCTGGACCTGACGAAGACGGTACTGAAGATACTTTAACCGGAATCCTGTTTGCGTCCAAAGCAA
HQ219493_B ATCTCATCTACAAACTTCTGGACCTGACGAAGACGGTACTGAAGATACTTTAACCGGAATCCTGTTTGCGTCCAAAGCAA
HQ219484_B ATCTCATCTACAAACTTCTGGACCTGACGAAGACGGTACTGAAGATACTTTAACCGGAATCCTGTTTGCGTCCAAAGCAA
HQ219485_A ATCTCATCTACAAACTTCTGGACCTGACGAAGACGGTACTGAAGATACTTTAACCGGAATCCTGTTTGCGTCCAAAGCAA
HQ219487_B ATCTCATCTACAAACTTCTGGACCTGACGAAGACGGTACTGAAGATACTTTAACCGGAATCCTGTTTGCGTCCAAAGCAA
HQ219501_B ATCTCATCTACAAACTTCTGGACCTGACGAAGACGGTACTGAAGATACTTTAACCGGAATCCTGTTTGCGTCCAAAGCAA
HQ219497_B ATCTCATCTACAAACTTCTGGACCTGACGAAGACGGTACTGAAGATACTTTAACCGGAATCCTGTTTGCGTCCAAAGCAA
HQ219483_B ATCTCATCTACAAACTTCTGGACCTGACGAAGACGGTACTGAAGATACTTTAACCGGAATCCTGTTTGCGTCCAAAGCAA
HQ219482_B ATCTCATCTACAAACTTCTGGACCTGACGAAGACGGTACTGAAGATACTTTAACCGGAATCCTGTTTGCGTCCAAAGCAA
HQ219490_B ATCTCATCTACAAACTTCTGGACCTGACGAAGACGGTACTGAAGATACTTTAACCGGAATCCTGTTTGCGTCCAAAGCAA
HQ219479_A ATCTCATCTACAAACTTCTGGCCCTGACAAAGACGGTACTGAAGACACTTTAACCGGAATCCTGTTTGCGTCCAAAGCAA
HQ219504_A ATCTCATCTACAAACTTCTGGCCCTGACAAAGACGGTACTGAAGACACTTTAACCGGAATCCTGTTTGCGTCCAAAGCAA
HQ219500_A ATCTCATCTACAAACTTCTGGCCCTGACAAAGACGGTACTGAAGACACTTTAACCGGAATCCTGTTTGCGTCCAAAGCAA
HQ219499_A ATCTCATCTACAAACTTCTGGCCCTGACAAAGACGGTACTGAAGACACTTTAACCGGAATCCTGTTTGCGTCCAAAGCAA
HQ219498_A ATCTCATCTACAAACTTCTGGCCCTGACAAAGACGGTACTGAAGACACTTTAACCGGAATCCTGTTTGCGTCCAAAGCAA
HQ219496_A ATCTCATCTACAAACTTCTGGCCCTGACAAAGACGGTACTGAAGACACTTTAACCGGAATCCTGTTTGCGTCCAAAGCAA
HQ219494_A ATCTCATCTACAAACTTCTGGCCCTGACAAAGACGGTACTGAAGACACTTTAACCGGAATCCTGTTTGCGTCCAAAGCAA
HQ219492_A ATCTCATCTACAAACTTCTGGCCCTGACAAAGACGGTACTGAAGACACTTTAACCGGAATCCTGTTTGCGTCCAAAGCAA
HQ219491_A ATCTCATCTACAAACTTCTGGCCCTGACAAAGACGGTACTGAAGACACTTTAACCGGAATCCTGTTTGCGTCCAAAGCAA
HQ219480_A ATCTCATCTACAAACTTCTGGCCCTGACAAAGACGGTACTGAAGACACTTTAACCGGAATCCTGTTTGCGTCCAAAGCAA
HQ219495_A ATCTCATCTACAAACTTCTGGCCCTGACAAAGACGGTACTGAAGACACTTTAACCGGAATCCTGTTTGCGTCCAAAGCAA
HT         ATCTCATCTACAAACTTCTGGCCCTGACAAAGACGGTACTGAAGACACTTTAACCGGAATCCTGTTTGCGTCCAAAGCAA
HQ219489_A ATCTCATCTACAAACTTCTGGCCCTGACAAAGACGGTACTGAAGACACTTTAACCGGAATCCTGTTTGCGTCCAAAGCAA
HQ219488_A ATCTCATCTACAAACTTCTGGCCCTGACAAAGACGGTACTGAAGACACTTTAACCGGAATCCTGTTTGCGTCCAAAGCAA

HQ219486_B TCGTGCAATTAATGGCGAATCCGTTTACTGGAACCTTTATAGATCGAGTTGGATACATAAAGCCATTAACTTTGGGGCTG
HQ219493_B TCGTGCAACTAATGGCGAATCCGTTTACTGGAACCTTTATAGATCGAGTTGGATACATAAAGCCATTAACTTTGGGGCTG
HQ219484_B TCGTGCAATTAATGGCGAATCCGTTTACTGGAACCTTTATAGATCGAGTTGGATACATAAAGCCATTAACTTTGGGGCTG
HQ219485_A TCGTGCAATTAATGGCGAATCCGTTTACTGGAACCTTTATAGATCGAGTTGGATACATAAAGCCATTAACTTTGGGGCTG
HQ219487_B TCGTGCAATTAATGGCGAATCCGTTTACTGGAACCTTTATAGATCGAGTTGGATACATAAAGCCATTAACTTTGGGGCTG
HQ219501_B TCGTGCAATTAATGGCGAATCCGTTTACTGGAACCTTTATAGATCGAGTTGGATACATAAAGCCATTAACTTTGGGGCTG
HQ219497_B TCGTGCAATTAATGGCGAATCCGTTTACTGGAACCTTTATAGATCGAGTTGGATACATAAAGCCATTAACTTTGGGGCTG
HQ219483_B TCGTGCAATTAATGGCGAATCCGTTTACTGGAACCTTTATAGATCGAGTTGGATACATAAAGCCATTAACTTTGGGGCTG
HQ219482_B TCGTGCAATTAATGGCGAATCCGTTTACTGGAACCTTTATAGATCGAGTTGGATACATAAAGCCATTAACTTTGGGGCTG
HQ219490_B TCGTGCAATTAATGGCGAATCCGTTTACTGGAACCTTTATAGATCGAGTTGGATACATAAAGCCATTAACTTTGGGGCTG
HQ219479_A TCGTGCAATTAATGGCAAACCCGTTTACTGGAACCTTTATAGATCGAGTTGGATACATAAAGCCATTAACTTTGGGGCTG
HQ219504_A TCGTGCAATTAATGGCAAACCCGTTTACTGGAACCTTTATAGATCGAGTTGGATACATAAAGCCATTAACTTTGGGGCTG
HQ219500_A TCGTGCAATTAATGGCAAACCCGTTTACTGGAACCTTTATAGATCGAGTTGGATACATAAAGCCATTAACTTTGGGGCTG
HQ219499_A TCGTGCAATTAATGGCAAACCCGTTTACTGGAACCTTTATAGATCGAGTTGGATACATAAAGCCATTAACTTTGGGGCTG
HQ219498_A TCGTGCAATTAATGGCAAACCCGTTTACTGGAACCTTTATAGATCGAGTTGGATACATAAAGCCATTAACTTTGGGGCTG
HQ219496_A TCGTGCAATTAATGGCAAACCCGTTTACTGGAACCTTTATAGATCGAGTTGGATACATAAAGCCATTAACTTTGGGGCTG
HQ219494_A TCGTGCAATTAATGGCAAACCCGTTTACTGGAACCTTTATAGATCGAGTTGGATACATAAAGCCATTAACTTTGGGGCTG
HQ219492_A TCGTGCAATTAATGGCAAACCCGTTTACTGGAACCTTTATAGATCGAGTTGGATACATAAAGCCATTAACTTTGGGGCTG
HQ219491_A TCGTGCAATTAATGGCAAACCCGTTTACTGGAACCTTTATAGATCGAGTTGGATACATAAAGCCATTAACTTTGGGGCTG
HQ219480_A TCGTGCAATTAATGGCAAACCCGTTTACTGGAACCTTTATAGATCGAGTTGGATACATAAAGCCATTAACTTTGGGGCTG
HQ219495_A TCGTGCAATTAATGGCAAACCCGTTTACTGGAACCTTTATAGATCGAGTTGGATACATAAAGCCATTAACTTTGGGGCTG
HT         TCGTGCAATTAATGGCAAACCCGTTTACTGGAACCTTTATAGATCGAGTTGGATACATAAAGCCATTAACTTTGGGGCTG
HQ219489_A TCGTGCAATTAATGGCAAACCCGTTTACTGGAACCTTTATAGATCGAGTTGGATACATAAAGCCATTAACTTTGGGGCTG
HQ219488_A TCGTGCAATTAATGGCAAACCCGTTTACTGGAACCTTTATAGATCGAGTCGGATACATAAAGCCATTAACTTTGGGGCTG

HQ219486_B ATGGTTATGTTTTTATCCACAGCGTTGTTTGCTTGTGCAAAGGGCTTTGCTGTATTGTTCTTAGTTAGAAGTTTACAGGG
HQ219493_B ATGGTTATGTTTTTATCCACAGCGTTGTTTGCTTGTGCAAAGGGCTTTGCTGTATTGTTCTTAGCTAGAAGTTTACAGGG
HQ219484_B ATGGTTATGTCTTTATCCACAGCGTTGTTTGCTTGTGCAAAGGGCTTTGCTGTATTGTTCTTAGCTAGAAGTTTACAGGG
HQ219485_A ATGGTTATGTTTTTATCCACAGCGTTGTTTGCTTGTGCAAAGGGCTTTGCTGTATTGTTCTTAGCTAGAAGTTTACAGGG
HQ219487_B ATGGTTATGTTTTTATCCACAGCGTTGTTTGCTTGTGCAAAGGGCTTTGCTGTATTGTTCTTAGCTAGAAGTTTACAGGG
HQ219501_B ATGGTTATGTTTTTATCCACAGCGTTGTTTGCTTGTGCAAAGGGCTTTGCTGTATTGTTCTTAGCTAGAAGTTTACAGGG
HQ219497_B ATGGTTATGTTTTTATCCACAGCGTTGTTTGCTTGTGCAAAGGGCTTTGCTGTATTGTTCTTAGCTAGAAGTTTACAGGG
HQ219483_B ATGGTTATGTTTTTATCCACAGCGTTGTTTGCTTGTGCAAAGGGCTTTGCTGTATTGTTCTTAGCTAGAAGTTTACAGGG
HQ219482_B ATGGTTATGTTTTTATCCACAGCGTTGTTTGCTTGTGCAAAGGGCTTTGCTGTATTGTTCTTAGCTAGAAGTTTACAGGG
HQ219490_B ATGGTTATGTTTTTATCCACAGCGTTGTTTGCTTGTGCAAAGGGCTTTGCTGTATTGTTCTTAGCTAGAAGTTTACAGGG
HQ219479_A ATGGTTATGTTTTTATCCACAGCGTTGTTTGCTTGTGCAGAGGGCTTTGCTGTATTGTTCTTAGCTAGAAGTTTACAGGG
HQ219504_A ATGGTTATGTTTTTATCCACAGCGTTGTTTGCTTGTGCAGAGGGCTTTGCTGTATTGTTCTTAGCTAGAAGTTTACAGGG
HQ219500_A ATGGTTATGTTTTTATCCACAGCGTTGTTTGCTTGTGCAGAGGGCTTTGCTGTATTGTTCTTAGCTAGAAGTTTACAGGG
HQ219499_A ATGGTTATGTTTTTATCCACAGCGTTGTTTGCTTGTGCAGAGGGCTTTGCTGTATTGTTCTTAGCTAGAAGTTTACAGGG
HQ219498_A ATGGTTATGTTTTTATCCACAGCGTTGTTTGCTTGTGCAGAGGGCTTTGCTGTATTGTTCTTAGCTAGAAGTTTACAGGG
HQ219496_A ATGGTTATGTTTTTATCCACAGCGTTGTTTGCTTGTGCAGAGGGCTTTGCTGTATTGTTCTTAGCTAGAAGTTTACAGGG
HQ219494_A ATGGTTATGTTTTTATCCACAGCGTTGTTTGCTTGTGCAGAGGGCTTTGCTGTATTGTTCTTAGCTAGAAGTTTACAGGG
HQ219492_A ATGGTTATGTTTTTATCCACAGCGTTGTTTGCTTGTGCAGAGGGCTTTGCTGTATTGTTCTTAGCTAGAAGTTTACAGGG
HQ219491_A ATGGTTATGTTTTTATCCACAGCGTTGTTTGCTTGTGCAGAGGGCTTTGCTGTATTGTTCTTAGCTAGAAGTTTACAGGG
HQ219480_A ATGGTTATGTTTTTATCCACAGCGTTGTTTGCTTGTGCAGAGGGCTTTGCTGTATTGTTCTTAGCTAGAAGTTTACAGGG
HQ219495_A ATGGTTATGTTTTTATCCACAGCGTTGTTTGCTTGTGCAGAGGGCTTTGCTGTATTGCTCTTAGCTAGAAGTTTACAGGG
HT         ATGGTTATGTTTTTATCCACAGCGTTGTTTGCTTGTGCAGAGGGCTTTGCTGTATTGTTCTTAGCTAGAAGTTTACAGGG
HQ219489_A ATGGTTATGTTTTTATCCACAGCGTTGTTTGCTTGTGCAGAGGGCTTTGCTGTATTGTTCTTAGCTAGAAGTTTACAGGG
HQ219488_A ATGGTTATGTTTTTATCCACAGCGTTGTTTGCTTGTGCAGAGGGCTTTGCTGTATTGTTCTTAGCTAGAAGTTTACAGGG

HQ219486_B TCTCGGTTCCGCACTAGCCGATACAGCATCCCTTGGGCTCATTGCCGATCGTTTCCAAGATGAAGCAGAGAGATCAAAAG
HQ219493_B TCTCGGTTCCGCACTAGCCGATACAGCATCCCTTGGGCTCATTGCCGATCGTTTCCAAGATGAAGCAGAGAGATCAAAAG
HQ219484_B TCTCGGTTCCGCACTAGCCGATACAGCATCCCTTGGGCTCATTGCCGATCGTTTCCAAGATGAAGCAGAGAGATCAAAAG
HQ219485_A TCTCGGTTCCGCACTAGCCGATACAGCATCCCTTGGGCTCATTGCCGATCGTTTCCAAGATGAAGCAGAGAGATCAAAAG
HQ219487_B TCTCGGTTCCGCACTAGCCGATACAGCATCCCTTGGGCTCATTGCCGATCGTTTCCAAGATGAAGCAGAGAGATCAAAAG
HQ219501_B TCTCGGTTCCGCACTAGCCGATACAGCATCCCTTGGGCTCATTGCCGATCGTTTCCAAGATGAAGCAGAGAGATCAAAAG
HQ219497_B TCTCGGTTCCGCACTAGCCGATACAGCATCCCTTGGGCTCATTGCCGATCGTTTCCAAGATGAAGCAGAGAGATCAAAAG
HQ219483_B TCTCGGTTCCGCACTAGCCGATACAGCATCCCTTGGGCTCATTGCCGATCGTTTCCAAGATGAAGCAGAGAGATCAAAAG
HQ219482_B TCTCGGTTCCGCACTAGCCGATACAGCATCCCTTGGGCTCATTGCCGATCGTTTCCAAGATGAAGCAGAGAGATCAAAAG
HQ219490_B TCTCGGTTCCGCACTAGCCGATACAGCATCCCTTGGGCTCATTGCCGATCGTTTCCAAGATGAAGCAGAGAGATCAAAAG
HQ219479_A TCTCGGTTCTGCATTAGCCGATACAGCATCCCTTGGCCTCATTGCCGATCGTTTCCAAGATGAAGCAGAGAGATCAAAAG
HQ219504_A TCTCGGTTCTGCATTAGCCGATACAGCATCCCTTGGCCTCATTGCCGATCGTTTCCAAGATGAAGCAGAGAGATCAAAAG
HQ219500_A TCTCGGTTCTGCATTAGCCGATACAGCATCCCTTGGCCTCATTGCCGATCGTTTCCAAGATGAAGCAGAGAGATCAAAAG
HQ219499_A TCTCGGTTCTGCATTAGCCGATACAGCATCCCTTGGCCTCATTGCCGATCGTTTCCAAGATGAAGCAGAGAGATCAAAAG
HQ219498_A TCTCGGTTCTGCATTAGCCGATACAGCATCCCTTGGCCTCATTGCCGATCGTTTCCAAGATGAAGCAGAGAGATCAAAAG
HQ219496_A TCTCGGTTCTGCATTAGCCGATACAGCATCCCTTGGCCTCATTGCCGATCGTTTCCAAGATGAAGCAGAGAGATCAAAAG
HQ219494_A TCTCGGTTCTGCATTAGCCGATACAGCATCCCTTGGCCTCATTGCCGATCGTTTCCAAGATGAAGCAGAGAGATCAAAAG
HQ219492_A TCTCGGTTCTGCATTAGCCGATACAGCATCCCTTGGCCTCATTGCCGATCGTTTCCAAGATGAAGCAGAGAGATCAAAAG
HQ219491_A TCTCGGTTCTGCATTAGCCGATACAGCATCCCTTGGCCTCATTGCCGATCGTTTCCAAGATGAAGCAGAGAGATCAAAAG
HQ219480_A TCTCGGTTCTGCATTAGCCGATACAGCATCCCTTGGCCTCATTGCCGATCGTTTCCAAGATGAAGCAGAGAGATCAAAAG
HQ219495_A TCTCGGTTCTGCATTAGCCGATACAGCATCCCTTGGCCTCATTGCCGATCGTTTCCAAGATGAAGCAGAGAGATCAAAAG
HT         TCTCGGTTCCGCATTAGCCGATACAGCATCCCTTGGCCTCATTGCCGATCGTTTCCAAGATGAAGCAGAGAGATCAAAAG
HQ219489_A TCTCGGTTCCGCATTAGCCGATACAGCATCCCTTGGCCTCATTGCCGATCGTTTCCAAGATGAAGCAGAGAGATCAAAAG
HQ219488_A TCTCGGTTCCGCATTAGCCGATACAGCATCCCTTGGCCTCATTGCCGATCGTTTCCAAGATGAAGCAGAGAGATCAAAAG
```

```
HQ219486_B CGCTTGGCCTCGCTCTCGCATTTA
HQ219493_B CGCTTGGCCTCGCTCTCGCATTTA
HQ219484_B CGCTTGGCCTCGCTCTCGCATTTA
HQ219485_A CGCTTGGCCTCGCTCTCGCATTTA
HQ219487_B CGCTTGGCCTCGCTCTCGCATTTA
HQ219501_B CGCTTGGCCTCGCTCTCGCATTTA
HQ219497_B CGCTTGGCCTCGCTCTCGCATTTA
HQ219483_B CGCTTGGCCTCGCTCTCGCATTTA
HQ219482_B CGCTTGGCCTCGCTCTCGCATTTA
HQ219490_B CGCTTGGCCTCGCTCTCGCATTTA
HQ219479_A CGCTTGGCCTCGCTCTAGCATTTA
HQ219504_A CGCTTGGCCTCGCTCTAGCATTTA
HQ219500_A CGCTTGGCCTCGCTCTAGCATTTA
HQ219499_A CGCTTGGCCTCGCTCTAGCATTTA
HQ219498_A CGCTTGGCCTCGCTCTAGCATTTA
HQ219496_A CGCTTGGCCTCGCTCTAGCATTTA
HQ219494_A CGCTTGGCCTCGCTCTAGCATTTA
HQ219492_A CGCTTGGCCTCGCTCTAGCATTTA
HQ219491_A CGCTTGGCCTCGCTCTAGCATTTA
HQ219480_A CGCTTGGCCTCGCTCTAGCATTTA
HQ219495_A CGCTTGGCCTCGCTCTAGCATTTA
HT         CGCTTGGCCTCGCTCTAGCATTTA
HQ219489_A CGCTTGGCCTCGCTCTAGCATTTA
HQ219488_A CGCTTGGCCTCGCTCTAGCATTTA
```

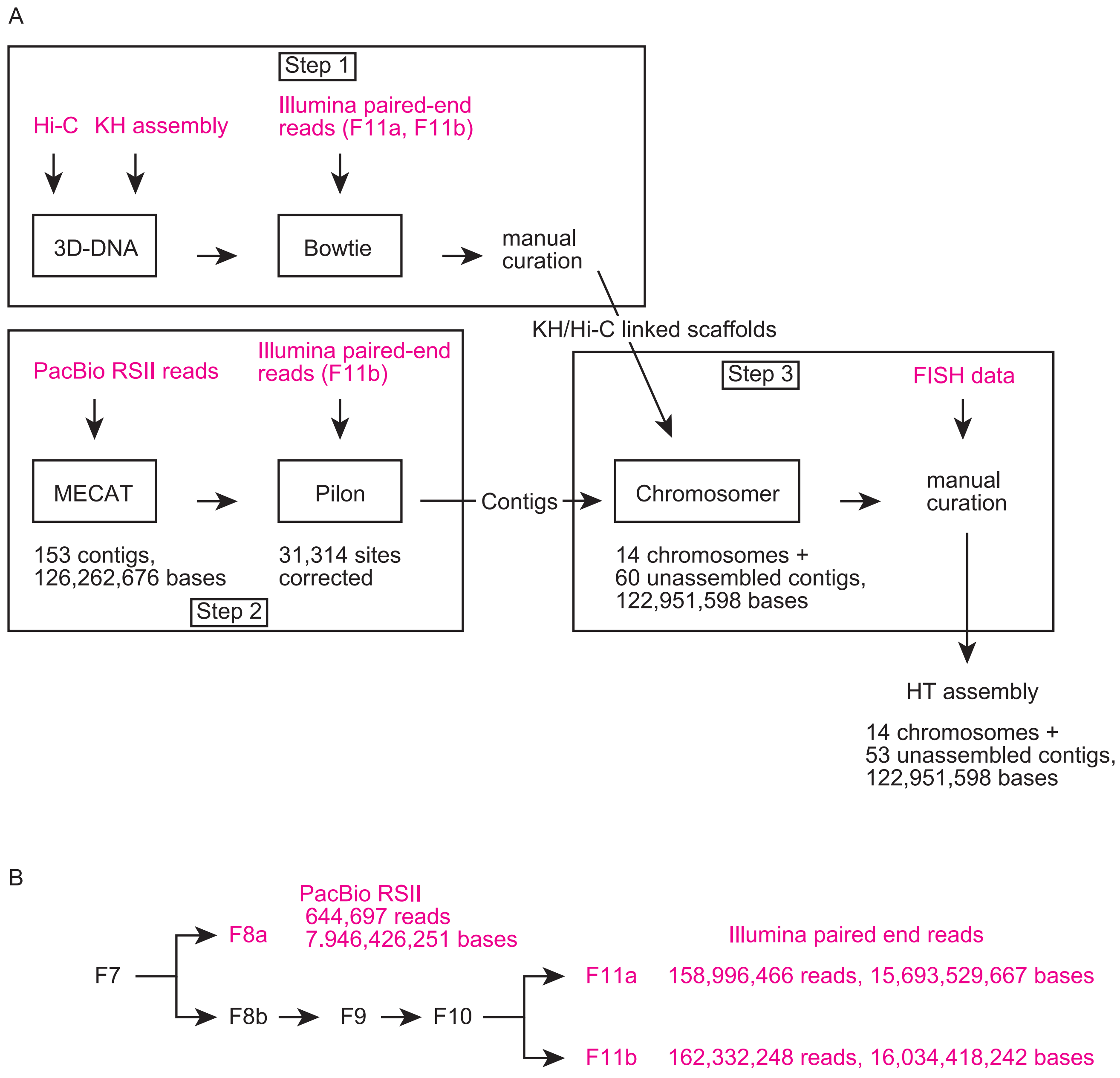

Supplementary figure S2. A detailed outline of genome sequence determination. (A) The detailed assembly strategy in the present study. Data types used in each step are shown in magenta, and computer programs used are enclosed by black lines. (B) Inbred line individuals we used in the present study. For PacBio RSII sequencing, we used genomic DNA of an individual in the 8th generation (F8a). In a previous study (Satou et al. 2015), we obtained Illumina sequencing reads from two individuals in the 11th generation (F11a and F11b). These animals are derived from a single animal in the 7th generation. Numbers of sequencing reads and total nucleotide lengths are shown in magenta.

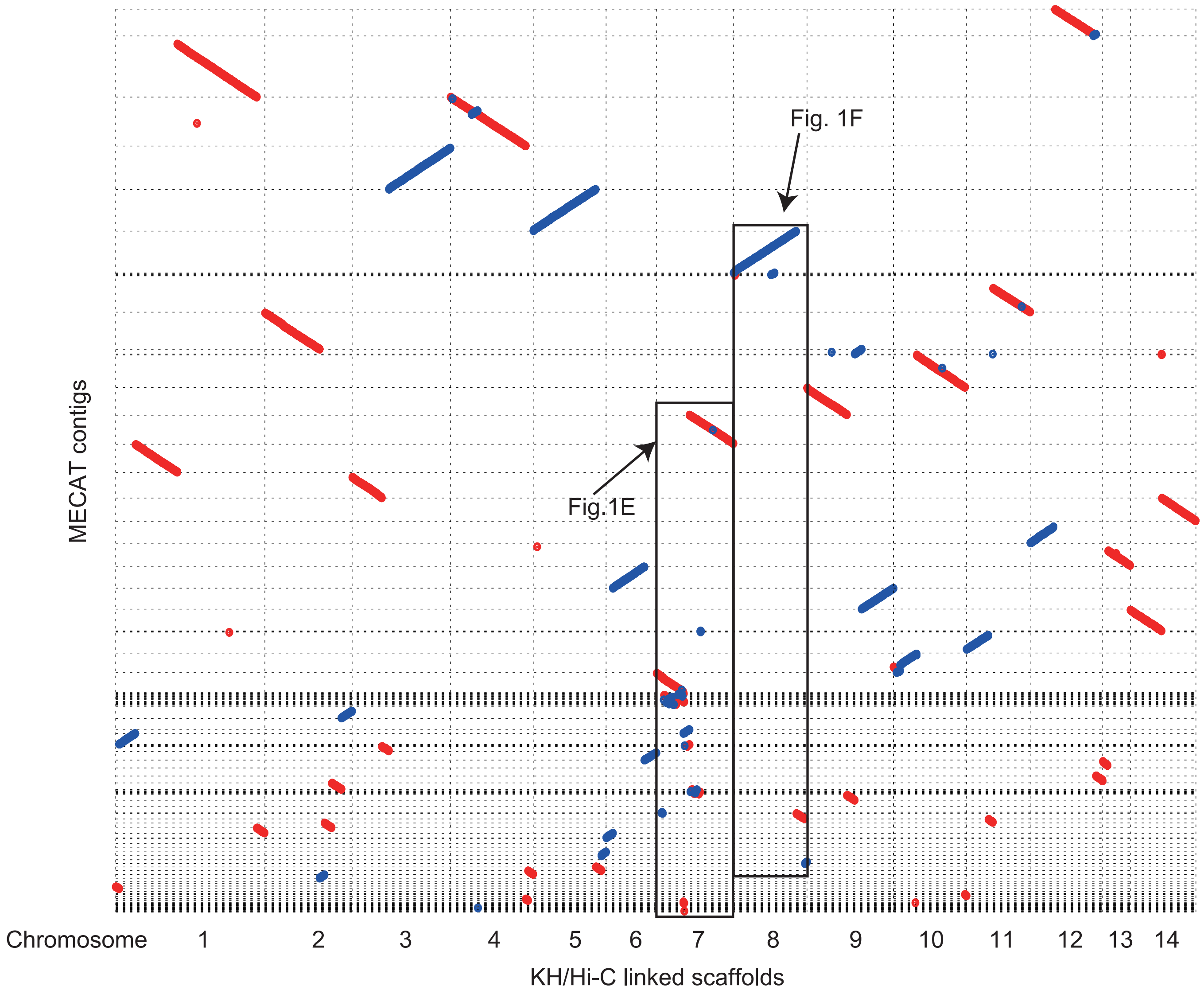

Supplementary figure S3. A comparison of nucleotide sequences between contigs obtained with the MECAT assembler and KH/Hi-C-linked scaffolds. Nucmer (Kurtz et al. 2004) was used for the alignment. Higher magnification views for chromosomes 7 and 8 (boxes) are in Figures 1E and F. Forward alignments are shown in red and reverse alignments are shown in blue.

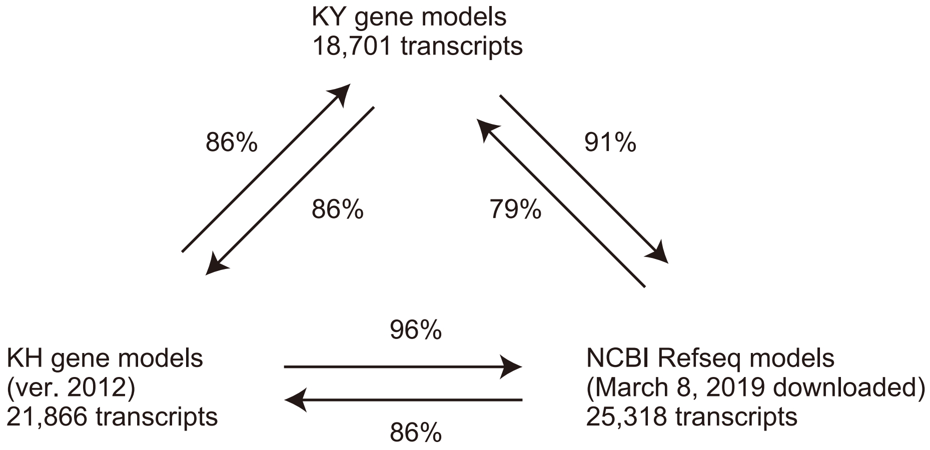

Supplementary figure S4. Comparisons among three gene model sets. These gene model sets were compared with BLASTN. Alignments longer than 300 bases and higher than 98% identity were considered to be significant. For shorter models (<300 bp), alignments above 90% coverage and higher that 98% identity were considered to be significant. Percentages of sequences with such alignments are shown.

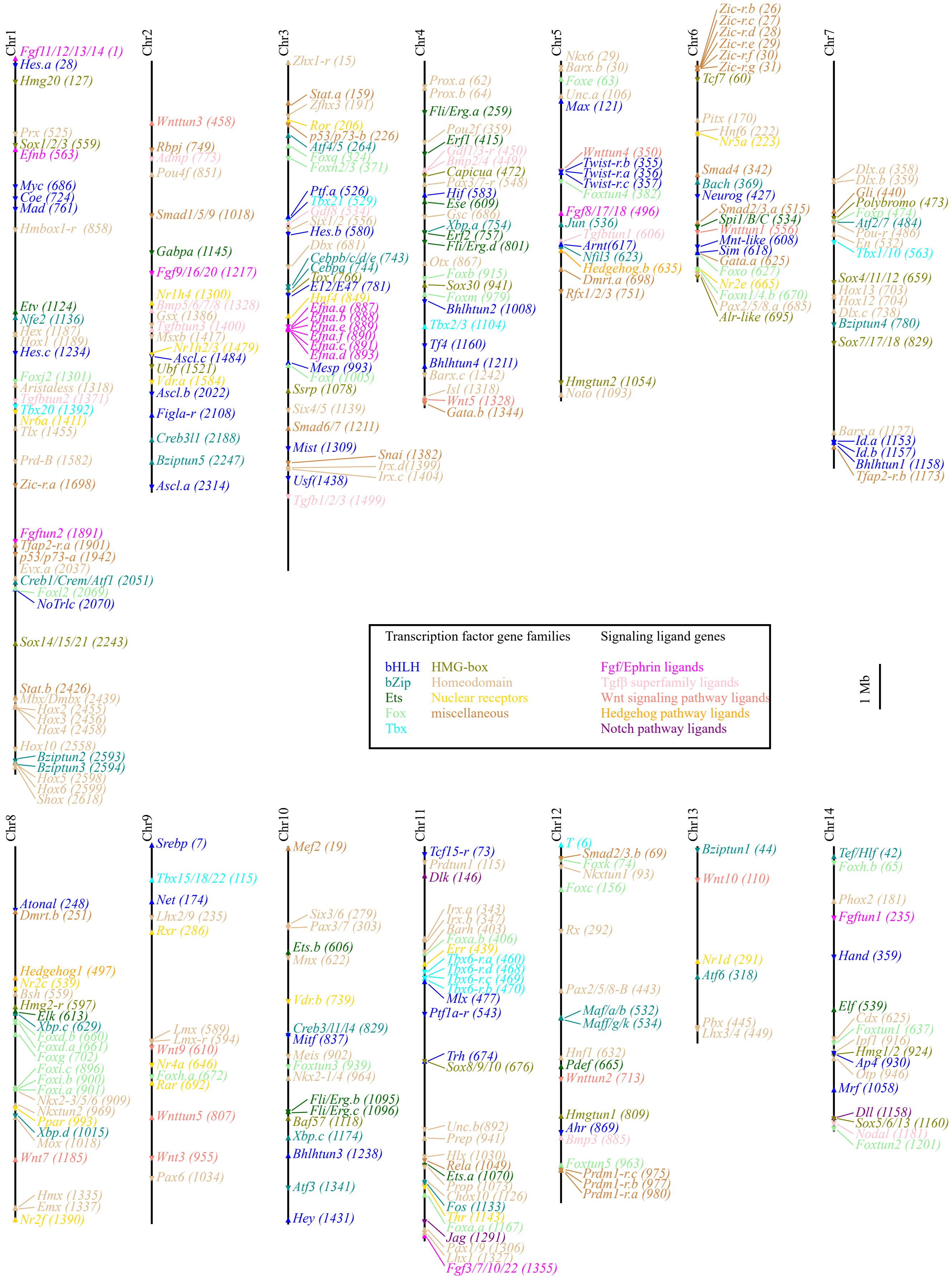

Supplementary figure S5. Chromosomal positions of several transcription family genes and several signaling molecule family genes. Gene families shown here (except the class miscellaneous) have been comprehensively annotated previously, and are indicated by different colors. The color code is in the middle of the figure. Numbers in parentheses indicate gene numbers for the KY gene model set. For example, the gene number for *Fgf11/12/13/14* is indicated as 1 on chromosome 1, and therefore the full gene code for *Fgf11/12/13/14* is KY.Chr1.1.

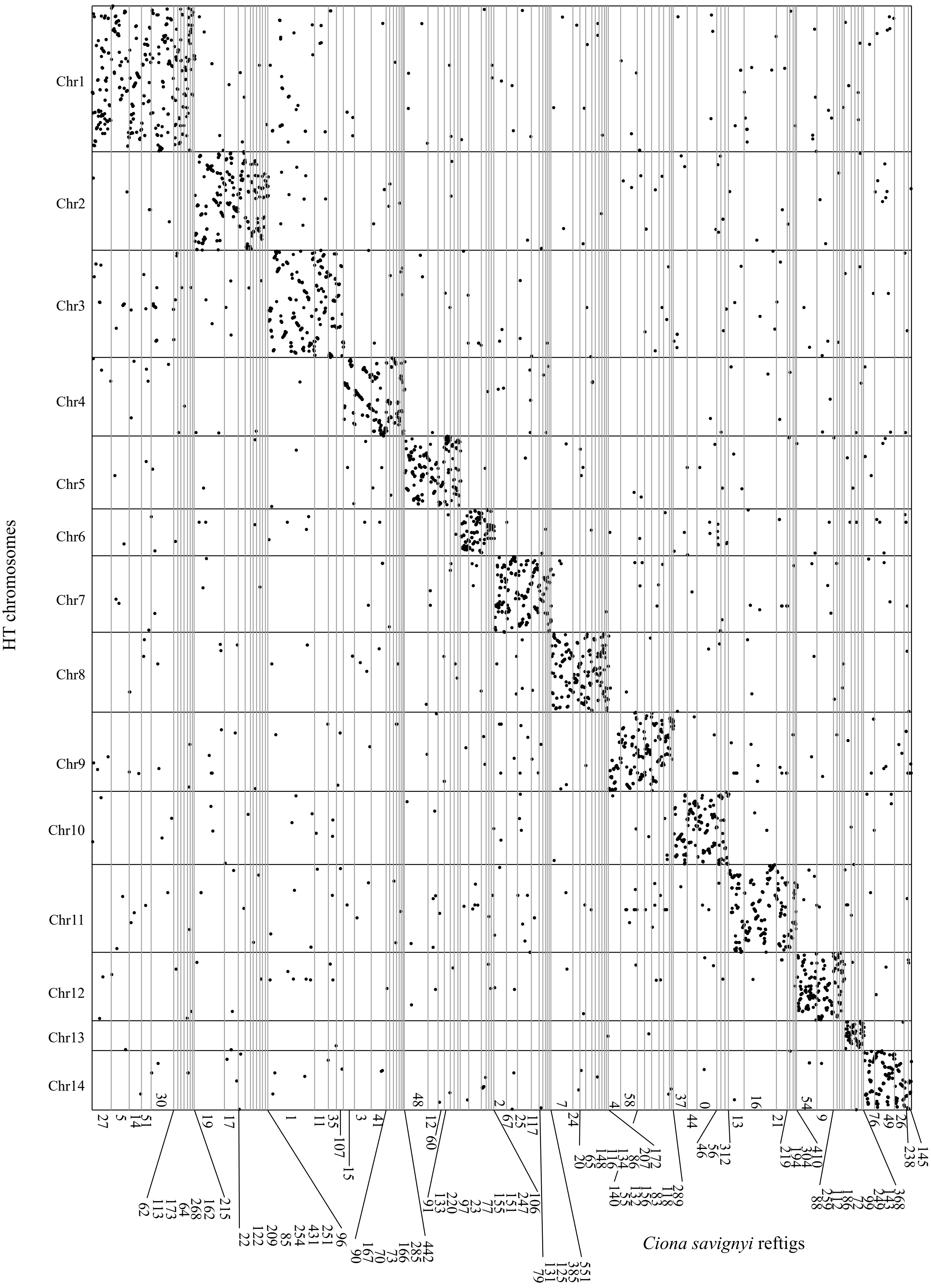

Supplementary figure S6. Extensive intrachromosomal rearrangements between two*Ciona* species. A dot plot represents the rank order position of orthologous gene pairs in the HT chromosomes and *C. savignyi* scaffolds (reftigs).

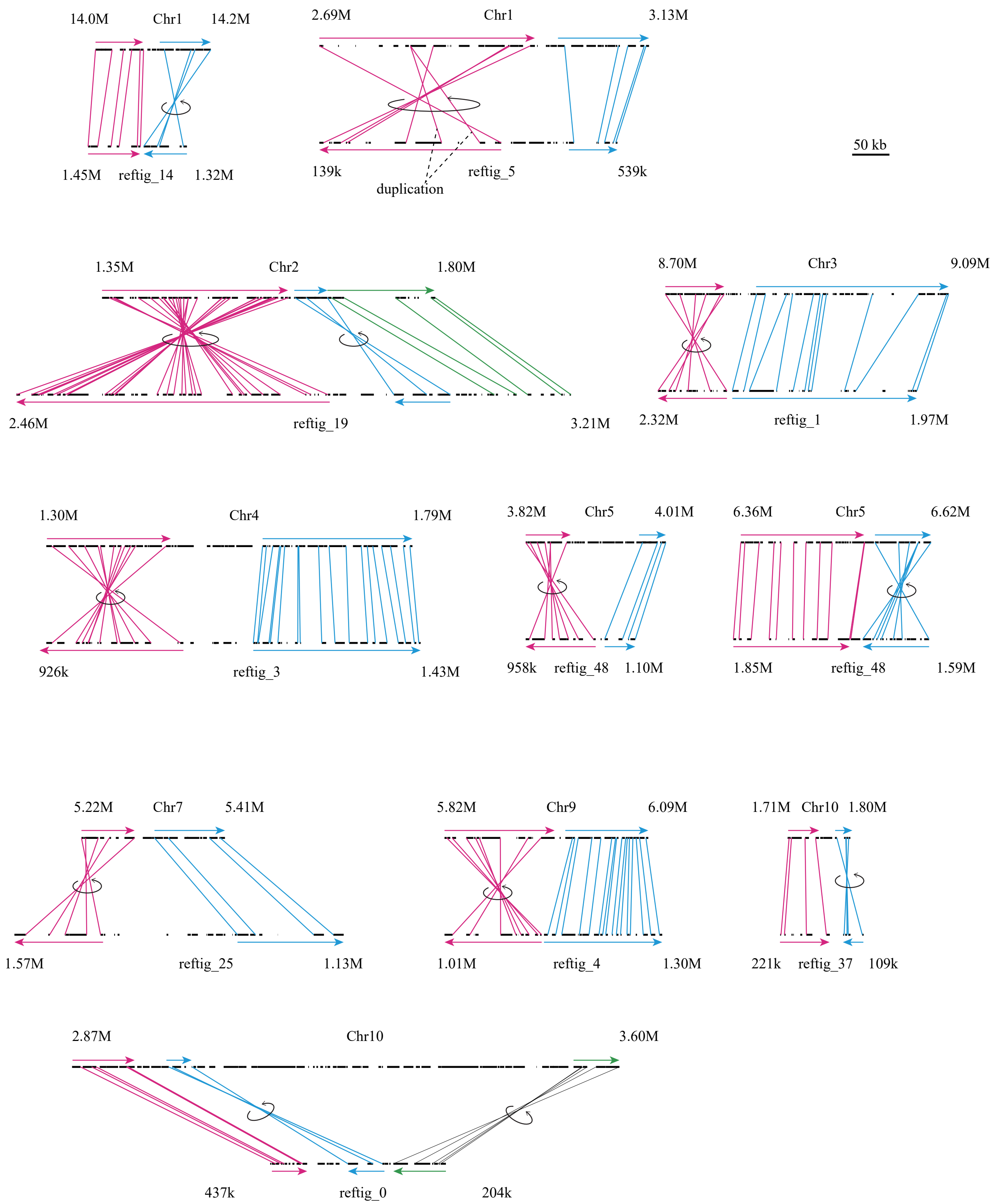

Supplementary figure S7. Eleven examples for inversions between two *Ciona* chromosomes. Genes are shown as black lines in the upper (HT chromosomes) and lower rows (*C. savignyi* reftigs) along the genomic regions shown above and below the rows. Genomic blocks showing co-linearity are indicated by colored arrows, and 5'-ends of putative orthologous genes are linked by lines of the same color. Putative inversions are indicated with black arrows.

Supplementary table S1. Evidence for assembling chromosomes

Chromosome	Nucleotide Position	Evidence
1	358,636-359,635	KH/Hi-C linked scaffolds
1	1,894,604-1,895,603	KH/Hi-C linked scaffolds
1	5,848,393-5,849,392	KH/Hi-C linked scaffolds
1	14,395,767-14,396,766	KH/Hi-C linked scaffolds
1	15,143,049-15,144,048	FISH (BAC clones: GECi40_o18/GECi23_n03)
2	5,179,745-5,180,744	KH/Hi-C linked scaffolds
2	5,664,628-5,665,627	KH/Hi-C linked scaffolds
2	6,353,340-6,354,339	KH/Hi-C linked scaffolds
2	7,268,078-7,269,077	KH/Hi-C linked scaffolds
2	8,946,064-8,947,063	FISH (BAC clones: GECi43_k20/GECi47_e09)
3	3,564,641-3,565,640	KH/Hi-C linked scaffolds
3	4,264,371-4,265,370	KH/Hi-C linked scaffolds
3	10,317,875-10,318,874	FISH (BAC clones: GECi41_o09/GECi39_k23/GECi46_g18)
4	58,726-59,725	KH/Hi-C linked scaffolds
4	6,903,212-6,904,211	KH/Hi-C linked scaffolds
4	7,127,593-7,128,592	KH/Hi-C linked scaffolds
5	5,859,989-5,860,988	KH/Hi-C linked scaffolds
5	6,342,749-6,343,748	KH/Hi-C linked scaffolds
5	6,875,472-6,876,471	FISH (BAC clones: GECi20_c18/GECi47_m12/GECi43_d04)
6	666,251-667,250	KH/Hi-C linked scaffolds
6	3,657,672-3,658,671	KH/Hi-C linked scaffolds
7	1,511,674-1,512,673	FISH (BAC clones: GECi20_e05/GECi36_j08)
7	4,312,916-4,313,915	KH/Hi-C linked scaffolds
7	4,897,647-4,898,646	KH/Hi-C linked scaffolds
8	1,122,451-1,123,450	FISH (BAC clones: GECi38_g14/GECi47_f07/GECi42_h16)
8	6,980,550-6,981,549	KH/Hi-C linked scaffolds
8	7,757,838-7,758,837	KH/Hi-C linked scaffolds
9	3,868,676-3,869,675	KH/Hi-C linked scaffolds
9	4,732,241-4,733,240	KH/Hi-C linked scaffolds
9	5,415,695-5,416,694	KH/Hi-C linked scaffolds

10	2,715,597-2,716,596	KH/Hi-C linked scaffolds
10	7,276,616-7,277,615	KH/Hi-C linked scaffolds
10	7,560,992-7,561,991	FISH (BAC clones: GECi39_p11/GECi30_h02/GECi27_m19)
11	2,941,714-2,942,713	KH/Hi-C linked scaffolds
11	3,496,081-3,497,080	KH/Hi-C linked scaffolds
11	3,598,093-3,599,092	KH/Hi-C linked scaffolds
12	3,142,657-3,143,656	KH/Hi-C linked scaffolds
12	6,890,859-6,891,858	KH/Hi-C linked scaffolds
13	1,105,076-1,106,075	KH/Hi-C linked scaffolds
14	3,044,729-3,045,728	KH/Hi-C linked scaffolds

Supplementary table S2. Validation of the HT assembly using FISH results for 270 BAC clones

BAC clone	Chromosome*	Order*	Genomic positions in the HT assembly
GECi43_d14	1	1	221 Kb
GECi41_f01	1	2	336 Kb
GECi43_d24	1	3	unmapped
GECi36_d24	1	4	1.3 Mb
GECi27_d06	1	5	2.5 Mb
GECi39_k06	1	6	2.9 Mb
GECi26_o02	1	7	3 Mb
GECi46_a05	1	8	3.4 Mb
GECi45_c11	1	9	5 Mb
GECi38_c18	1	10	5.3 Mb
GECi37_o16	1	11	5.7 Mb
GECi28_o13	1	12	6.1 Mb
GECi45_b24	1	13	6.3 Mb
GECi20_e16	1	14	6.4 Mb
GECi27_i10	1	15	6.6 Mb
GECi37_a23	1	16	7.1 Mb
GECi41_c08	1	17	7.6 Mb
GECi23_m14	1	18	8.6 Mb
GECi31_j07	1	19	8.7 Mb
GECi18_g12	1	20	9 Mb
GECi40_l15	1	21	9.3 Mb
GECi37_e22	1	22	10.4 Mb
GECi23_j14	1	23	10.7 Mb
GECi28_m19	1	24	11 Mb
GECi45_a01	1	25	12 Mb
GECi39_f18	1	26	12.3 Mb
GECi31_j11	1	27	12.6 Mb
GECi31_p02	1	28	12.8 Mb
GECi40_e06	1	29	13 Mb
GECi37_l17	1	30	13.3 Mb

GECi41_h20	1	31	14.2 Mb
GECi31_h16	1	32	15 Mb
GECi40_o18	1	33	unmapped
GECi23_n03	1	34	15.5 Mb
GECi27_l04	2	1	333 Kb
GECi46_h17	2	2	744 Kb
GECi38_k22	2	3	unmapped
GECi41_a01	2	4	1.2 Mb
GECi45_j15	2	5	2.3 Mb
GECi38_p05	2	6	2.7 Mb
GECi41_d18	2	7	4.4 Mb
GECi35_k18	2	8	4.6 Mb
GECi46_k01	2	9	4.9 Mb
GECi18_g15	2	10	5.5 Mb
GECi46_h13	2	11	5.8 Mb
GECi47_a06	2	12	6.1 Mb
GECi42_k02	2	13	6.4 Mb
GECi44_j14	2	14	7.1 Mb
GECi48_g02	2	15	7.4 Mb
GECi22_g17	2	16	7.8 Mb
GECi31_g10	2	17	8.1 Mb
GECi38_e18	2	18	8.5 Mb
GECi24_f14	2	19	unmapped
GECi43_k20	2	20	9.2 Mb
GECi47_e09	2	21	9.4 Mb
GECi46_l15	3	1	15 Kb
GECi31_c13	3	2	unmapped
GECi46_h21	3	3	1 Mb
GECi46_d23	3	4	1.4 Mb
GECi23_j02	3	5	1.8 Mb
GECi37_i18	3	6	1.9 Mb
GECi27_h01	3	7	2.1 Mb
GECi28_d04	3	8	2.9 Mb
GECi40_c20	3	9	3.2 Mb
GECi31_b03	3	10	3.5 Mb

GECi32_c01	3	11	4 Mb
GECi45_b21	3	12	4.6 Mb
GECi26_o05	3	13	4.9 Mb
GECi29_j07	3	14	5.2 Mb
GECi44_a17	3	15	5.3 Mb
GECi42_m21	3	16	6.7 Mb
GECi35_m19	3	17	7.2 Mb
GECi30_e01	3	18	7.4 Mb
GECi41_j13	3	19	7.8 Mb
GECi40_n17	3	20	8.7 Mb
GECi35_g08	3	21	9 Mb
GECi23_n22	3	22	9.5 Mb
GECi20_c16	3	23	9.6 Mb
GECi46_d19	3	24	10.1 Mb
GECi41_o09	3	25	10.8 Mb
GECi39_k23	3	26	11 Mb
GECi46_g18	3	27	11.1 Mb
GECi27_g06	4	1	440 Kb
GECi47_m18	4	2	602 Kb
GECi46_b22	4	3	905 Kb
GECi28_j12	4	4	1.2 Mb
GECi31_h10	4	5	1.6 Mb
GECi39_d17	4	6	3.2 Mb
GECi47_a17	4	7	3.7 Mb
GECi38_g13	4	8	5 Mb
GECi44_c14	4	9	5.3 Mb
GECi41_c18	4	10	6.5 Mb
GECi46_h16	4	11	7 Mb
GECi48_b03	4	12	7.5 Mb
GECi26_p05	5	1	270 Kb
GECi40_e21	5	2	661 Kb
GECi40_k16	5	3	866 Kb
GECi46_m20	5	4	1.1 Mb
GECi35_j24	5	5	1.4 Mb
GECi47_l17	5	6	2.5 Mb

GECi46_k04	5	7	2.6 Mb
GECi47_h08	5	8	3.5 Mb
GECi31_h15	5	9	4 Mb
GECi42_d18	5	10	3.9 Mb
GECi46_g06	5	11	5.2 Mb
GECi39_d16	5	12	5.4 Mb
GECi30_g04	5	13	5.8 Mb
GECi38_b03	5	14	6.2 Mb
GECi20_c18	5	15	7 Mb
GECi47_m12	5	16	7.2 Mb
GECi43_d04	5	17	7.4 Mb
GECi35_o20	6	1	93 Kb
GECi45_o09	6	2	522 Kb
GECi27_m12	6	3	789 Kb
GECi19_e01	6	4	1.4 Mb
GECi40_j01	6	5	1.4 Mb
GECi48_n14	6	6	unmapped
GECi46_b01	6	7	1.7 Mb
GECi28_e05	6	8	2 Mb
GECi26_m10	6	9	2.6 Mb
GECi36_o01	6	10	3.1 Mb
GECi35_i11	6	11	3.4 Mb
GECi40_c13	6	12	3.9 Mb
GECi48_g11	6	13	4.5 Mb
GECi18_e21	6	14	4.6 Mb
GECi20_e05	7	1	818 Kb
GECi36_j08	7	2	1.2 Mb
GECi46_l20	7	3	1.6 Mb
GECi15_k04	7	4	1.7 Mb
GECi48_g08	7	5	2.8 Mb
GECi13_m09	7	6	3.3 Mb
GECi17_i16	7	7	3.8 Mb
GECi44_o12	7	8	unmapped
GECi22_c14	7	9	unmapped
GECi28_i19	7	10	5.3 Mb

GECi37_e03	7	11	5.8 Mb
GECi42_e19	7	12	6.1 Mb
GECi20_i01	7	13	6.7 Mb
GECi41_b01	7	14	7.3 Mb
GECi47_h12	7	15	8.4 Mb
GECi40_p19	7	16	8.6 Mb
GECi36_a07	7	17	8.8 Mb
GECi38_g14	8	1	594 Kb **
GECi47_f07	8	2	852 kb **
GECi42_h16	8	3	1 Mb
GECi41_f18	8	4	unmapped
GECi36_m09	8	5	1.2 Mb
GECi41_c19	8	6	1.3 Mb
GECi24_a14	8	7	1.4 Mb
GECi28_f10	8	8	1.5 Mb
GECi31_c16	8	9	2.5Mb ***
GECi38_g11	8	10	2.6 Mb
GECi42_a15	8	11	3 Mb
GECi39_b24	8	12	3.2 Mb
GECi38_i23	8	13	3.2 Mb
GECi38_d12	8	14	3.4 Mb
GECi39_a23	8	15	4 Mb
GECi47_c08	8	16	4.4 Mb
GECi48_c22	8	17	4.6 Mb
GECi39_p17	8	18	4.8 Mb
GECi13_k10	8	19	4.8 Mb
GECi38_b08	8	20	5.4 Mb
GECi27_m09	8	21	6 Mb
GECi42_j18	8	22	6.9 Mb
GECi23_a18	8	23	7.2 Mb
GECi31_b20	8	24	7.6 Mb
GECi31_p24	8	25	7.9 Mb
GECi31_b13	8	26	8.2 Mb
GECi41_l21	9	1	270 Kb
GECi16_d18	9	2	305 Kb

GECi37_l23	9	3	454 Kb
GECi40_g03	9	4	1.3 Mb
GECi30_d14	9	5	1.7 Mb
GECi31_g18	9	6	3.3 Mb
GECi17_i24	9	7	3.7 Mb
GECi40_c01	9	8	4 Mb
GECi17_f22	9	9	4.4 Mb
GECi38_j10	9	10	4.6 Mb
GECi23_g02	9	11	4.9 Mb
GECi44_k03	9	12	5.2 Mb
GECi42_e12	9	13	5.8 Mb
GECi17_p17	9	14	6 Mb
GECi45_n13	9	15	6.5 Mb
GECi41_e15	9	16	6.9 Mb
GECi27_o10	9	17	7.7 Mb
GECi46_n10	9	18	8.2 Mb
GECi22_b17	10	1	486 Kb
GECi43_p13	10	2	738 Kb
GECi26_m14	10	3	1.2 Mb
GECi15_i18	10	4	2.1 Mb
GECi46_k15	10	5	2.6 Mb
GECi38_f04	10	6	3.1 Mb
GECi32_a09	10	7	3.6 Mb
GECi14_l18	10	8	4 Mb
GECi38_e13	10	9	4.4 Mb
GECi16_c03	10	10	4.6 Mb
GECi42_k13	10	11	4.9 Mb
GECi30_f09	10	12	5.6 Mb
GECi27_g17	10	13	6.2 Mb
GECi15_d02	10	14	6.6 Mb
GECi38_a19	10	15	7.1 Mb
GECi41_a08	10	16	7.4 Mb
GECi39_p11	10	17	8 Mb
GECi30_h02	10	18	8.1 Mb
GECi27_m19	10	19	8.3 Mb

GECi46_i05	11	1	219 Kb
GECi36_a08	11	2	650 Kb
GECi28_m24	11	3	1.5 Mb
GECi39_o18	11	4	1.8 Mb
GECi38_c09	11	5	1.9 Mb
GECi48_n17	11	6	2.4 Mb
GECi28_a19	11	7	2.6 Mb
GECi40_g04	11	8	3 Mb
GECi22_f05	11	9	2.8 Mb
GECi46_o22	11	10	3.1 Mb
GECi45_b12	11	11	3.3 Mb
GECi26_d15	11	12	3.8 Mb
GECi40_a09	11	13	4.2 Mb
GECi15_b02	11	14	unmapped
GECi46_d13	11	15	5.3 Mb
GECi37_i12	11	16	5.7 Mb
GECi28_m06	11	17	5.9 Mb
GECi28_p08	11	18	7.7 Mb
GECi46_i11	11	19	8.1 Mb
GECi36_d13	11	20	8.3 Mb
GECi44_e14	11	21	8.6 Mb
GECi31_f08	11	22	8.6 Mb
GECi45_a22	12	1	84 Kb
GECi35_b12	12	2	426 Kb
GECi47_j16	12	3	1.2 Mb
GECi18_c13	12	4	2.1 Mb
GECi40_n04	12	5	2.3 Mb
GECi25_m07	12	6	2.4 Mb
GECi24_f21	12	7	3.3 Mb
GECi20_e08	12	8	3.5 Mb
GECi45_o05	12	9	4.5 Mb
GECi40_k03	12	10	5.5 Mb
GECi46_e22	12	11	5.7 Mb
GECi27_e17	12	12	6 Mb
GECi16_k11	12	13	6.2 Mb

GECi21_a11	12	14	6.5 Mb
GECi36_b17	12	15	7 Mb
GECi39_c09	12	16	7.3 Mb
GECi27_c10	12	17	7.7 Mb
GECi20_b17	13	nd	unmapped
GECi23_l17	13	nd	817 Kb
GECi46_e02	13	nd	235 Kb
GECi13_p09	13	1	unmapped
GECi31_b04	13	2	unmapped
GECi31_c22	13	3	3.2 Mb
GECi46_d01	13	4	3.7 Mb
GECi18_e13	13	5	4.3 Mb
GECi17_n22	13	6	4.2 Mb
GECi41_b16	14	1	83 Kb
GECi38_j05	14	2	426 Kb
GECi44_g03	14	3	658 Kb
GECi22_e15	14	4	1.3 Mb
GECi40_e22	14	5	1.8 Mb
GECi44_d13	14	6	2.8 Mb
GECi28_p10	14	7	2.9 Mb
GECi13_l12	14	8	3.2 Mb
GECi48_m01	14	9	3.7 Mb
GECi42_m08	14	10	4.1 Mb
GECi48_j16	14	11	4.3 Mb
GECi36_o02	14	12	4.5 Mb
GECi17_i09	14	13	4.7 Mb
GECi27_g03	14	14	4.9 Mb
GECi40_k08	14	15	5.2 Mb
GECi44_g24	14	16	5.6 Mb
GECi39_l14	14	17	6.1 Mb

* The orders of BAC clones on individual chromosomes are reported in Shoguchi (2006).

** One end sequence of each of these two clones was mapped to the designated location, while the other end was mapped to unassembled contigs.

*** One end sequence of GECi31_c16 was mapped to genomic position 2.5 Mb in chromosome 8, while the other end was mapped to genomic position 3.0 Mb in chromosome 10.

Supplementary table S3. Putative telomeric repeats in chromosomal ends.

Chromosomes	Number of 'CCCCTAA' in the initial 2 kb region	Number of 'CCCCTAA' in the last 2 kb region
Chr1	0	0
Chr2	0	0
Chr3	178	260
Chr4	0	151
Chr5	0	212
Chr6	0	99
Chr7	0	151
Chr8	0	0
Chr9	181	207
Chr10	0	157
Chr11	0	0
Chr12	167	1
Chr13	0	217
Chr14	111	130
